# Nucleotide Loading Modes of Human RNA Polymerase II as Deciphered by Molecular Simulations

**DOI:** 10.3390/biom10091289

**Published:** 2020-09-07

**Authors:** Nicolas E. J. Génin, Robert O. J. Weinzierl

**Affiliations:** 1 Institut de Chimie Organique et Analytique, Université d’Orléans, 45100 Orléans, France; nicolas.genin@univ-orleans.fr; 2 Department of Life Sciences, Imperial College London, London SW7 2AZ, UK

**Keywords:** RNA polymerase, nucleoside triphosphate, main channel, secondary channel, tertiary channel, downstream bubble, loading, diffusion, entry, TFIIF

## Abstract

Mapping the route of nucleoside triphosphate (NTP) entry into the sequestered active site of RNA polymerase (RNAP) has major implications for elucidating the complete nucleotide addition cycle. Constituting a dichotomy that remains to be resolved, two alternatives, direct NTP delivery via the secondary channel (CH2) or selection to downstream sites in the main channel (CH1) prior to catalysis, have been proposed. In this study, accelerated molecular dynamics simulations of freely diffusing NTPs about RNAPII were applied to refine the CH2 model and uncover atomic details on the CH1 model that previously lacked a persuasive structural framework to illustrate its mechanism of action. Diffusion and binding of NTPs to downstream DNA, and the transfer of a preselected NTP to the active site, are simulated for the first time. All-atom simulations further support that CH1 loading is transcription factor IIF (TFIIF) dependent and impacts catalytic isomerization. Altogether, the alternative nucleotide loading systems may allow distinct transcriptional landscapes to be expressed.

## 1. Introduction

**Figure 1:**
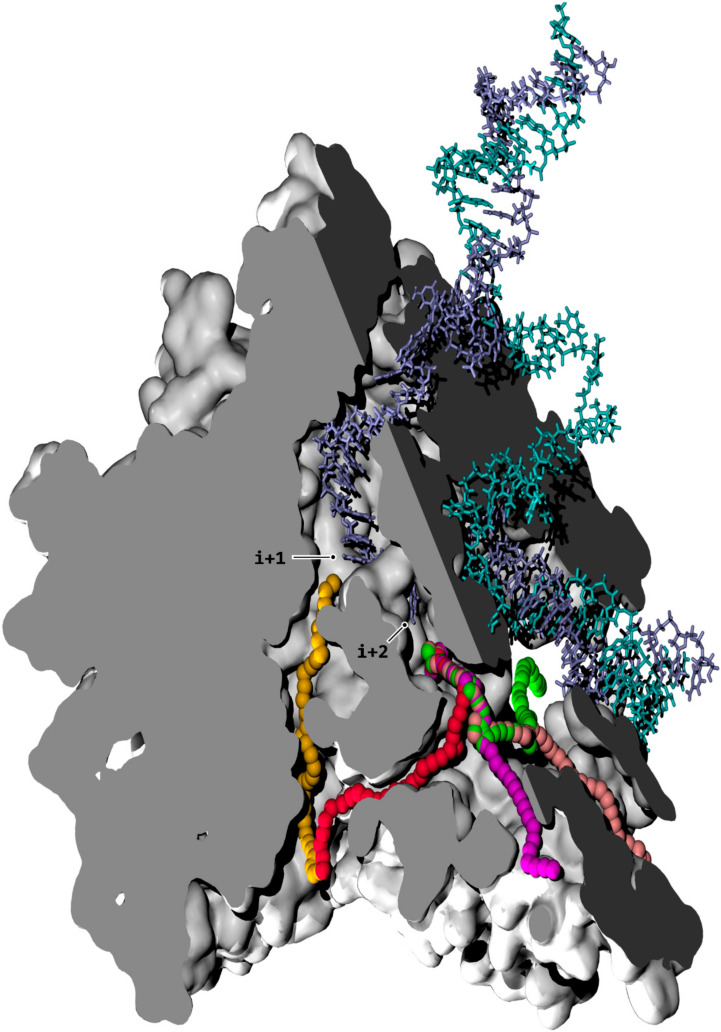
Nucleoside triphosphate (NTP) pathways in RNAPII. The cutaway view through the enzymatic complex is rendered via two cutoff planes (light and dark grey solid fills). The displayed conformation is from simulation aMD_A1 (simulation indexes are listed in Table 1). The protein walls are represented as white surfaces. The template and non-template DNA strands are fully shown, in fade blue and cyan stick representation, respectively. The pathway axes verifying maximal distance from protein interlining atoms for secondary channel (CH2) (orange), CH3A (red), CH3B (magenta), CH3C (pink), and CH3D (green) subchannels are shown as a series of balls. The latter trajectories are generated with a custom-built pathway-exploration algorithm. The CH2 trajectory leads to the i + 1 register, whereas the CH3A/B/C/D paths lead to the i + 2 position.

RNA polymerases (RNAPs) are nanoscopic machines responsible for transcribing sections of the information stored in DNA into RNA. In all cellular RNAPs, the catalytic site carrying out the RNA synthesis reactions is buried deep within a large multi-subunit protein complex. Among several mechanistic requirements, RNAPs possess an active site sequestered at the center of the enzyme to facilitate the coupling of polymerization with catalytic dehydration and to maintain a precise structural geometry around the active site pocket. Entry of the nucleic acids and polymerization substrates (nucleoside triphosphates, NTPs) occurs through various channels that link the interior of the enzyme to the intracellular environment. An overview of access channels into RNAPII is shown in Figure 1.

The secondary channel (CH2, shown in orange in Figure 1) starts at a large conical section (funnel), and then converges to a narrower pore (corridor) that leads directly to the active site of RNAP. The CH2 hypothesis, i.e., delineating a possible pathway allowing NTPs to access the catalytic register of the DNA template strand in the catalytic center, was initially based on the first generated structures of bacterial RNAP and eukaryotic RNAPII, where CH2 was identified as the only unobstructed active site leading route [1,2]. Several lines of computational and experimental evidences support the latter hypothesis. The existence of an entry site (E site), which is able to bind nucleotides in the corridor, suggests CH2 as the most obvious access point to this location [3,4,5,6,7,8]. Molecular dynamics (MD) simulations of NTP diffusion substantiate that CH2 is compatible with the observed in vivo synthesis rate [9,10], whereas alternative pathways do not appear to satisfy conformational and electrostatic requirements for NTP loading [11]. Finally, the CH2 model correlates well to the more general nucleotide addition cycle. Direct access of single NTPs to the catalytic site via CH2 offers, indeed, the simplest explanation for transcription proceeding unidirectionally, while accommodating translocation oscillations (Brownian ratchet model) [12,13,14]. A series of biochemical studies conducted on bacterial RNAP and human RNAPII uncovered a fascinating alternative loading mechanism. In this model, RNAP can be seen as a factory production chain, where the substrates are lined up in the main channel (CH1) and stored within the enzymatic complex prior to catalysis. CH1 is a cleft-like structure forming an elbow-shaped corridor through the center of RNAP, and its predominant function is to allow the entry of DNA in RNAPs from all three domains of life (archaea, bacteria, and eukaryotes). Experimental data support that the pathway can also accommodate substrate binding to downstream DNA at the i + 2, i + 3, or even up to the i + 4 registers, acting as secondary templated sites, before NTPs are incrementally shifted to the catalytic site. This concept was based on compelling evidence comparing the fate of i + 1 (or subsequent i + 2) nucleotide with and without preincubation of the next templated nucleotides [15,16,17,18,19]. Additional lines of evidence included the sigmoidal dependence of the reaction, or pause escape rate, on substrate concentrations and global kinetic fits to a model, where i + 2 NTP accelerated state transitions [15,16,20,21,22,23]. Kinetic data indicate that TFIIF is required for secondary NTP induced allostery to take place in RNAPII [20,21,22]. Although based on persuasive functional data, it has been challenging to reconcile the CH1 hypothesis with the available structural material, as tight protein electrostatic contacts with the downstream DNA helix did not appear to leave room for NTPs to enter alongside.

The evidence supporting direct delivery of individual NTPs to the active site via CH2 and the observations that NTPs can bind to several positions along the DNA template strand before transferring into the active site appear to be in contradiction. Mapping the NTP entry routes constitutes an arduous investigation because the diffusion process occurs in the sub-millisecond range and is an intrinsically dynamic process. Traditional experimental techniques, offering either limited spatial (e.g., transient state kinetics) or temporal (e.g., X-ray crystallography) resolutions, struggle to grasp the full mechanistic overview of the loading mechanism. In the following investigation, we discuss structural evidence suggesting the existence of a yet undescribed third NTP entry channel (CH3, Figure 1, shown in red and other colors), supplying access to CH1 downstream bubble. The combined results from accelerated molecular dynamics (aMD) simulations of NTP diffusion into RNAP through these two channels provide new insights into the substrate loading mechanisms governing transcription elongation.

## 2. Materials and Methods

Accelerated molecular dynamics (aMD) simulations were computed with the dual boost method [24], and run with the CUDA (Compute Unified Device Architecture) mixed precision model [25]. Dihedral boost α and potential were calculated as 4.5 kcal/mol per protein residue and median dihedral potential added to one-fifth of α. Total boost α and potential parameters were defined as 0.20 kcal/mol per atom and median total parameters potential added to α. Structural alignments were carried out with Visual Molecular Dynamics 1.9.2 [26]. Insertion of missing loops and residue mutations were performed with Yasara Structure [27]. System preparation and minimization were performed with the Amber 16 package [28]. Water, DNA, RNA, and protein amino acid models were the following: TIP4PEW, DNA.OL15, RNA.OL3, and ff14SB [29,30,31,32,33,34,35,36,37]. The DNA forcefield was based on a modified parameter set [38]. Divalent Mg^2+^ and monovalent K^+^, Na^+^, Cl^–^ ionic parameters included a third term in the van der Waals potential accounting partially for polarizability (12-6-4 forcefield) [39,40,41]. Although the 12-6-4 parameters display superior physical description of strongly charged atoms, a slight overestimation of short-range van der Waals interactions can occur, thereby overlapping interacting atoms below their van der Waals surface, because of the intrinsic limitations of non-fully polarizable forcefields. At the start of the simulation runs, if a nucleotide attached magnesium atom was trapped by aspartate OD1/OD2 atoms, glutamate OE1/OE2 atoms, or template i + 3 guanine OP1/OP2 atoms (collapse of the van der Waals radii characterized by an interatomic distance below 2 Å), the divalent Mg^2+^ forcefield was reverted back to standard 12-6 parameters without the r^−4^ term. Such a parametrization choice provided the best compromise between the sole utilization of the default 12-6 or 12-6-4 potentials, and was motivated by the observation of an improved NTP diffusion process (e.g., overall decreased propensity of NTP aggregates formation), whilst preventing prolonged trapping (energetic well) of the NTP-bound Mg^2+^ by oxygen atoms during diffusion. The NTP molecule used in the simulations was a cytidine triphosphate (CTP). CTP parameters were provided from a previous study [42,43]. The initial system was the 10-subunit human RNAPII, extracted from PDB#5IY9 [44], with the TFIIF subunit RAP74 extended to amino acid position 227 (modeled with I-Tasser) [45], the RNA chain extended to i − 16, and the double-stranded DNA ranged from i − 30 to i + 20. The template and non-template i + 1 to i + 4 positions were mutated to guanine and cytosine, respectively. The initial structure was drawn from a preinitiation complex in the starting transcribing state. The latter structure was the only currently available X-ray/cryo-EM-derived RNAPII complex with a complete transcription bubble and an associated TFIIF molecule. The fact that the structure is a preinitiation complex provides an enhanced resolution of critical domains that are seldom stable enough to allow atomic mapping, such as the full length of the non-template DNA strand and the two subunits of TFIIF, due to the presence of a large array of stabilizing preinitiation proteins. Because the RNAPII complex was resolved in the starting transcribing state, the double DNA helix, together with the RNA transcript, were appropriately engaged in the enzyme. Furthermore, once stripped of its preinitiation proteins, the RNAPII-nucleic structure was virtually identical to an elongation complex (RMSD of 1.50 Å, Figure S1), indicating that the model was suitable to investigate the elongation stage of the transcription cycle. The starting model was embedded in a water box extending an additional 15 Å in x, y, and z dimensions from the protein. The box also contained 15 mM Mg^2+^ bound CTPs, 15 mM Na^+^, and 150 mM K^+^ and Cl^−^ concentration to ensure charge neutrality. The latter compounds were randomly placed in the solvation box with the AddToBox module of Amber 16. The downstream and upstream DNA terminal base pairs were stabilized through the addition of harmonic bond restraints between the hydrogen bond forming atoms. Charged ends were added at the amino-terminal (N-TER) and carboxy-terminal (C-TER) portions of the respective subunits, except for C-TER of RPB1, N-TER of RPB6, and C-TER of TFIIF subunit RAP74, which were appropriately capped with either acetyl- or N-methylamide groups. Histidine residue RPB1 1108 was protonated based on a previous study [46]. Simulations containing a closed trigger loop were aligned into PDB#5IY9 using PDB#2E2H as a template [5]. The simulations were carried out with OpenMM 7.0 [47,48,49,50,51]. Seventy-seven aMD simulations ranging from 10 to 370 ns (Table 1) were run to investigate NTP loading into RNAPII.

The simulations were run several times from the same input to explore landscape variants and were optionally supplemented with specific NTP diffusional states (referred to as “transitive NTP”, Table 1) in order to capture forward sampling along a given pathway. Simulations aMD_B, B’, C, and D specifically investigated loading through CH1, aMD_K, and L through CH2, aMD_E, F, and F’ isomerization in CH1, and aMD_G, H, I, and J shuttling of i + 2 CTP to the i + 1 position. Simulations aMD_M compared the effect of multiple downstream bindings in CH1. Simulations aMD_A to M simultaneously were designed to investigate overall substrate diffusion by including 15 mM CTP available in the solvent buffer. According to an alternative hybrid aMD/steered MD method, simulations aMD_N compared substrate input via the different CH1 entry paths and involved the pulling of an NTP with a 0.03 kcal/mol Å^−2^ external force and the single boost acceleration of the conformational dynamics. Molecular renderings presented in this article were produced with Visual Molecular Dynamics 1.9.2 and associated packages: SURF accessible surface calculator [52], STRIDE Secondary Structure Prediction [53], Persistence of Vision Raytracer (Version 3.6), and the Tachyon ray tracing library [54]. A pathway-exploration algorithm designed to calculate iteratively a series of points to verify the optimal distance from surrounding protein atoms was custom written in Perl. Then, the latter points were used to extract the minimal radii along the pathway axis. The detailed simulation procedures are available in Appendix A. Amino acids are referred to using the following nomenclature: The subunit name for sequences of amino acids is either listed explicitly for a continuous range of residues (e.g., RPB1 1100–1110) or, for individual amino acids, indicated as a superscript before the amino acid type letter code (e.g., **^1^**H1108 for RBP1 histidine residue number 1108). 

Collectively, the simulation data presented in this article should be considered to be semiquantitative. The reported timescales and probabilities of the observables do not reflect the absolute time evolution of the configurational space but, instead, are used to describe the metastable states of the protein and the NTP ligand dynamics. The aMD protocol applies a boost to the potential energy surface, effectively enhancing the sampling of the conformational space and the transition between energetic basins. Compared to nonenhanced sampling techniques (such as conventional MD), aMD promotes the sampling of rare events (low-energy state transitions), and compared to other enhanced sampling procedures, the aMD method benefits from not relying on an a priori reaction-coordinate knowledge. For large biomolecular systems such as the enzymatic complex investigated in this article, an accurate reweighting of the trajectories to recover the canonical ensemble averages can be hampered by statistical errors and the transmission coefficient remains ill-defined to recover the nonbiased timescale of events [55,56]. The “Gaussian” aMD method has recently been developed and shows the ability to achieve a more robust reweighting of systems with a large number of atoms. The latter aMD implementation improves the derivation of well-converged free energy profiles by leveraging cumulant expansion to the second order with a boost potential following a Gaussian distribution [57]. Un-reweighted aMD simulations are nevertheless useful to derive a semiquantative ranking of the observable probabilities [58,59], thereby capturing meaningful insights into the conformational and diffusional dynamics.

## 3. Results

### 3.1. TFIIF Reconfiguration of the Transcription Bubble

**Figure 2:**
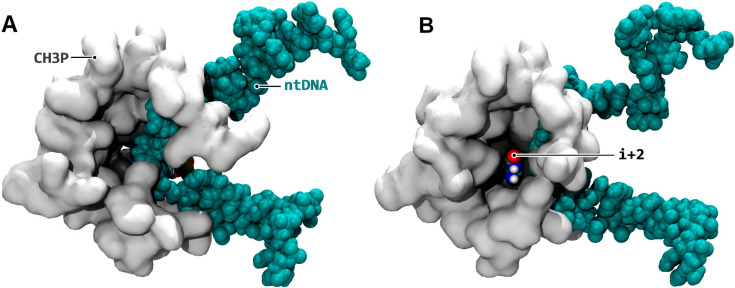
TFIIF-induced trapping of the i + 2 downstream register. (**A**) Configuration before (simulation aMD_A1, 15 ns); (**B**) After (aMD_A1, 220 ns) the relaxation of TFIIF. The folding of non-template DNA (**B**) frees up a cavity inside the protein represented as white surface, which in turn allows the template i + 2 register (colored by atomic type) to insert inside the cavity. The amino acids (RPB1 852, 853, 855, 856, and 859; RPB2 493–496 and 499) locking the i + 2 position are represented as grey surfaces.

TFIIF is a general transcription factor known to participate in the transcription initiation phase and is also predicted to impact the conformation of the RNAPII complex during elongation [20,21,22]. Figure 1 displays four unobstructed solvent-accessible paths to the exposed downstream i + 2 register when TFIIF actively rearranges the downstream bubble, a process detailed hereafter. In simulations aMD_A1 to 6 (the simulation indexes aMD_A to N described in this article are listed in Table 1) of the initial equilibration of the RNAPII-TFIIF complex, a significant remodeling of the transcription bubble was observed as follows: TFIIF interacted mainly with non-template DNA (ntDNA) positions i + 14 to i + 12 and i − 7 to i − 29 and with template DNA i − 30 to i − 8 and i + 8 to i + 12 registers (weakly with i − 9 and i − 23 to i − 27). The latter interaction was carried out through six articulated modules (Figure S2A,B). Module 1 (RAP74 166 and 169–172) is localized at the central section of the charged α1 helix (RAP74 152–186); Module 2 (RAP74 67–92) spans across the arm (RAP74 49–62 and 73–97) and insertion 1 (RAP74 62–73) domains; Modules 3 (RAP30 192–201), 4 (RAP30 223–234), and 5 (RAP30 209–212, 214, and 218) belong to the RAP30 winged helix (RAP30 176–249); Module 6 (RAP30 154–177 and 180) of the linker domain (RAP30 119–176) is adjacent to the winged helix. The observed rearrangement of the different DNA-interacting modules was the following: Module 1 anchored the downstream DNA helix through ntDNA i + 12 to i + 14 positions. Module 2 folded against Modules 3 and 4, which constricted upstream ntDNA about the i − 9 to i − 16 positions. The conjunction of the upstream strain induced by TFIIF Modules 2 to 4 on one side, with the downstream anchor provided by Module 1 on the other side, resulted in folding of ntDNA in between these modules (Figure S2C,D). Modules 5 and 6 interacted with the exiting reannealed DNA helix and the upstream template strand, respectively, which provided additional contacts to stimulate DNA remodeling. The folding of the non-template strand, observed most significantly around i + 2 to i − 6 positions, freed up a cavity inside the downstream bubble, where the template i + 2 register then inserted (Figure 2).

In simulations aMD_B to M, the i + 2 register remains predominantly locked inside the cavity between the fork loop 2 and bridge helix domains (Table S1). The remodeling of DNA inside RNAPII is accompanied by the reshaping of a large portion of the entire CH1 structure. The jaw and lobe domains close against and around the downstream helix, while the clamp stabilizes into close conformation (Figure S3). 

### 3.2. NTP Accessible Pathways

Up to six intra-enzymatic pathways are identified when CH1 is open, that is, when the jaw, the lobe, or the clamp do not tightly encircle the downstream helix, and five pathways are identified in the presence of TFIIF. In addition to the well-documented CH2 pathway, an overlooked branched route connecting the interior of the enzyme was identified. This pathway provided access deep into CH1 and displayed constant opening in all simulations. This supports the interpretation that it constitutes a genuine solvent accessible route, rather than an occasionally present structural feature. We refer to this structure as the tertiary channel (CH3) to maintain consistent nomenclature of channels in RNAPs. The CH3 consists of four subchannels, i.e., CH3A–D. The CH2 and CH3A/B/C/D pathways are illustrated in Figure 1 and the sequence of their respective exposed atoms are listed in Table S2. Similar to the CH2 pathway, CH3A originates from the funnel, but emerges to a location that corresponds to the “pore 2” structure. Pore 2 was briefly described in the original publications of the yeast RNAPII structure [60,61], but has apparently not been referred to since. CH3B is directly adjacent to CH3A, but its entrance does not lie in the funnel and is positioned more externally. The CH3B opening runs between the RPB9 zinc ribbon 2 domain and the jaw domain. The CH3C subchannel runs approximatively parallel to the downstream DNA helix through the center of the jaw domain and is adjacent to the CH3B subchannel. The CH3D subchannel is located underneath the downstream DNA helix, adjacent to the clamp, and connects to the CH3C subchannel orthogonally. The four subchannels merge into a cavity accommodating the i + 2 locked register. The latter cavity is referred to as the CH3 pocket (CH3P). The TFIIF folding improves the demarcation of the CH3C subchannel by collapsing protein residues along its axis through the closure of the jaw domain (Figure S3A,B). It is worth mentioning that an explanation for overlooking the CH3A/B/C/D subchannels in a previous MD investigation [11] could be the fact that the absence of TFIIF did not allow the necessary remodeling of the downstream bubble to expose the i + 2 template register inside CH3P. Finally, a sixth pathway, “CH4”, is identified, but it only connects to the inside of the enzyme when the CH1 pathway is open, hence, it only applies when TFIIF is not present, and therefore does not appear to constitute a possible substrate input path, as the i + 2 register would not be locked. The CH4 pathway originates on the opposite site of downstream DNA from the CH3C subchannel, between the lobe and the clamp, and then runs between the rudder and upstream ntDNA. The CH4 pathway appears to correspond to the CH1 solvent accessible path described in the literature [11,62,63]. An inspection of X-ray/cryo-EM structures reveals that CH3 is present in eukaryotic RNAPII in general. In bacterial RNAP, a channel comparable to RNAPII CH3A/B and leading directly to the i + 2 tDNA base is observed (PDB#4YLN, 4YLO, and 4YLP) [64]. Likewise, a pathway matching the CH3A/B location is also observed in archaeal RNAP (PDB#4V8S) [65].

**Figure 3:**
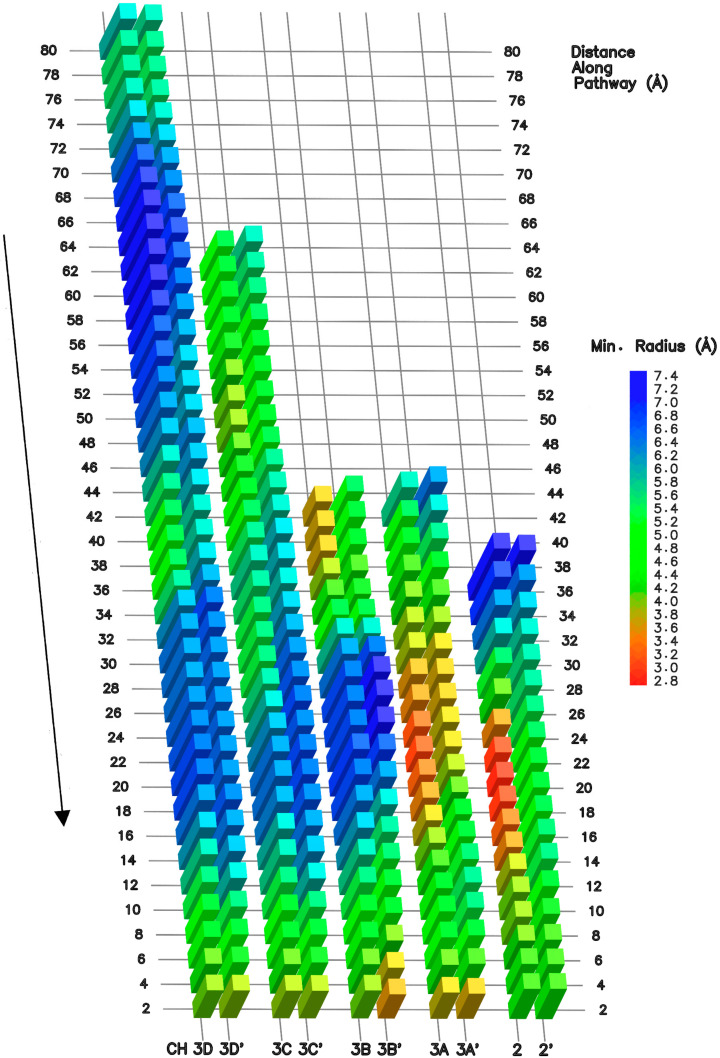
NTP pathways conformational fit. Right to left columns represent minimal radius along the pathway axis of CH2, CH3A/B/C/D, generated by the pathway-exploration algorithm. The displayed processed trajectory is the last 100 ns of simulation aMD_A1. The prime symbol indicates the conformational analysis spanning 100 ns in an alternative trajectory, i.e., CH2′ (aMD_A5), CH3A’ (aMD_B’1), CH3B’ (aMD_B4), CH3C’ (aMD_B4) and CH3D’ (aMD_B4). The arrow (left) indicates the direction from the exterior of to the enzyme to the buried i + 1 and i + 2 registers for the CH2 and CH3A/B/C/D pathways, respectively. The first displayed minimal radius value (top row) is from a distance of about 6 Å along the starting of the pathway (so as to discount the initial convergence of the exploration towards the optimal pathway center).

In order to characterize the suitability of the various channels for substrate accommodation, we developed a pathway-exploration algorithm. A series of points were iteratively calculated, defined as the furthest away from surrounding atoms, to draw a path describing the most central axis. Next, the minimal radius, defined as the distance to the closest atom, was extracted and used as a measure of the pathway width along its axis. Figure 3 summarizes the analysis outcome where two representative 100 ns trajectories were processed for each pathway. 

While CH3C and CH3D remain essentially unchanged and widely open in all simulations, CH2 and CH3A are the most circumstantial, and CH3B is modestly circumstantial. The CH2 opening varies along the corridor and in proximity to the active site, due to the shift in conformation of the central regions of the F loop [66,67], and trigger loop domains, referred to as “F loop central” (RPB1 790–800) and “TL central” (RPB1 1097–1119). The same mobile domains also open or close CH3A access. We identify four additional loops sporadically interfering with CH2, CH3A, and CH3B pathway accesses. First, a highly mobile amino acid chain (RPB1 606–627), which we term “CH2 loop”, displays conformational switches between the funnel and the corridor. Second, a loop (RPB1 757–779), positioned at the interface between the funnel helices [68] and the corridor, also gates access. The domain extends from the F loop N-Terminus (N-TER) to α21 of the funnel helix C-Terminus (C-TER). We term the element as “funnel loop”. Third, a loop located in the CH2 cleft region, comprises a central segment (RPB1 1314–1321), referred to as “cleft loop”, intermittently folding inside the CH3A entrance. Fourth, the C-TER region of the RPB9 subunit [69], is also highly mobile and CH3B initial access depends on the orientation of the loop. A portion of the RPB9 C-TER segment (97−109) periodically folds inside CH3B reaching the proximity of CH3P. The portion is referred to as the “RPB9 loop”. Altogether, the longitudinal dimensions of CH2 (Figure 3, approximate distance 26 to 12 Å from the pathway end) depend on the F loop central, TL central, CH2 loop, and funnel loop conformations. The dimensions of CH3A (Figure 3, distance 30 to 14 Å from the pathway end) depend on those of F loop central, TL central, and cleft loop. CH3B opening (Figure 3, distance 42 to 38 Å from the pathway end) relies on the RPB9 loop; CH3C and CH3D accommodation dimensions are reasonably constant.

### 3.3. NTP Contacting Macro-Regions

**Figure 4:**
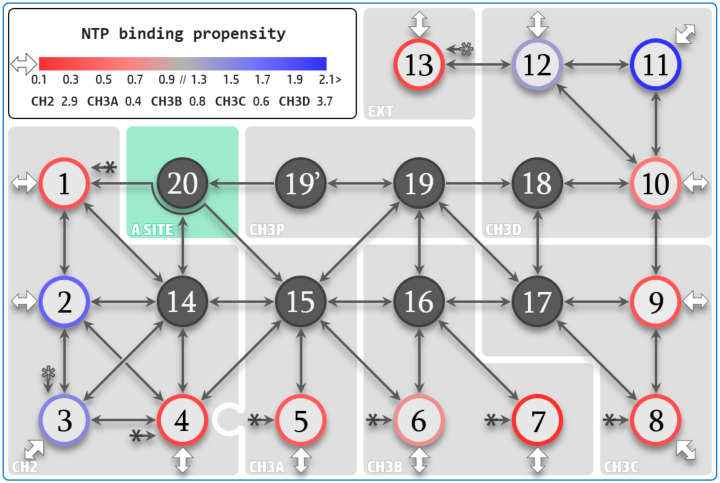
NTP diffusion map. Amino acids lying about the path of substrate diffusion are divided into twenty macro-regions (MR). Entry MR1 to 13 are shown as color-contoured discs, where the disc contour codes propensity to NTP binding. MR13, located externally (indicated by “EXT”) to solvent pathways, connects MR3 and MR12. Infiltration MR14 to 20, defined as not accessible directly from bulk solvent, are shown as grey discs. “A site” designates the active site region encompassed by MR20. MR19′ denotes necessary downstream NTP isomerization against the bridge helix before transfer to MR20. Solid and empty star symbols represent connections that are not fully drawn. White arrows indicate initial diffusion from the bulk solvent to the entry sites. The binding propensities are defined as the average number of bound NTPs during aggregate simulation time and are indicated for the entry macro-regions. Propensities are also listed for CH2, CH3A/B/C/D overall, and are defined as the cumulative average amount of NTPs bound to MR1/2/3/4/14 for CH2, MR5/15 for CH3A, MR6/7/16 for CH3B, MR8/9/17 for CH3C, and MR10/11/12/18 for CH3D. MR1/4/5/6/7/8 are interconnected through a common junction area (solid star symbol connection). The CH3A and CH2 pathways (MR5 and MR1/4) overlap about the funnel region (represented by the imbrication of the grey shaded areas).

Visualization of the overall substrate diffusion modes observed in the various simulations (aggregate simulation time of 6.5 μs) allows us to characterize a select number of residues that coordinate initial capture of NTP substrates at the entrance, and then follow them along the respective CH2/CH3A/B/C/D pathways. To facilitate such a description, we have divided the portions of the various pathways into twenty “macro-regions” (MR1 to MR20, Table S3). Macro-regions define a minimal patch on the NTP diffusion paths spaced to represent a modular portion connected to others to cover various portions of the diffusion pathways. MR1 to 13 are defined as the entry NTP binding regions that can be reached directly from solvent. MR1 to -12 belong to the NTP channels, while MR13 lies outside pathway entries, and connects MR3 and MR12. MR14 to 20 are defined as infiltration regions, accessible from the entry macro-regions solely. MR19 (CH3P) and MR20 (catalytic site) are the ultimate diffusion areas along CH3 and CH2, respectively. The NTP flow through the pathway macro-regions, together with entry NTP binding propensities defined as the average number of bound NTPs in aggregate simulation time are represented in Figure 4. Pathway NTP binding propensities are further discussed in the following sections.

### 3.4. NTP Loading Through CH2

**Figure 5:**
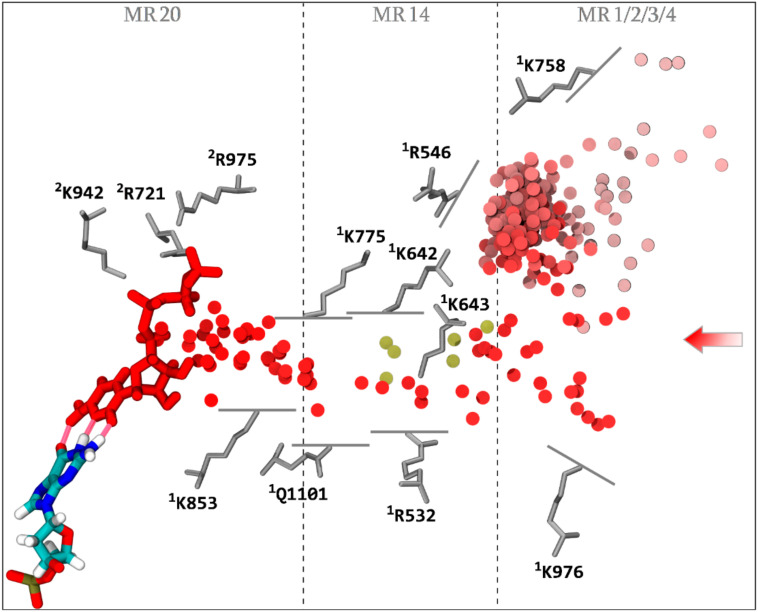
NTP loading through CH2. The successful diffusion trajectory across CH2 consists of the consecutive simulations aMD_A5, aMD_K2 (restart of aMD_A5), and aMD_L2 (restart of aMD_K2). The incoming NTP initiates diffusion into the funnel in aMD_A5 at 66.5 ns. The displayed trajectory is shown further along the funnel at the time point 102 ns. The center of mass of the NTP-MgB molecule, every 1 ns along the diffusion trajectory, is shown as a series of discs, color coded from light red to red, chronologically. The NTP substrate (MgB not shown) at the end of the displayed trajectory (red), and the i + 1 dNMP DNA register (colored by atomic type), are represented as sticks. The hydrogen bonds completing the CH2 diffusion process are represented as translucent red tubes. Key amino acids interacting with NTP-MgB are shown as grey sticks. Partial loadings along CH2 are represented as follows: most far-reaching alternative NTP diffusion from aMD_A4 (33.5 ns), aMD_E5 (119 ns), aMD_F4 (187 ns), aMD_L5 (93 and 118.5 ns), and aMD_L6 (101 ns) are aligned into the main trajectory and the center of mass of the NTPs are represented as gold discs. Entry macro-regions MR1 to 4, and infiltrations regions MR14 and MR20, are indicated.

Quantitative analysis of simulations aMD_A–M containing 15 mM of explicit NTPs in the solvent buffer allowed us to characterize four main areas coordinating NTPs at the entry of CH2 pathway. MR2 (contacting an average of 1.76 NTPs over the total simulation time and a maximum of seven NTPs at any given point in time) is of most significant relevance. MR3, comprising the foot domain, also displays a very strong contact propensity of 1.52 NTPs average (maximum of six NTPs). The overall CH2 binding propensity defined as the average number of substrates bound over the simulation time at MR1, 2, 3, 4, and 14 combined, amounts to 2.94 NTPs. The maximum number of substrates bound in CH2 at the same time amounts to nine. It follows that CH2 appears to offer very favorable NTP accommodation properties. Successful diffusion and binding to i + 1 (Figure 5, red color-coded discs) are sampled through the combined aMD_A5, K2, and L2 trajectories.

In addition, partial loadings in six alternative simulations were sampled (Figure 5, gold discs). Altogether, the following observations emerge. An energetic barrier seems to lie at the transition between the funnel (MR1/2/3/4) and the corridor (MR14). Although a significant number of NTPs are located in the funnel at any one time (on average 2.94), only seven transitions in the corridor (six partial loadings and one complete loading) were sampled. A second energetic barrier lies near the center of MR14, as only aMD_A5 captures a complete transition through MR14. Transition through the corridor involves rotation around **^1^**K642, **^1^**K643, and **^1^**R532 residues (with contribution of **^1^**K775); see Materials and Methods for the residue naming convention used in this paper. In simulation aMD_A5, the NTP binds in an inverted position through its phosphate group to **^1^**K775, **^1^**R532, and **^1^**K643, before undergoing rotation through the corridor. In the partial loading simulation aMD_F5, polyphosphate coordination is achieved through **^1^**K642 and **^1^**K643, preceding NTP rotation. In the additional partial loading trajectories, a similar mechanism was observed. Overall, incoming NTP triphosphate moieties anchor to two or more residues (**^1^**K642, **^1^**K643, **^1^**R532, and **^1^**K775), and then initiate rotation across the corridor. Once the rotation is complete and the NTP inserts through MR14, NTPs load via the remainder of the channel without encountering any significant energy barriers. Simulations aMD_K1 and K2 restarted from the rotated MR14 insertion state, sample diffusion to the active site region in 16 and 34.5 ns, respectively. Transition from MR14 to MR20 involves initial polyphosphate coordination through **^1^**K642 and **^1^**K775, followed by **^1^**K775 on its own, before detaching from **^1^**K775 and completing insertion into the catalytic site. Other amino acids belonging notably to the trigger loop and the bridge helix participate in the loading; detailed amino acid contributions along CH2 diffusion are listed in Table S3. The observation of the reverse diffusion trajectories (Figure S4) following a similar amino acid coordination map from the input path, supports that CH2 can exchange substrates in and out of the enzyme.

Following transition to MR20, simulations aMD_L1 and L2 sample bind to i + 1. Strikingly, no E site coordination [3,5,8,10] was observed; the incoming NTP bound directly to the i + 1 register without undergoing the preliminary inverted coordination to the metal binding sites (**^1^**D495, **^1^**D497, **^1^**D499, **^2^**E791, and **^2^**D792). Instead, we observed only a partial interaction with the metal site preceding binding to i + 1. In simulation aMD_L1, the NTP-bound MgB atom interacts weakly with **^2^**E791/**^2^**D792, oscillating about 3.5 to 6 Å from the residues, before binding directly to the DNA template register (Figure S5A). In simulation aMD_L2, MgB interacts electrostatically with **^2^**E791/**^1^**D495/**^1^**D497 from a distance of 3.5 to 5 Å, before binding to i + 1, afterwards actual metal site binding to **^1^**D497 occurs (Figure S5B). Successive i + 1 and metal site binding events are consistent with a PS (“preinsertion”) site configuration [70,71]. In addition, weak preliminary interaction with the metal site supports the possibility that metal site binding can precede i + 1 binding. Altogether, the observed loading trajectories support the concept that E site coordination is not an obligatory state of passage for the catalytic nucleotide and could occur before or after binding to i + 1.

Residues close to the catalytic site (**^1^**K775, **^2^**R721, **^2^**E791, **^2^**D792, **^2^**K942, and **^2^**R975, belonging to MR20) deserve further consideration. **^1^**K775, located at the central tip of the funnel loop, acts like a cantilever and displays substantial movement from the funnel all the way up to the catalytic site entrance and covers MR1, 14, and 20. Residues **^2^**K942 and **^2^**R975 belong to a subdomain of the hybrid binding region (RPB2 973–1073) located adjacent to the wall domain [72]. The latter domain comprises partially the “basic loop of hybrid binding region” [73] adjacent to the F loop and the bridge helix. We propose the term “F claw” for this domain. For the second subdomain of the hybrid binding region adjacent to the wall, spanning across the F claw, and adjacent to the link domain [74], we propose the term “sleeve” domain (RPB2 817–852). The sleeve domain comprises **^2^**E791 and **^2^**D792 that interact with the NTP MgB atom. **^2^**R721 of the link domain strongly interacts with the NTP phosphate group.

### 3.5. NTP Loading Through CH1

Access to the i + 2 register is granted by four subchannels, discussed previously, which connect to a cavity, CH3P, located inside the CH1 macro-channel surrounding the downstream DNA helix and upstream non-template DNA. First, we review the interconnection and state transition patterns of the subchannels, before exploring the diffusion and transient binding site propensities in more detail. On the one hand, hybrid steered/accelerated MD was employed to generate the path of optimal diffusion along the subchannels. First, a loading path across the CH3C subchannel to CH3P was produced employing a weak steering force (0.03 kcal/mol Å^−2^). The latter path was used as a reference to compare the potential convergence of the alternative channels along its trajectory (Figure S6). Loading of NTPs via the CH3C subchannel proceeded quickly across the pathway, indicating favorable access characteristics. A transitional barrier existed at the junction with CH3P and depended on a necessary rotation around **^1^**K1306 to insert inside the binding pocket. Then, the rotation was followed by binding to **^1^**K1133/**^1^**K1135 near the carboxy-terminal trigger base helix (TLc). On the other hand, steered MD through the CH3B subchannel was more direct as it merged directly into the CH3C path in front of CH3P. Similar to the CH3C path, the CH3B path offers propitious electrostatic and conformational properties, but is shorter and, more importantly, does not require rotation around **^1^**K1306 as the pathway is virtually directly aligned with CH3P. The CH3D path is more indirect than both CH3C and CH3B and transitions via CH3C. The pathway also appears to offer favorable NTP input properties because no significant energetic barrier is encountered from the entrance of CH3D to CH3C. The CH3A optimal steering path does not merge with CH3C until TLc coordination. The pathway connects CH3P directly and, similar to CH3B, does not require **^1^**K1306 rotation. Although offering a more direct pathway, the input propensity compares less favorably, as indicated by the slope of the diffusion curve (Figure S6). 

**Figure 6:**
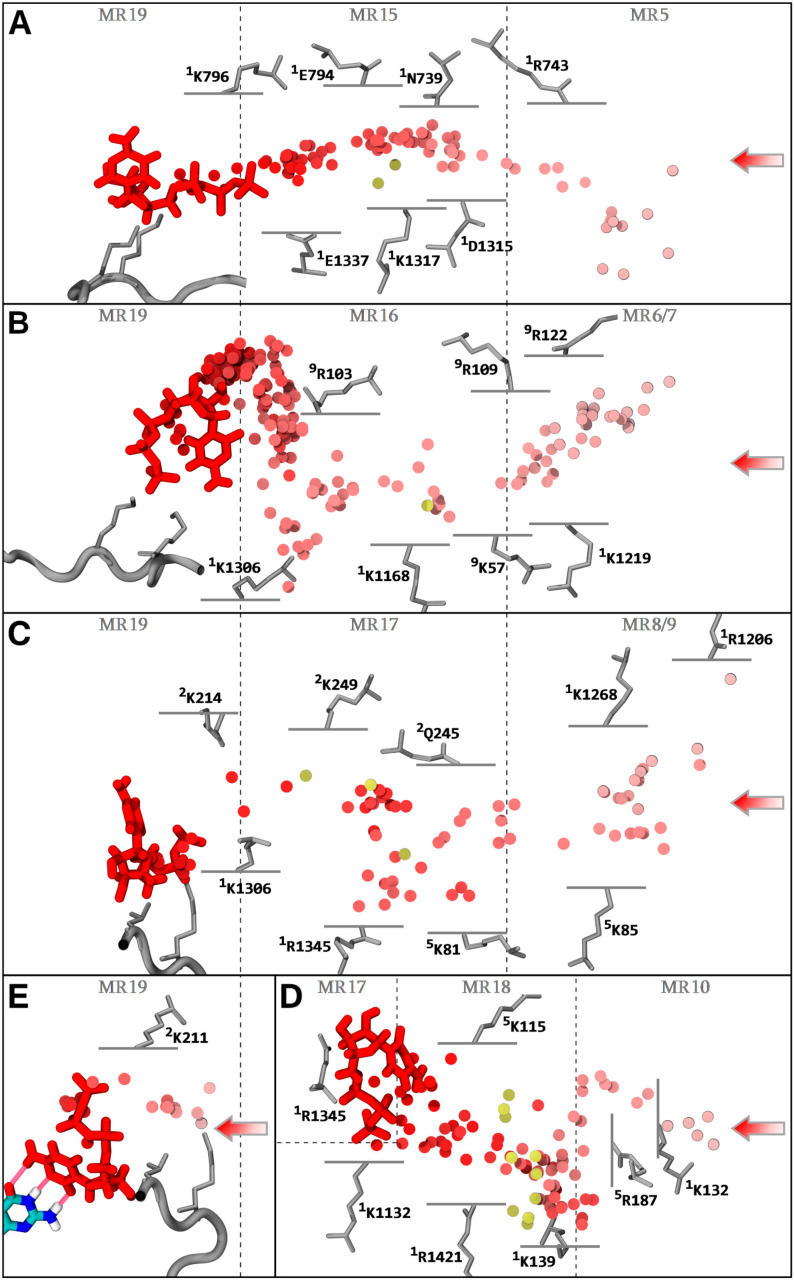
NTP loading through CH1. The center of mass of the NTP-MgB molecule along the diffusion trajectories is shown as a series of discs, color coded from light red to red, chronologically. The NTP substrate (MgB not shown) at the end (**A**–**E**, red) of the displayed trajectory, and the i + 1 dNMP DNA register ((**E**) colored by atomic type), are shown in stick representation. The hydrogen bonds completing the CH1 diffusion process (**E**) are represented as translucent red tubes. Key amino acids interacting with NTP-MgB are indicated. Trigger loop carboxy-terminal RPB1 1129–1137 is represented as a grey tube, with the **^1^**K1133/**^1^**K1135 NTP contacting residues indicated as sticks. Partial loadings along CH3A/B/C/D are represented. Most far-reaching NTP diffusions from alternative simulations are aligned into the main trajectory and the center of mass of the NTPs are represented as gold discs. Entry macro-regions MR5 to 10, and infiltrations regions MR15 to 19 are indicated. (**A**) Diffusion through CH3A from sMD/aMD simulation aMD_N1 (0−7.9 ns), with superimposed partial loadings from simulation aMD_A4 (52 and 190 ns); (**B**) Diffusion through CH3B from simulation aMD_I1 (0−69.8 ns), with a superimposed partial loading from aMD_A6 (258 ns); (**C**) Diffusion through CH3C, the trajectory is from simulation aMD_B2 (60.25−89.75 ns), followed up by simulation aMD_C1 (0−5 ns, restarted from aMD_B2), and the superimposed partial loadings are from aMD_A6 (338.5 ns), aMD_E4 (74.5 ns), and aMD_M3 (60 ns); (**D**) Diffusion through CH3D from simulation aMD_I2 (25.2−127.2 ns), with superimposed partial loadings from aMD_A6 (197 and 343 ns), A3 (121.5 ns), D8 (97 ns), D12 (51.5 ns), E2 (57.5 ns), E3 (73.5 ns), F2 (52 ns), H5 (141.8 ns), and J2 (26 ns); (**E**) Diffusion through CH3P. The trajectory is from simulation aMD_C1 (2−6.5 ns), followed up by simulation aMD_D12 (0−9 ns, restarted from aMD_C1).

Unbiased reaction-coordinate aMD simulations were used to capture spontaneous substrate diffusion deep inside the downstream bubble via three of the four subchannels. The interconnection patterns of the CH3 subchannels and the TLc as the target binding area, were corroborated. Indeed, examination of the diffusion trajectories from bulk solvent to the downstream bubble via CH3B (aMD_I1), CH3C (aMD_B2; aMD_C1), and CH3D (aMD_I2) supports the following conclusions (Figure 6B–D).

The CH3D most far-reaching sampled diffusion is characterized by delivery to **^1^**R1345, confirming the merging of CH3D with CH3C. The CH3C diffusion involves rotation around **^1^**K1306 and CH3B NTP loads to the TLc without **^1^**K1306 rotation coordination. No complete input diffusion and only limited reverse diffusion across CH3A were sampled in the aMD runs. This arises mainly from the fact that the starting structures for the simulation runs (except simulations aMD_B’) contained a trigger loop in the open conformation, which impedes access through the pathway.

Next, state transitions are to be further considered. Simulations aMD_B to D include an NTP molecule aligned in CH1, in addition to the bulk substrates. In simulations aMD_B, B’, and C, the transitive NTP is initially positioned before and after **^1^**K1306 rotation, aligned from sampled transition in aMD_A6 and B2. Simulations aMD_D1–D15 include a substrate initially aligned at a position after TLc coordination sampled in aMD_C1. This allowed us to further investigate the key CH3P junction region. The following state transitions were observed, some of which preceded the initial transitive position due to reverse diffusion followed by forward diffusion: Transition from **^1^**K1306 to TLc binding was observed in aMD_B1, B3, C1, D8, and D13 (Figure S7B–D,F,H) confirming the forward contact pathway. After TLc binding, simulations also revealed a CH3P insertion state involving **^2^**K211. Indeed, simulations aMD_B’1, D8, D12, and D13 (Figure S7E–H) and aMD_C1 (Figure S7B) captured, respectively, complete and intermediate insertion into CH3P through the anchoring of the NTP phosphate group to **^2^**K211 providing rotation of the NTP molecule. In simulation aMD_D12 (Figure 6E and Figure S7H), the insertion was completed by binding to i + 2, whereas partial binding was achieved in simulations aMD_B’1 and D8. The simulation aMD_B’1 (Figure S7G) contained the trigger loop in closed conformation, supporting that insertion was independent of the domain conformation. In simulations aMD_B1 and B3, incorrect insertion from TLc to CH3P was observed (Figure S7C,D), i.e., the triphosphate group shifted ahead into the cavity instead of the base moiety. Interestingly, no **^2^**K211 coordination occurs, which gives credence to the necessary involvement of the amino acid in the ultimate insertion transition. Finally, we observed a direct transition between CH3P and CH3D, i.e., skipping the CH3C pathway (Figure S7A), connecting the two areas via a reverse transition around **^1^**K1306 to TLc **^1^**K1132 residue extruding in CH3D. Other transitions in between adjacent subchannels are shown in Figure 4.

Considering the simulation data in general, the following key aspects of the diffusion properties emerge. For CH3A, substrate input is less advantageous for electrostatic reasons. Access is also highly dependent on the conformation of certain protein domains, especially the trigger loop. Nevertheless, two partial loadings were sampled in unbiased runs and diffusion across the channel was achieved with a very weak steering force (Figure 6A). This supports the conclusion that it is still a very suitable input channel. CH3D possesses the strongest entry macro-region transient binding sites. In particular, MR11 displays an outstanding binding average of 2.37 NTPs at any time. MR12 also displays important affinity for substrate binding (1.38 NTPs average), although direct binding from solvent can be lower, due to the input of neighboring MR11. MR11 and MR12 contribute largely for the average and maximum of NTPs lying in CH3D (3.73 and 10, respectively). The accumulation of substrates at the entrance of CH3D translated into ten partial loadings that were sampled (Figure 6D, gold discs). Subchannels CH3B and CH3C possess weaker transient binding sites, but, similar to CH3D, allow very fast substrate entry (Figure 6B,C and Figure S6). In particular, the weak propensity of CH3C to hold substrates at entrance could notably induce a higher probability of direct infiltration. CH3B and CH3C are also more tubular in shape, which could limit the diffusive degrees of freedom, thereby, improving forward channeling of incoming substrates. Finally, CH3A–D appears to allow the exchange of substrates in and out of the enzymatic complex, thereby the off-loading of mismatched NTPs, as characterized by the exit traces (Figure S8).

### 3.6. i + 2 NTP Isomerization, Downstream Scanning, and Transfer to the Active Site

**Figure 7:**
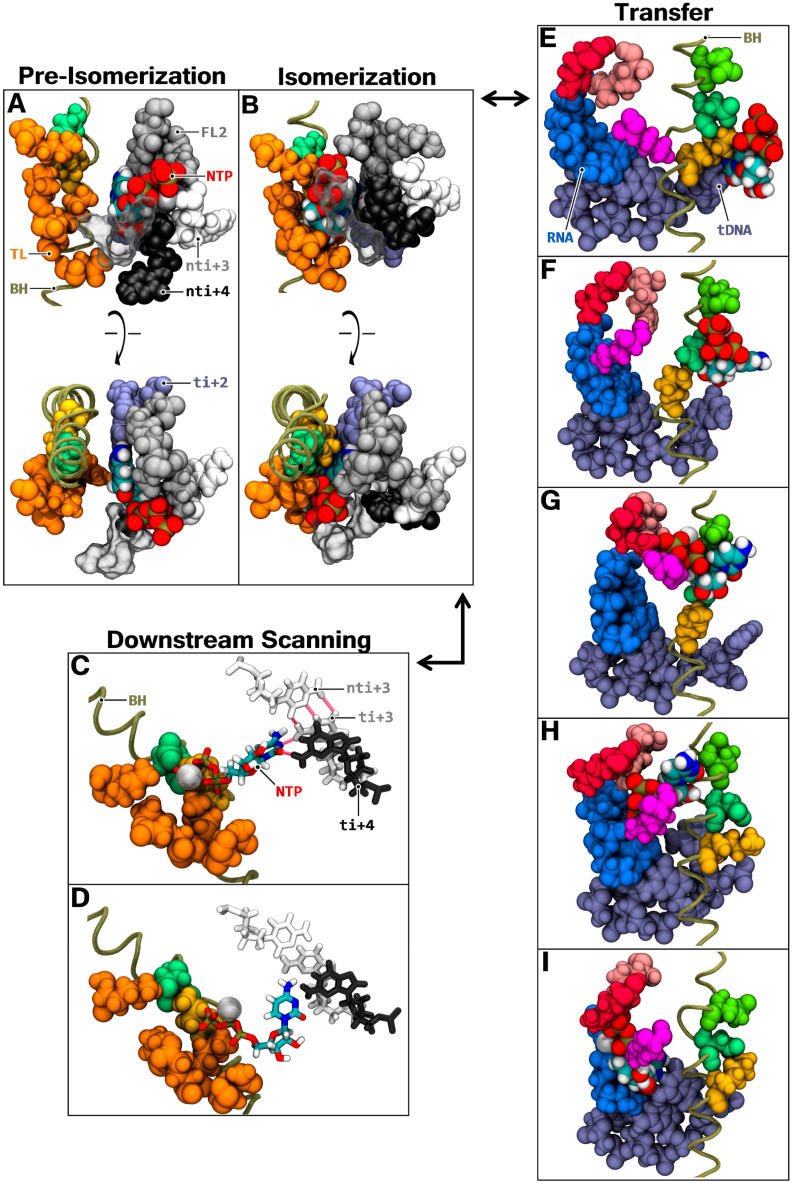
On-pathway fate of CH3P NTP. The bridge helix is represented as a tan green tube. The following amino/nucleic acids are shown in van der Waals spherical representation as follows: RNA i + 1 to i − 1 (medium blue); template DNA i + 2 to i − 1 (fade blue); bridge helix residues **^1^**E845 (fluo green), **^1^**D849 (green), **^1^**K853 (dark yellow); trigger loop residues **^1^**K1115, **^1^**P1122, **^1^**K1125, **^1^**E1126, **^1^**N1129 (orange); fork loop 2 residues **^2^**K494, **^2^**A496, **^2^**K497, **^2^**R499, **^2^**Q500 (grey); and key active site coordinating residues **^2^**R721 (pink), **^2^**R975 (red), **^1^**K775 (magenta). NTP bound magnesium ion, MgB, is shown as a silver sphere. Carboxy-terminal trigger base helix (TLc) **^1^**K1133/**^1^**K1135 are displayed as transparent surfaces. The NTP molecule (colored by atomic type), i + 3 (white) and i + 4 (black) registers, are shown in stick (**C,D**) and van der Waals representation (**A**,**B**,**E**–**I**). (**A**) Front and top view of the pre-isomerization coordination of an NTP in CH3P (aMD_E4, 5.5 ns); (**B**) Front and top view of the isomerization coordination (aMD_E4, 58.5 ns), following pre-isomerization; (**C**) First stage of the downstream scanning state. Partial bindings to i + 3/i + 4 occur (aMD_F3, 26.5 ns), with the hydrogen bonds highlighted in red; (**D**) Second stage of the downstream scanning state. The NTP molecule advances downstream and the phosphate group detaches from the isomerization contacts (aMD_F3, 46.5 ns); (**E**–**I**) The transfer of i + 2 NTP to the active site occurs on one side of the bridge helix, while the next template register translocates on the other side. The first state (**E**) is an isomerization frame (sampled in aMD_E4) before the bridge helix bending. The next states (**F**–**I**), are sampled from the isomerized NTP state, aligned in a protein structure with a bent bridge helix.

Following diffusion and binding to the i + 2 position, the fate of the NTP loaded via CH3P was investigated through a series of additional simulations. Six trajectories were run starting from the i + 2 bound position from aMD_D12, with harmonic restraints added to the hydrogen interaction between the i + 2 template register and the NTP, to characterize isomerization in CH1. Then, the i + 2 isomerized position was tested by running six simulations with the restraints relieved and six simulations with the i + 4 DNA pair restrained. In addition to off-pathway reverse diffusion and CH3P exit, four main productive on-pathway states emerged. The first state consisted of the consolidation of the immediate i + 2 binding position after **^2^**K211 rotation and insertion (Figure 7A).

We refer to the state as pre-isomerization. NTP-MgB is mainly coordinated by non-template i + 3 (binding MgB via phosphate oxygens) and i + 4 registers, fork loop 2, and TLc. Transition to the second state (“isomerization”), depends on the shift of the substrate towards the bridge helix and the more central part of the trigger loop (Figure 7B), generating strong interaction with **^1^**K853, **^1^**K1115, **^1^**K1125, and **^1^**E1126 (Table S4). Long-lasting pre-isomerization configurations were observed in simulations restarted from the i + 2 binding position (aMD_E2 to E6). Transition to the isomerization state was sampled in aMD_E3 and E4. The isomerization configuration was characterized from the latter transitions and from restarts of the sampled transition (aMD_F2, F4, and F5). 

The role of nti + 4 deserves further discussion. In aMD_A to M, an intriguing stochastic flipping of the nti + 4 DNA base in and out of CH3P occurs, suggesting the presence of a specific molecular mechanism. To investigate the role of the non-template register, isomerization position simulations run with a freely mobile base were compared with simulations where the i + 4 DNA bond was constrained to prevent flipping of the nucleotide. Prolonged isomerization was only observed when nti + 4 was freely mobile. In simulations aMD_F’1 to 4, and aMD_F’6, the NTP promptly lost binding to the i + 2 register and lost isomerization, whereas in aMD_F2, F4, and F5, prolonged isomerization was observed. Furthermore, aMD_F2, F4, F5, and F6 displayed long-lasting i + 2 binding. Therefore, we conclude that nti + 4 contributes strongly to substrate confinement. 

The third possible on-pathway outcome involves the shift of the NTP about the isomerization configuration to scan i + 3/i + 4 positions, where the substrate NTPs can attempt binding. We refer to the state as “downstream scanning”. Transition from isomerization to downstream scanning was sampled six times (aMD_F1, F3, F’3, F’4, F’5, and F’6). The shift to i + 3/4 was carried out in two stages. First, the NTP rotated directly towards downstream DNA and bound partially to i + 3/+ 4 (Figure 7C), with an affinity with the right-handed lining atoms, which were more directly available in CH3P (H21/H22 s nitrogen hydrogens in case of guanine). During the rotation, the triphosphate group remained anchored to the trigger loop/bridge helix, in a disposition comparable to the isomerization state. At the second stage, the phosphate group detached from its initial contact point adjacent to the bridge helix, allowing the NTP to shift forward towards downstream registers (Figure 7D). In the process, contact with **^1^**K1115 was lost, and the nucleobase advanced in close proximity to the switch 1 domain. Regarding the availability of the i + 3 and i + 4 DNA template bases for NTP binding, the following is noted. While the i + 4 position is largely melted, i + 3 remains mainly associated. However, quantitation of DNA melting indicates that the presence of an NTP in CH3P stimulates i + 3 melting (Table S5). The latter position evolves from 9.26 to 29.34% and 5.57 to 20.47% melting and partial melting time fractions, respectively. Cumulative partial and full melting of i + 3, therefore, amounts to ≈50% when an NTP is in the downstream scanning state, which appears to be sufficient to allow stable binding at that position. Interaction of an NTP with an i + 3 dNMP, whilst i + 3 DNA pair is associated, was observed (Figure 7C). This suggests a mechanism of hydrogen contacts trading between the CH3P loaded NTP and the downstream i + 3 DNA pair. In other words, the melting of the i + 3 position appears to occur with sufficient frequency to allow stable binding at the register, but an incoming NTP can also destabilize the paired DNA position to induce the availability of the template dNMP for binding.

The last on-pathway state consists of the transfer of the i + 2 isomerized NTP to the catalytic site. A transfer intermediate was detected in aMD_F6 and F’6, where the triphosphate portion shifted in the direction of bridge helix N-TER, and where MgB advanced near **^1^**E794/**^1^**E845/**^1^**D849. To further investigate the transfer process, the NTP isomerization configuration was aligned in a structure containing a bent bridge helix. Simulations aMD_G1, H2, I3, and J4 (aMD_H2, I3, and J4 were restarted from aMD_G1, H2, and I3, respectively) sampled a complete forward translocation step, accompanied with the transfer of the i + 2 NTP to the active site. The downstream template register shifted from the i + 2 to the i + 1 position, while the NTP molecule was shuttled to the other side of the bridge helix between the trigger loop and the F loop, and reassociated with the translocated DNA register (Figure 7E–I). During the process, up to 41 residues belonging to fork loop 2, trigger loop, bridge helix, F loop, F claw, link, and sleeve contact the NTP (Figure S9). A very important conclusion that emerges from this simulation data is the confirmation that i + 2 NTP can be shuttled to the active site, concomitantly to translocation, to become the next polymerization substrate (corroborating *E. coli* results) [19]. The i + 2 NTP appears to not only function as an allosteric effector but can also assume the role of substrate.

### 3.7. Synergistic Coupling of Downstream Binding and Catalytic Isomerization

**Figure 8:**
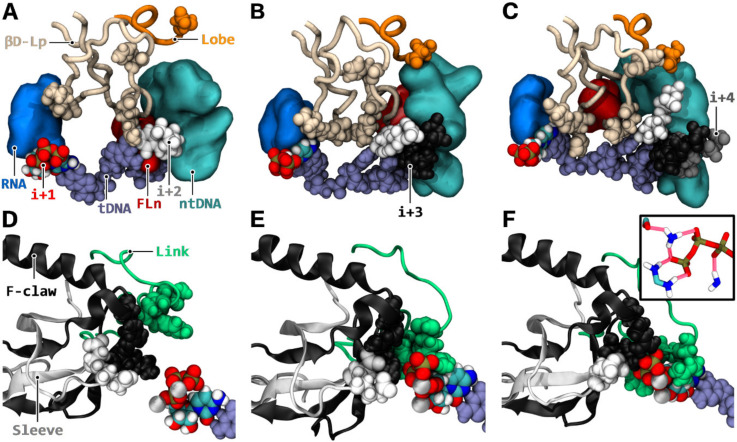
Mechanistic reorganizations associated with downstream binding. Left (**A,D**), middle (**B,E**), and right (**C,F**) columns: respective simulated configuration after 100 ns with i + 2 (aMD_M1); i + 2/+ 3 (aMD_M2); and i + 2/+ 3/+ 4 (aMD_M3) NTPs paired to template DNA. Template DNA (fade blue van der Waals spheres) is represented from i + 4 to i + 1 (**A**–**C**) or sole i + 1 (**D**–**F**) positions. Active site i + 1 NTP is colored by atomic type. i + 2, i + 3, and i + 4 NTPs (**A**–**C**) are in white, black, and grey van der Waals spherical representation, respectively. (**A**–**C**) Coupling of downstream binding to βD loop reorganization. βD loop, RPB2 483–527 (beige tube), lies in between template DNA, non-template DNA (cyan surface, represented from i−1 to i + 6), and RNA (medium blue surface, represented from i to i−2). Fork loop 2 N-TER segment RPB2 491–494 (red surface) is represented. C-TER portion of the lobe domain RPB2 384–392 is shown as an orange tube. Lobe βD loop interacting residue **^2^**D384 is shown in van der Waals representation. βD loop junction residues are shown as follows: **^2^**H502 (top), **^2^**R499 (right), and **^2^**E516/**^2^**G517/**^2^**H518 (left); (**D**–**F**) The distortion of template DNA is propagated to i + 1 bound NTP (MgA and MgB ions are represented in silver), which flips towards link RPB2 713–737 (green), F claw RPB2 928–992 (black), and sleeve RPB2 772–807 (white) domains. Highlighted in van der Waals representation are the following NTP surrounding residues: link (**^2^**R721, **^2^**Y724, and **^2^**T723), F claw (**^2^**R975 and **^2^**K942), sleeve (**^2^**E791 and **^2^**D792); (**F inset**) The NTP triphosphate group is tightly coordinated by the sleeve, F claw, and link domains. The hydrogen bonds between the NTP and F claw **^2^**K942 (atoms HZ2, HZ1, HZ3, and NZ, shown top left); F claw **^2^**R975 (atoms CZ, NH2, HH22, NH1, HH11, HH12, and HH21, shown bottom left); and link **^2^**R721 (atoms HH12, NH1, and HH11, shown bottom right) are represented as red translucent tubes. Sleeve **^2^**D792 (atoms CG, OD1, and OD2, shown top left corner) hydrogen bond with **^2^**R975 is represented.

According to published data, the multiple occupancy of downstream binding sites at the i + 2/+ 3/+ 4 positions appears to propagate an intriguing long-range conformational change acting on the active site [17,18]. In order to investigate a possible synergistic mechanism, we ran three aMD simulations involving a paired i + 1 register together with paired i + 2 to i + 4 registers. Compared to the simulation trajectories with unpaired downstream sites, or only a paired i + 2 position, the simulation data exhibit spectacular long-range conformational changes when i + 3/+ 4 are paired in addition to i + 2. These involve notably a subset of the RPB2 fork region comprising fork loop 2 and fork loop 3, which is also referred to as β/RPB2 D loop, D loop II, D fork, and D domain [23,75,76,77,78]. The subset RPB2 483–527 is referred to as the “βD loop”. The observed mechanistic modifications are as follows: When the sole i + 2 NTP-dNMP pair is present, amino acid segment RPB2 491–494 lying at the N-TER of fork loop 2 (“FL2n”), remains in CH3P against the i + 2 NTP-dNMP pair (Figure 8A). 

With the i + 3 bound NTP present in addition to i + 2, induced spatial saturation displaces FL2n from CH3P (Figure 8B). Then, FL2n stacks against the template i + 4 position outside CH3P. The paired i + 4 position removes the FL2n stack against i + 4, by repositioning the template register inside CH3P, and as a result FL2n entirely flips away and around the βD loop (Figure 8C). Modest and robust repositioning of FL2n with i + 3 and i + 3/i + 4 pairs, respectively, trigger a large-scale nonlinear shift of βD loop (Figure 8A–C). Then, the domain reorganizes in the catalytic cavity, thereby, strongly improving the confinement of the i + 1 register region. Another long-range and synergistic conformational change occurring concurrently to βD loop reorganization is propagated via the template DNA strand. Downstream NTP pairings re-orientate the template registers inside CH3P, while aggregating through stacking interactions, and the βD loop stacks against the i + 2 pair at the downstream/upstream junction. As a result, a switch in conformation of template DNA section i + 2 to i + 4 is generated, allowing a structural distortion to propagate to the i + 1 position upstream. This in turn, flips i + 1 NTP towards the F claw, sleeve, and link domains (Figure 8D–F), thus, effectively enhancing further the isomerization already taking place via the refolding of the βD loop. Otherwise, non-template DNA conformational changes can also occur, but this affects only the case where the i + 4 position is bound in addition to i + 3. Repelling of an ntDNA segment away from the i + 4 dNMP-NTP pair, in conjunction to βD loop refolding, triggers, indeed, a rearrangement of a large portion of ntDNA around βD loop (Figure 8C). Then, the C-TER segment of the lobe domain (RPB2 384–392) inserts robustly between the repelled ntDNA strand and βD loop (Figure 8C), further locking the refolded states of both βD loop and ntDNA. Finally, the NTP bound downstream registers act as an anchor to a trigger loop segment (1131−1136), which could indirectly stimulate trigger loop tip rotation (Figure S10).

## 4. Discussion

For the past twenty years, the NTP entry into the catalytic center of cellular RNAPs has been described through two conflicting models, i.e., direct and indirect access via CH2 and CH1, respectively. The MD simulations described here elucidate the structural bases of the CH1 loading process in human RNAPII and support that substrate binding to the i + 2 up to i + 4 registers, together with the subsequent shuttling of the pre-bound NTPs to the active site, are feasible and realistic processes. They further support a model in which the CH1 and CH2 mechanistic pathways are not necessarily contradictory, but rather describe distinct elongation regimes [19]. In this model, one mode could indeed apply over the other depending on the transcriptional conditions. The TFIIF reconfiguration of the downstream bubble appears to enable CH1 loading (Figure 2 and Figure S2). Corroborating our results, Förster resonance energy transfer (FRET) data are consistent with the hypothesis that TFIIF actively remodels DNA inside RNAPII during transcription elongation [79]. Interestingly, when CH1 is enabled, CH2 loading could be repressed because at each nucleotide addition round, the next NTP (i + 2 NTP) is selected downstream during the incorporation of i + 1 NTP, is transferred to the active site together with translocation (Figure 7), and then sterically blocks access via CH2 while completing the nucleotide selection process. Therefore, NTPs load primarily via CH1 in the presence of TFIIF and load primarily via CH2 in its absence. The following experimental data strongly corroborate the existence of a conditional (and more efficient) CH1 mechanism. Significant elongation rate stimulation at very low NTP concentrations and kinetic switch to the rapid phase regime, in human and yeast RNAPII when supplemented with TFIIF (as well as in *E. coli* RNAP when comprising fork loop 2 required for i + 2 binding), have been reported in quench-flow kinetic and single-molecule optical trapping experiments [19,20,21,22,80]. 

Considering the correlation between the NTP loading mechanisms and the nucleotide addition cycle in more detail, the following hypothesis emerges: The CH1 mode of substrate loading appears to significantly support additional modes of the general transcription process by RNA polymerases. The presence of two mechanisms could allow distinct elongation regimes to be expressed, thereby, allowing the tuning of transcription. Important implications include the NTP selection time window, coupling, transition, fidelity, and pause escape as follows:

- **NTP selection time window**. When NTPs were loaded via CH1, the enzyme performed two tasks at the same time. It selected the next nucleotide(s), up to the i + 4 register (Figure 7C,D), while incorporating the current NTP. This could be especially advantageous kinetically when NTP concentrations are low or stoichiometrically imbalanced.
- **Coupling.** In the context of several successful downstream NTP bindings, the induced refolding of the βD loop and stretching of the template DNA strongly isomerized the active site (Figure 8), thereby, potentially accelerating the incorporation of i + 1 NTP. As such, a coupling between catalysis and subsequent substrate availability could occur.
- **Transition.** Transition between nucleotide addition rounds is likely to be stimulated in four ways. First, during induced isomerization, with NTPs bound to i + 2/i + 3/i + 4, an enhanced strain was applied onto the i + 1 NTP polyphosphate group (Figure 8E,F), which could result in improved PPi-MgB release. Pyrophosphate exit may not be rate limiting [81], therefore, only a modest stimulation would be expected. Second, the downstream NTP bindings stretched template DNA and shifted non-template DNA away (Figure 8A–C), which could bias translocation to the forward state. Third, the modification of the trigger loop dynamics via the NTP anchoring of TLc (Figure S10) could accelerate trigger loop closing. Fourth, because i + 2 NTP was, or was likely to be transferred to the active site concomitantly to translocation (Figure 7E–I and Figure S9), there is an increased probability that the ratchet was immediately incremented after translocation. However, employing the CH2 mechanism, the right NTP must first successfully be selected after vacation of the active site before translocation oscillation would be adjourned.
- **Fidelity.** When the next NTP binds to i + 2, first, it was isomerized in CH3P (Figure 7B), then detached from template DNA (Figure 7F), and transferred to the catalytic site (Figure 7I) before undergoing final catalytic confinement. Therefore, there is a double isomerization process at play, utilizing the energy of base pairs twice, and delayed in time. In addition, the re-enforced catalytic confinement occurring when multiple downstream positions were occupied (Figure 8) could reduce the probability of i + 1 misincorporation by reducing the possibility of altered Watson–Crick geometry stabilization in the active site.
- **Pause escape.** The same mechanism responsible for re-enforced catalytic isomerization on the i + 1 NTP that improves fidelity could also be involved in facilitating pause escape by stimulating realignment of the RNA 3′ end.

## 5. Conclusions

In summary, results from the MD investigation of human RNAPII presented here interpreted in the light of pre-existing literature data, are consistent with the hypothesis that human and yeast TFIIF-bound RNAPII, together with *E. coli* RNAP, can accommodate substrate loading via CH1. A subsidiary hypothesis is that when the CH1 elongation regime is disabled, notably in TFIIF-free RNAPII, delivery through CH2 would operate by default. Alternative means of substrate loading could apply to cellular RNAPs in general and could impact in a strongly dissimilar fashion substrate selection kinetics, coupling, transition, fidelity, and pause escape, thereby affecting the overall transcription landscape both qualitatively as well as quantitatively. The main amino acids coordinating the NTP loading processes presented in this article are conserved among eukaryotes and archaea (Tables S6 and S7), advocating for a universal mechanism among the latter domains of life. Future investigation could focus on the derivation of the on- and off-pathway NTP diffusion rates along the binding macro-regions and their correlation to experimental elongation rates; downstream bubble remodeling modulation in bacterial and archaeal RNAPs [82,83,84], as well as in RNAPI and III [85,86,87]; regulation of TFIIF association [88,89,90,91]; and the utilization of the various NTP loading modes for different stages of the transcription cycle (initiation, pause, arrest, termination). 

## Figures and Tables

**Table 1:** Simulation tree.

Simulation ID	Protein Structure	Transitive NTP Restart ^1^	Observations
aMD_A1–6	PDB#5IY9		Initial structure.
aMD_B1–4	aMD_A1 168.5 ns	aMD_A6 233 ns	CH1 loading state sampled in aMD_A6 is aligned into aMD_A1 displaying a fully stabilized RNAPII-TFIIF complex.
aMD_B’1–2	aMD_A1 168.5 ns	aMD_A6 233 ns	Same as above but the trigger loop is in closed conformation (templated from PDB#2E2H).
aMD_C1–4	aMD_B2 89.75 ns		aMD_B2 comprises an NTP loaded through CH1, RNAPII-TFIIF complex is stable.
aMD_D1–15	aMD_A1 181 ns (D1 to D5), aMD_B2 72.5 ns (D6 to D10) and aMD_B3 108 ns (D11 to D15)	aMD_C1 6.5 ns	CH1 preinsertion state sampled in aMD_C1 is aligned into structures with three different open trigger loop conformations.
aMD_E1–6	aMD_D12 9 ns		An NTP is bound to the i + 2 register. The nucleic pair is kept attached with restraints.
aMD_F1–6	aMD_E4 56 ns		The i + 2 bound NTP is isomerized.
aMD_F’1–6	aMD_E4 56 ns		Same as above, but the i + 4 DNA pair is restrained.
aMD_G1–2	aMD_A1 87 ns	aMD_E4 56 ns	The i + 2 isomerized NTP state is aligned into a structure displaying a bent bridge helix.
aMD_H1–5	aMD_G1 11.4 ns		NTP forward transfer intermediate towards the active site.
aMD_I1–3	aMD_H2 55.8 ns		Forward transfer intermediate.
aMD_J1–7	aMD_I3 21.4 ns		Forward transfer intermediate.
aMD_K1–2	aMD_A1 110 ns	aMD_A5 359 ns	CH2 loading state sampled in aMD_A5 is aligned into aMD_A1 displaying a stabilized RNAPII-TFIIF complex.
aMD_L1–6	aMD_K2 34.5 ns		NTP loaded through CH2 corridor.
aMD_M1–3	aMD_D12 9 ns (M1), aMD_D7 12 ns (M2, M3)		aMD_M1–3 inputs are completed with i + 2, i + 2/+ 3 and i + 2/+ 3/+ 4 NTP bound registers.
aMD_N1–4	aMD_B’1 12 ns (N1), aMD_B4 117 ns (N2), aMD_A1 107 ns (N3), aMD_A1 182 ns (N4)		Hybrid accelerated/steered MD method to investigate the loading through the different CH1 entry paths.

The protein structure column specifies the provenance of the RNAPII-TFIIF complex input coordinates. **^1^** The field specifies the optional addition of an NTP (diffusion transition state) into the protein structure taken from an alternative simulation.

## Supplementary Materials

The following are available online at https://www.mdpi.com/2218-273X/10/9/1289/s1, Figure S1: Structural alignment of the 10 subunit preinitiation and elongation RNAPII complexes, Figure S2: TFIIF reconfiguration of the downstream bubble, Figure S3: CH1 stabilization into the close conformation, Figure S4: CH2 exit traces, Figure S5: Active site loading transitions, Figure S6: Comparative biased reaction-coordinate diffusion through CH1 subpathways, Figure S7: CH3P state transitions, Figure S8: CH1 exit traces, Figure S9: i + 2 NTP transfer and reassociation with the translocated i + 2 template register, Figure S10: Coupling of downstream binding with trigger loop closing, Table S1: Amino acid contribution to the locking of the i + 2 register, Table S2: Amino acid sequence of the pathway interlining residues, Table S3: Amino acid sequence of the NTP binding macro-regions, Table S4: Amino/nucleic acid contribution to the pre-isomerization and isomerization states, Table S5: Melting state of the downstream registers, Table S6: RPB1 subunit conservation, Table S7: RPB2 subunit conservation.

## Appendix A

Supporting information protocol. Potential energy minimization is carried out with Amber16 pmemd.cuda through a first stage (“min1”) consisting in 1000 and 4000 steps of steepest descent and conjugate gradient algorithms respectively, with 500 kcal/mol harmonic positional restraints on protein and nucleic atoms; and a second stage (“min 2”) consisting in the computation of 2500 steps of each algorithm with restraints on the four DNA extremity residues only. The metabolites, including a 15 Å buffer water box and the CTP substrates, are randomly placed in the simulation box with the Amber16 AddToBox module. In some simulations, indicated below, minimization is performed on an expurgated system (lower computational cost to optimize the minimization algorithms). No r^−4^ term is added to divalent and monovalent ions and only K^+^ metabolites are added to balance the charge. K^+^ counter-ions are inserted in the charge grid in this case with xLeap instead of AddToBox to optimize neutralization. In these expurgated preliminary systems, water molecules are also added with xLeap, and with a 15 Å buffer except in simulations aMD_A where an 18 Å buffer is used in a preparation step to stabilize the intrinsically more unstable initial structure. The latter systems are then stabilized by several rounds of min1 and min2 runs with Amber16 pmemd.cuda. When minimization rounds are performed on a system prepared that way, they will be referred below as “pre-min” rounds. Heating procedure (hereafter referred to as “heat”) is carried out with 10 kcal/mol restraints on the protein and nucleic atoms, for 20 ps. Velocity equilibration (“eq-vel”) is run as NVT (constant moles, volume and temperature) for 20 ps in simulations aMD_B, B’, G, N, for 1 ns in aMD_D, E, K, M and for 20 ns in aMD_A. No eq-vel is computed for aMD_C, D, F, F’, G, H, I, J, L. Box equilibration (“eq-box”) is run as NPT (constant moles, pressure and temperature) with a 1 bar Monte Carlo barostat. Heat, eq-box and eq-vel are run with a Langevin integrator. Accelerated Molecular Dynamics (“aMD”) runs are performed with a dual boost integrator and an Andersen thermostat. Heat, eq-box, eq-vel and aMD runs are computed with OpenMM 7.0, use a temperature of 300 K, a timestep of 2 fs, a thermal collision frequency of 1.0 ps^−1^, a non-bonded Particle Mesh Ewald method with a cutoff of 8 Å, constrained hydrogen bonds, rigid water molecules, and harmonic bond restraints (ranging from 10 to 50 kcal/mol) added to the hydrogen bond forming atoms of the four DNA extremity residues. In simulations aMD_K to L with neither the RNA 3′end nor a CTP-MgB molecule in the active site, the RNA length was 17 nucleotides, there were a total number of atoms of 659,340, 146,582 water molecules, 8 protein-bound Zn^2+^ atoms, 2 protein-bound Mg^2+^ ions in addition to MgA, 400 K^+^, 40 Na^+^, 228 Cl^−^, and 40 Mg^2+^ bound CTPs in the simulation box. In simulations aMD_E to J comprising the RNA 3′end in the active site, the RNA length was 18 nucleotides, the total number of atoms was 659,370 and there were 227 Cl^−^ ions; the other molecular species counts were identical. In the remaining simulations, where the active site was occupied by a CTP-MgB substrate, the RNA length was 17 nucleotides, the total number of atoms was 659,380 and there were 226 Cl^−^ atoms. The number of protein residues used for the calculation of the dihedral acceleration parameters is defined as the total number minus 1 of the dihedral containing residues, which includes the CTP substrates: 4296 + 40 = 4336. The acceleration parameters alpha dihedral, dihedral boost potential, alpha total and total boost potential are 16,328, 322,268, 551,769 and −7,389,886 kJ/mol respectively. The 12-6-4 potential, a potential taking partially into account polarizability and describing in a more realistic fashion the strongly charged divalent ions, was assigned to the magnesium atoms. Alternatively, the 12-6 van der Waals parameters are used between non-template i + 3 OP1, OP2 atoms and Mg^2+^ in simulations aMD_E, F and F’, between aspartate **^1^**1295:OD1/OD2 atoms and Mg^2+^ in aMD_B and between all aspartate OD1/OD2, glutamate OE1/OE2 atoms and Mg^2+^ ions in aMD_B’, C, D, H, I, K. The latter parametrization choice is motivated by the observation that the van der Waals radius of the magnesium ion can collapse inside the van der Waals radius of oxygen atoms, due to the intrinsic limitations of non-fully polarizable forcefields. Hence, in the simulation runs where Mg^2+^ atoms were too close to oxygen atoms (interatomic distance below 2 Å) at the starting coordinates (t = 0 ns), the r^−4^ term was not added. The simulation durations in nanoseconds are: aMD_A1 (251.5), A2 (250), A3 (252), A4 (251), A5 (368.5), A6 (365), B1 (75.75), B2 (122), B3 (138.5), B4 (123.5), B’1 (101), B’2 (100), C1 (31), C2 (27.5), C3 (28.5), C4 (27), D1 (13), D2 (13), D3 (18), D4 (28.5), D5 (28), D6 (16), D7 (15.5), D8 (107.5), D9 (31.5), D10 (31.5), D11 (16), D12 (71), D13 (19.5), D14 (38.5), D15 (27), E1 (67), E2 (57.5), E3 (75.5), E4 (77), E5 (124.5), E6 (124), F1 (102.5), F2 (74), F3 (52.5), F4 (189), F5 (162), F6 (74.5), F’1 (54.5), F’2 (54.5), F’3 (54.5), F’4 (55.5), F’5 (58.5), F’6 (55.5), G1 (22.2), G2 (49.8), H1 (53.5), H2 (101.8), H3 (40.4), H4 (95.2), H5 (169.6), I1 (89), I2 (156.6), I3 (75), J1(40.8), J2 (40.2), J3 (12.2), J4 (68.8), J5 (12.4), J6 (14.2), J7 (94.2), K1 (56), K2 (93.5), L1 (59), L2 (67), L3 (61.5), L4 (62), L5 (118.5), L6 (101), M1 (100), M2 (100), M3 (100), N1 (7.9), N2 (2.1), N3 (6.3), N4 (2.9). **Preliminary steps:** The initial system is PDB#5IY9. The preinitiation Complex is stripped to a 10-subunit RNAPII-TFIIF complex by removing RPB4/7, TFIIA, TFIIB, TFIIE, TFIIH, XPB/XPD helicases and the TATA-binding protein. The DNA helix is stripped to positions i − 30 to i + 20 so as to exclude the segments significantly external to the enzymatic complex. The RNA strand is prolonged to position i − 16. The missing loops, not resolved in the initial X-ray structure, are inserted with Yasara Structure. A CTP-MgB molecule is inserted in the active site. TFIIF RAP74 segment 181-227, obtained via homology modelling, is aligned to TFIIF subunit RAP74 amino acid position 180. Only charged ends are added at the N-TER and C-TER portions of the respective subunits (no neutral caps). One pre-min round is computed without DNA restraints and with an 18 Å buffer water box. Heat is performed for 20 ps with a non-bonded cutoff of 10 Å. Then eq-vel (100 ps), eq-box (100 ps), eq-vel (20 ns) and single dihedral boost aMD (15 ns) are run with mass constraints on DNA extremities and residues lying at C-TER of RPB1 and N-TER of RPB6. The final box dimensions are x = 187.793, y = 220.507, z = 179.572 Å, dihedral acceleration parameters are alphaD = 16,177, EthreshD = 319,645 kJ/mol and the total number of atoms is 838,730. The structure is then minimized through twenty rounds of pre-min. The preliminary system, which we will term “5IY9-prel”, is complete. Then water molecules are stripped off, neutral caps are added at C-TER of RPB1, N-TER of RPB6, C-TER of TFIIF subunit RAP74, 10 mM Mg^2+^ (53 elements), 15 mM Na^+^ (40 elements), 150 mM K^+^ (400 elements) and Cl^−^ (408 elements) concentration to ensure charge neutrality, are added, and water molecules, respecting a total 15 Å buffer distance to protein/nucleic atoms, are added. Min1, min2 with restraints on C-TER of RPB1 and N-TER of RPB6, 20 ps heat with a non-bonded cutoff of 10 Å, 100 ps eq-vel, 20 ns eq-box and 20 ns eq-vel, are run without constraints. Unbound Mg^2+^ atoms are stripped off, resulting in only two Mg^2+^ ions (in addition to active site MgA/MgB) that are kept protein-bound. A calculated amount of Cl^−^ atoms is removed to balance the charge, water is stripped off, 15 mM CTPs are inserted, and then water (146,582 molecules) is added back again. The final total number of atoms is 659380. Then min1, min2 with positional restraints on C-TER of RPB1 and N-TER of RPB6, 20 ps heat with non-bonded cutoff of 10 Å, 100 ps eq-vel, 20 ns eq-box and 20 ns eq-vel are run again. **aMD_A1-6:** After the aforementioned preparation steps, the first long timescale production aMD runs are computed, allowing the system to explore the conformational landscape and NTPs to diffuse about the enzymatic complex. **aMD_B1-4:** The initial system is taken from an output frame of simulations aMD_A displaying a stabilized TFIIF-protein complex and the i + 2 register locked in CH3P. An additional NTP (which we will refer as “transitive NTP”) is aligned into the structure, which coordinates are taken from another output of simulation aMD_A displaying a CTP rotated around **^1^**K1306. **aMD_B’1-2:** The initial system is an output of aMD_A supplemented with the transitive NTP rotated around **^1^**K1306. The system is prepared with a close trigger loop as follows. Bridge helix residues RPB1 835–864, Trigger loop segment RPB1 1101–1108, i + 1 GTP, MgA and MgB are aligned from PDB#2E2H into the system 5IY9-prel described above. Unconserved residues are mutated and GTP is mutated into CTP. Then twelve pre-min rounds are run with restraints on trigger loop section 1101-1108, active site CTP, MgA and MgB; followed up by one pre-min round with the restraints relieved. Then, the entire trigger loop domain is aligned into the structure from PDB#2E2H and non-conserved residues are mutated again. One pre-min round is run with restraints on trigger loop section RPB1 1101–1108, i + 1 CTP, MgA and MgB. Then, the trigger loop, the bridge helix, i + 1 CTP, MgA and MgB from the latter relaxed structure are aligned into a protein/nucleic structure taken from output aMD_A. Twenty rounds of pre-min are run with restraints on RPB1 1101–1108, i + 1 CTP, MgA and MgB, and one round with the restraints relieved. **^1^**K1306 transitive CTP is then aligned into the structure. Min1, min2, heat and eq, all with restraints on the transitive CTP, eq-vel and aMD, are computed with bond restraints on the i + 1/+3/+4 pairs and between trigger loop^**1**^1108:HE2 and i + 1 CTP:O5 atoms. **aMD_C1-4:** The initial system is an output of simulation aMD_B displaying a CTP bound at residue **^1^**K1306. **aMD_D1-15:** Three different protein structures are used as starting model, displaying different trigger loop conformations, taken from output aMD_A for aMD_D1 to 5, and two different output aMD_B frames for aMD_D6 to 10 and aMD_D11 to 15 respectively. A transitive CTP bound to residue **^2^**K211 from output aMD_C is aligned into the structure. Three, one and fifteen pre-min rounds with restraints on the transitive CTP are computed for simulations aMD_D1-5, D6-10 and D11-15, respectively. Then min1, min2, heat and eq, all with restraints on the transitive CTP, are computed. Finally, aMD are run with harmonic restraints on the hydrogen bond forming atoms of the i + 1, i and i − 1 pairs. **aMD_E1-6:** The initial system is an output of aMD_D displaying a CTP bound to the i + 2 register. The RNA chain is prolonged with a base added to the 3′end in the active site. Then six pre-min rounds are computed, followed up by min1, min2, heat, all with positional restraints on the transitive CTP, and eq-vel is run with bond harmonic restraints on the i + 2 CTP-dGMP pair. Then aMD is performed with the i + 2 CTP kept attached with a 0.3 kcal/mol harmonic bond. The i + 1 and i pairs are also restrained. **aMD_F1-6:** The initial system is a restart of output aMD_E displaying a CTP molecule isomerized at i + 2 position. aMD is run with the i + 2 bond restraints relieved. **aMD_F’1-6:** The initial system is the same output aMD_E as above, but with the nontemplate i + 4 register associated to its cognate template register prepared as follows. i + 4 folding is prepared by three short consecutive aMD/sMD runs, with mass constraints on the isomerized CTP and DNA residues. Then the normal aMD procedure is run with bond restraints on the i + 4 pair. **aMD_G1-2:** The initial system is taken from an output of aMD_A displaying a bent bridge helix, supplemented with the i + 2 isomerized CTP from output aMD_E aligned into the structure. The system is prepared with the RNA 3′end prolonged by one base, followed-up by eleven pre-min rounds with restraints on the isomerized CTP, one pre-min round with the restraints relieved, min1, min2 with restraints on the isomerized CTP, eq-vel and aMD with restraints on the i + 1 and i nucleic pairs. **aMD_H1-5:** The initial system is a simulation aMD_G restart frame displaying the beginning of the i + 2 NTP transfer to the active site together with forward translocation. **aMD_I1-3:** The initial system is a restart frame of aMD_H. **aMD_J1-7:** The initial system is a restart frame of aMD_I. **aMD_K1-2:** The initial system is taken from an output of simulation aMD_A displaying a stabilized TFIIF-protein complex. CTP-MgA is removed from the active site. A transitive CTP is aligned into the structure from an output of simulation aMD_A displaying a CTP inserted into CH2 corridor. Min1, min2, heat and eq-vel, are run with restraints on the CH2 corridor loaded transitive CTP. eq-vel and aMD are run with restraints on the i and i − 1 nucleic pairs. **aMD_L1-6:** The initial system is a restart frame of output aMD_K displaying the diffusion of the transitive CTP substrate to the catalytic cavity. **aMD_M1-3:** The initial system for aMD_M2 and M3 is an aMD_D output frame displaying the i + 3/+4 template DNA registers oriented towards the interior of CH3P. aMD_M3 is prepared by positioning three CTP molecules in front of i + 2 to i + 4 registers, then by twelve pre-min rounds with restraints on the downstream CTPs, followed up by heat also with restraints on the downstream CTPs, and by eq-vel and aMD both with bond restraints on the i + 1 to i + 4 hydrogen bonds formed by the i + 1 to i + 4 CTPs. aMD_M2 is prepared from the aMD_M3 pre-min structure by removing the i + 4 CTP. The i + 1 to i + 3 bonds are restrained. The initial system for aMD_M1 is a distinct aMD_D output frame displaying a CTP bound to the i + 2 DNA register and an adequate fork loop 2 conformation. The i + 1 and i + 2 bonds are restrained. **aMD_N1-4:** Alternative hybrid aMD/steered MD method. Simulation runs through CH3A/B/C/D are performed with a single boost acceleration (alphaD = 16,328 kJ/mol, EthreshD = 322,151 kJ/mol), 657,820 atoms and a CTP-MgB molecule in the active site. They are computed with mass constraints on DNA extremities. The diffusive CTP is pulled via a single atom and with an external force calculated as k*[(x − x_D_)^2^ + (y − y_D_)^2^ + (z − z_D_)^2^], where (x_D_, y_D_, z_D_) is the direction to which the force is applied and k = 0.03 kcal/mol Å^−2^ is the force magnitude. Min1, min2, heat and eq-vel, are run with restraints on the steered CTP. The initial protein structures are taken from simulation frames belonging to aMD_A, B and B’. The input is completed with a CTP positioned at the entrance of the respective subchannels.

## References

[1] Fu J., Gnatt A.L., Bushnell D.A., Jensen G.J., Thompson N.E., Burgess R.R., David P.R., Kornberg R.D. (1999). Yeast RNA polymerase II at 5 Å resolution. Cell.

[2] Zhang G., Campbell E.A., Minakhin L., Richter C., Severinov K., Darst S.A. (1999). Crystal structure of Thermus aquaticus core RNA polymerase at 3.3 Å resolution. Cell.

[3] Sosunov V., Sosunova E., Mustaev A., Bass I., Nikiforov V., Goldfarb A. (2003). Unified two-metal mechanism of RNA synthesis and degradation by RNA polymerase. EMBO J..

[4] Westover K.D., Bushnell D.A., Kornberg R.D. (2004). Structural basis of transcription: Nucleotide selection by rotation in the RNA polymerase II active center. Cell.

[5] Wang D., Bushnell D.A., Westover K.D., Kaplan C.D., Kornberg R.D. (2006). Structural Basis of Transcription: Role of the Trigger Loop in Substrate Specificity and Catalysis. Cell.

[6] Zenkin N., Yuzenkova Y., Severinov K. (2006). Transcript-assisted transcriptional proofreading. Science.

[7] Zhang Y., Degen D., Ho M.X., Sineva E., Ebright K.Y., Ebright Y.W., Mekler V., Vahedian-Movahed H., Feng Y., Yin R. (2014). GE23077 binds to the RNA polymerase ‘i’ and ‘i+1′ sites and prevents the binding of initiating nucleotides. eLife.

[8] Yu J., Da L.T., Huang X. (2015). Constructing kinetic models to elucidate structural dynamics of a complete RNA polymerase II elongation cycle. Phys. Biol..

[9] Batada N.N., Westover K.D., Bushnell D.A., Levitt M., Kornberg R.D. (2004). Diffusion of nucleoside triphosphates and role of the entry site to the RNA polymerase II active center. Proc. Natl. Acad. Sci. USA.

[10] Wang B., Sexton R.E., Feig M. (2017). Kinetics of nucleotide entry into RNA polymerase active site provides mechanism for efficiency and fidelity. Biochim. Biophys. Acta Gene Regul. Mech..

[11] Zhang L., Silva D.A., Pardo-Avila F., Wang D., Huang X. (2015). Structural Model of RNA Polymerase II Elongation Complex with Complete Transcription Bubble Reveals NTP Entry Routes. PLoS Comput. Biol..

[12] Brueckner F., Cramer P. (2008). Structural basis of transcription inhibition by α-amanitin and implications for RNA polymerase II translocation. Nat. Struct. Mol. Biol..

[13] Dangkulwanich M., Ishibashi T., Liu S., Kireeva M.L., Lubkowska L., Kashlev M., Bustamante C.J. (2013). Complete dissection of transcription elongation reveals slow translocation of RNA polymerase II in a linear ratchet mechanism. eLife.

[14] Zhang L., Pardo-Avila F., Unarta I.C., Cheung P.P.H., Wang G., Wang D., Huang X. (2016). Elucidation of the Dynamics of Transcription Elongation by RNA Polymerase II using Kinetic Network Models. Acc. Chem. Res..

[15] Foster J.E., Holmes S.F., Erie D.A. (2001). Allosteric binding of nucleoside triphosphates to RNA polymerase regulates transcription elongation. Cell.

[16] Holmes S.F., Erie D.A. (2003). Downstream DNA sequence effects on transcription elongation: Allosteric binding of nucleoside triphosphates facilitates translocation via a ratchet motion. J. Biol. Chem..

[17] Gong X.Q., Zhang C., Feig M., Burton Z.F. (2005). Dynamic error correction and regulation of downstream bubble opening by human RNA polymerase II. Mol. Cell.

[18] Xiong Y., Burton Z.F. (2007). A tunable ratchet driving human RNA polymerase II translocation adjusted by accurately templated nucleoside triphosphates loaded at downstream sites and by elongation factors. J. Biol. Chem..

[19] Kennedy S.R., Erie D.A. (2011). Templated nucleoside triphosphate binding to a noncatalytic site on RNA polymerase regulates transcription. Proc. Natl. Acad. Sci. USA.

[20] Nedialkov Y.A., Gong X.Q., Hovde S.L., Yamaguchi Y., Handa H., Geiger J.H., Yan H., Burton Z.F. (2003). NTP-driven translocation by human RNA polymerase II. J. Biol. Chem..

[21] Zhang C., Burton Z.F. (2004). Transcription factors IIF and IIS and nucleoside triphosphate substrates as dynamic probes of the human RNA polymerase II mechanism. J. Mol. Biol..

[22] Zhang C., Zobeck K.L., Burton Z.F. (2005). Human RNA Polymerase II Elongation in Slow Motion: Role of the TFIIF RAP74 α1 Helix in Nucleoside Triphosphate-Driven Translocation. Mol. Cell. Biol..

[23] Toulokhonov I., Zhang J., Palangat M., Landick R. (2007). A Central Role of the RNA Polymerase Trigger Loop in Active-Site Rearrangement during Transcriptional Pausing. Mol. Cell.

[24] Hamelberg D., De Oliveira C.A.F., McCammon J.A. (2007). Sampling of slow diffusive conformational transitions with accelerated molecular dynamics. J. Chem. Phys..

[25] Le Grand S., Götz A.W., Walker R.C. (2013). SPFP: Speed without compromise—A mixed precision model for GPU accelerated molecular dynamics simulations. Comput. Phys. Commun..

[26] Humphrey W., Dalke A., Schulten K. (1996). VMD: Visual molecular dynamics. J. Mol. Graph..

[27] Krieger E., Koraimann G., Vriend G. (2002). Increasing the precision of comparative models with YASARA NOVA—A self-parameterizing force field. Proteins Struct. Funct. Genet..

[28] Case D.A., Betz R., Botello-Smith W., Cerutti D.S., Cheatham T.E., Darden T.A., Duke R.E., Giese T.J., Gohlke H., Goetz A.W. (2016). AMBER 2016.

[29] Horn H.W., Swope W.C., Pitera J.W., Madura J.D., Dick T.J., Hura G.L., Head-Gordon T. (2004). Development of an improved four-site water model for biomolecular simulations: TIP4P-Ew. J. Chem. Phys..

[30] Horn H.W., Swope W.C., Pitera J.W. (2005). Characterization of the TIP4P-Ew water model: Vapor pressure and boiling point. J. Chem. Phys..

[31] Wang J., Cieplak P., Kollman P.A. (2000). How Well Does a Restrained Electrostatic Potential (RESP) Model Perform in Calculating Conformational Energies of Organic and Biological Molecules?. J. Comput. Chem..

[32] Pérez A., Marchán I., Svozil D., Sponer J., Cheatham T.E., Laughton C.A., Orozco M. (2007). Refinement of the AMBER force field for nucleic acids: Improving the description of α/γ conformers. Biophys. J..

[33] Zgarbová M., Otyepka M., Šponer J., Mládek A., Banáš P., Cheatham T.E., Jurečka P. (2011). Refinement of the Cornell et al. Nucleic acids force field based on reference quantum chemical calculations of glycosidic torsion profiles. J. Chem. Theory Comput..

[34] Krepl M., Zgarbová M., Stadlbauer P., Otyepka M., Banáš P., Koča J., Cheatham T.E., Jurečka P., Šponer J. (2012). Reference simulations of noncanonical nucleic acids with different χ variants of the AMBER FORCE Field: Quadruplex DNA, quadruplex RNA, and Z-DNA. J. Chem. Theory Comput..

[35] Zgarbová M., Luque F.J., Šponer J., Cheatham T.E., Otyepka M., Jurečka P. (2013). Toward improved description of DNA backbone: Revisiting epsilon and zeta torsion force field parameters. J. Chem. Theory Comput..

[36] Maier J.A., Martinez C., Kasavajhala K., Wickstrom L., Hauser K.E., Simmerling C. (2015). ff14SB: Improving the Accuracy of Protein Side Chain and Backbone Parameters from ff99SB. J. Chem. Theory Comput..

[37] Zgarbová M., Šponer J., Otyepka M., Cheatham T.E., Galindo-Murillo R., Jurečka P. (2015). Refinement of the Sugar-Phosphate Backbone Torsion Beta for AMBER Force Fields Improves the Description of Z- and B-DNA. J. Chem. Theory Comput..

[38] Panteva M.T., Giambaşu G.M., York D.M. (2015). Force Field for Mg^2+^, Mn^2+^, Zn^2+^, and Cd^2+^ Ions That Have Balanced Interactions with Nucleic Acids. J. Phys. Chem. B.

[39] Li P., Merz K.M. (2014). Taking into account the ion-induced dipole interaction in the nonbonded model of ions. J. Chem. Theory Comput..

[40] Li P., Song L.F., Merz K.M. (2015). Systematic parameterization of monovalent ions employing the nonbonded model. J. Chem. Theory Comput..

[41] Panteva M.T., Giambaşu G.M., York D.M. (2015). Comparison of structural, thermodynamic, kinetic and mass transport properties of Mg2+ ion models commonly used in biomolecular simulations. J. Comput. Chem..

[42] Meagher K.L., Redman L.T., Carlson H.A. (2003). Development of polyphosphate parameters for use with the AMBER force field. J. Comput. Chem..

[43] Demir Ö., Amaro R.E. (2012). Elements of nucleotide specificity in the trypanosoma brucei mitochondrial RNA editing enzyme RET2. J. Chem. Inf. Model..

[44] He Y., Yan C., Fang J., Inouye C., Tjian R., Ivanov I., Nogales E. (2016). Near-atomic resolution visualization of human transcription promoter opening. Nature.

[45] Yang J., Yan R., Roy A., Xu D., Poisson J., Zhang Y. (2015). The I-TASSER suite: Protein structure and function prediction. Nat. Methods.

[46] Huang X., Wang D., Weiss D.R., Bushnell D.A., Kornberg R.D., Levitt M. (2010). RNA polymerase II trigger loop residues stabilize and position the incoming nucleotide triphosphate in transcription. Proc. Natl. Acad. Sci. USA.

[47] Friedrichs M.S., Eastman P., Vaidyanathan V., Houston M., Legrand S., Beberg A.L., Ensign D.L., Bruns C.M., Pande V.S. (2009). Accelerating molecular dynamic simulation on graphics processing units. J. Comput. Chem..

[48] Eastman P., Pande V.S. (2010). Constant constraint matrix approximation: A robust, parallelizable constraint method for molecular simulations. J. Chem. Theory Comput..

[49] Eastman P., Pande V.S. (2010). Efficient nonbonded interactions for molecular dynamics on a graphics processing unit. J. Comput. Chem..

[50] Eastman P., Pande V.S. (2010). OpenMM: A Hardware-Independent Framework for Molecular Simulations. Comput. Sci. Eng..

[51] Eastman P., Friedrichs M.S., Chodera J.D., Radmer R.J., Bruns C.M., Ku J.P., Beauchamp K.A., Lane T.J., Wang L.P., Shukla D. (2013). OpenMM 4: A reusable, extensible, hardware independent library for high performance molecular simulation. J. Chem. Theory Comput..

[52] Varshney A., Brooks F.P., Wright W. (1994). V Computing smooth molecular surfaces. IEEE Comput. Graph. Appl..

[53] Frishman D., Argos P. (1995). Knowledge-based protein secondary structure assignment. Proteins Struct. Funct. Bioinforma..

[54] Stone J.E. (1998). Efficient Library for Parallel Ray Tracing and Animation. Master’s Thesis.

[55] Markwick P.R.L., McCammon J.A. (2011). Studying functional dynamics in bio-molecules using accelerated molecular dynamics. Phys. Chem. Chem. Phys..

[56] Miao Y., Sinko W., Pierce L., Bucher D., Walker R.C., McCammon J.A. (2014). Improved reweighting of accelerated molecular dynamics simulations for free energy calculation. J. Chem. Theory Comput..

[57] Miao Y., Feher V.A., McCammon J.A. (2015). Gaussian Accelerated Molecular Dynamics: Unconstrained Enhanced Sampling and Free Energy Calculation. J. Chem. Theory Comput..

[58] Kappel K., Miao Y., McCammon J.A. (2015). Accelerated molecular dynamics simulations of ligand binding to a muscarinic G-protein-coupled receptor. Q. Rev. Biophys..

[59] Ricci C.G., Chen J.S., Miao Y., Jinek M., Doudna J.A., McCammon J.A., Palermo G. (2019). Deciphering Off-Target Effects in CRISPR-Cas9 through Accelerated Molecular Dynamics. ACS Cent. Sci..

[60] Cramer P., Bushnell D.A., Fu J., Gnatt A.L., Maier-Davis B., Thompson N.E., Burgess R.R., Edwards A.M., David P.R., Kornberg R.D. (2000). Architecture of RNA polymerase II and implications for the transcription mechanism. Science.

[61] Kettenberger H., Armache K.J., Cramer P. (2003). Architecture of the RNA polymerase II-TFIIS complex and implications for mRNA cleavage. Cell.

[62] Murakami K.S., Shin Y., Turnbough C.L., Molodtsov V. (2017). X-ray crystal structure of a reiterative transcription complex reveals an atypical RNA extension pathway. Proc. Natl. Acad. Sci. USA.

[63] Molodtsov V., Sineva E., Zhang L., Huang X., Cashel M., Ades S.E., Murakami K.S. (2018). Allosteric Effector ppGpp Potentiates the Inhibition of Transcript Initiation by DksA. Mol. Cell.

[64] Zuo Y., Steitz T.A. (2015). Crystal structures of the E. coli transcription initiation complexes with a complete bubble. Mol. Cell.

[65] Wojtas M.N., Mogni M., Millet O., Bell S.D., Abrescia N.G.A. (2012). Structural and functional analyses of the interaction of archaeal RNA polymerase with DNA. Nucleic Acids Res..

[66] Miropolskaya N., Artsimovitch I., Klimašauskas S., Nikiforov V., Kulbachinskiy A. (2009). Allosteric control of catalysis by the F loop of RNA polymerase. Proc. Natl. Acad. Sci. USA.

[67] Miropolskaya N., Esyunina D., Klimašauskas S., Nikiforov V., Artsimovitch I., Kulbachinskiy A. (2014). Interplay between the trigger loop and the F loop during RNA polymerase catalysis. Nucleic Acids Res..

[68] Liu X., Farnung L., Wigge C., Cramer P. (2018). Cryo-EM structure of a mammalian RNA polymerase II elongation complex inhibited by α-amanitin. J. Biol. Chem..

[69] Walmacq C., Kireeva M.L., Irvin J., Nedialkov Y., Lubkowska L., Malagon F., Strathern J.N., Kashlev M. (2009). Rpb9 subunit controls transcription fidelity by delaying NTP sequestration in RNA polymerase II. J. Biol. Chem..

[70] Kettenberger H., Armache K.J., Cramer P. (2004). Complete RNA polymerase II elongation complex structure and its interactions with NTP and TFIIS. Mol. Cell.

[71] Vassylyev D.G., Vassylyeva M.N., Zhang J., Palangat M., Artsimovitch I., Landick R. (2007). Structural basis for substrate loading in bacterial RNA polymerase. Nature.

[72] Cramer P., Bushnell D.A., Kornberg R.D. (2001). Structural Basis of Transcription: RNA Polymerase II at 2.8 Angstrom Resolution. Science.

[73] Ruprich-Robert G., Thuriaux P. (2010). Non-canonical DNA transcription enzymes and the conservation of two-barrel RNA polymerases. Nucleic Acids Res..

[74] Weinzierl R.O.J. (2010). Nanomechanical constraints acting on the catalytic site of cellular RNA polymerases. Biochem. Soc. Trans..

[75] Domecq C., Kireeva M., Archambault J., Kashlev M., Coulombe B., Burton Z.F. (2010). Site-directed mutagenesis, purification and assay of Saccharomyces cerevisiae RNA polymerase II. Protein Expr. Purif..

[76] Hein P.P., Landick R. (2010). The bridge helix coordinates movements of modules in RNA polymerase. BMC Biol..

[77] Seibold S.A., Singh B.N., Zhang C., Kireeva M., Domecq C., Bouchard A., Nazione A.M., Feig M., Cukier R.I., Coulombe B. (2010). Conformational coupling, bridge helix dynamics and active site dehydration in catalysis by RNA polymerase. Biochim. Biophys. Acta Gene Regul. Mech..

[78] Wang L., Zhou Y., Xu L., Xiao R., Lu X., Chen L., Chong J., Li H., He C., Fu X.D. (2015). Molecular basis for 5-carboxycytosine recognition by RNA polymerase II elongation complex. Nature.

[79] Chang J.W., Wu Y.M., Chen Z.Y., Huang S.H., Wang C.H., Wu P.L., Weng Y.P., You C., Piehler J., Chang W.H. (2013). Hybrid electron microscopy-FRET imaging localizes the dynamical C-terminus of Tfg2 in RNA polymerase II-TFIIF with nanometer precision. J. Struct. Biol..

[80] Schweikhard V., Meng C., Murakami K., Kaplan C.D., Kornberg R.D., Block S.M. (2014). Transcription factors TFIIF and TFIIS promote transcript elongation by RNA polymerase II by synergistic and independent mechanisms. Proc. Natl. Acad. Sci. USA.

[81] Da L.T., Wang D., Huang X. (2012). Dynamics of pyrophosphate ion release and its coupled trigger loop motion from closed to open state in RNA polymerase II. J. Am. Chem. Soc..

[82] Chakraborty A., Wang D., Ebright Y.W., Korlann Y., Kortkhonjia E., Kim T., Chowdhury S., Wigneshweraraj S., Irschik H., Jansen R. (2012). Opening and Closing of the Bacterial RNA Polymerase Clamp. Science.

[83] Schulz S., Gietl A., Smollett K., Tinnefeld P., Werner F., Grohmann D. (2016). TFE and Spt4/5 open and close the RNA polymerase clamp during the transcription cycle. Proc. Natl. Acad. Sci. USA.

[84] Duchi D., Mazumder A., Malinen A.M., Ebright R.H., Kapanidis A.N. (2018). The RNA polymerase clamp interconverts dynamically among three states and is stabilized in a partly closed state by ppGpp. Nucleic Acids Res..

[85] Geiger S.R., Lorenzen K., Schreieck A., Hanecker P., Kostrewa D., Heck A.J.R., Cramer P. (2010). RNA Polymerase I Contains a TFIIF-Related DNA-Binding Subcomplex. Mol. Cell.

[86] Taylor N.M.I., Baudin F., Von Scheven G., Müller C.W. (2013). RNA polymerase III-specific general transcription factor IIIC contains a heterodimer resembling TFIIF Rap30/Rap74. Nucleic Acids Res..

[87] Wu C.-C., Lin Y.-C., Chen H.-T. (2011). The TFIIF-Like Rpc37/53 Dimer Lies at the Center of a Protein Network To Connect TFIIIC, Bdp1, and the RNA Polymerase III Active Center. Mol. Cell. Biol..

[88] Kubicek C.E., Chisholm R.D., Takayama S., Hawley D.K. (2013). RNA Polymerase II Mutations Conferring Defects in Poly(A) Site Cleavage and Termination in Saccharomyces cerevisiae. G3 Genes Genomes Genet..

[89] Mullen Davis M.A., Guo J., Price D.H., Luse D.S. (2014). Functional interactions of the RNA polymerase II-interacting Proteins Gdown1 and TFIIF. J. Biol. Chem..

[90] DeLaney E., Luse D.S. (2016). Gdown1 associates efficiently with RNA polymerase II after promoter clearance and displaces TFIIF during transcript elongation. PLoS ONE.

[91] Jishage M., Yu X., Shi Y., Ganesan S.J., Chen W.Y., Sali A., Chait B.T., Asturias F.J., Roeder R.G. (2018). Architecture of Pol II(G) and molecular mechanism of transcription regulation by Gdown1. Nat. Struct. Mol. Biol..

