# Supplementary material for: Nucleotide Loading Modes of Human RNA Polymerase II as Deciphered by Molecular Simulations
Source: Biomolecules. 2020 Sep 7;10(9):1289. doi: 10.3390/biom10091289 (PMC7565877; doi:10.3390/biom10091289)
Supplement: Supplementary file 1 [file biomolecules-10-01289-s001.pdf]

Supplementary Materials for the article:

Nucleotide Loading Modes of Human RNA Polymerase II as Deciphered by Molecular Simulations

Nicolas E.J. Génin and Robert O.J. Weinzierl

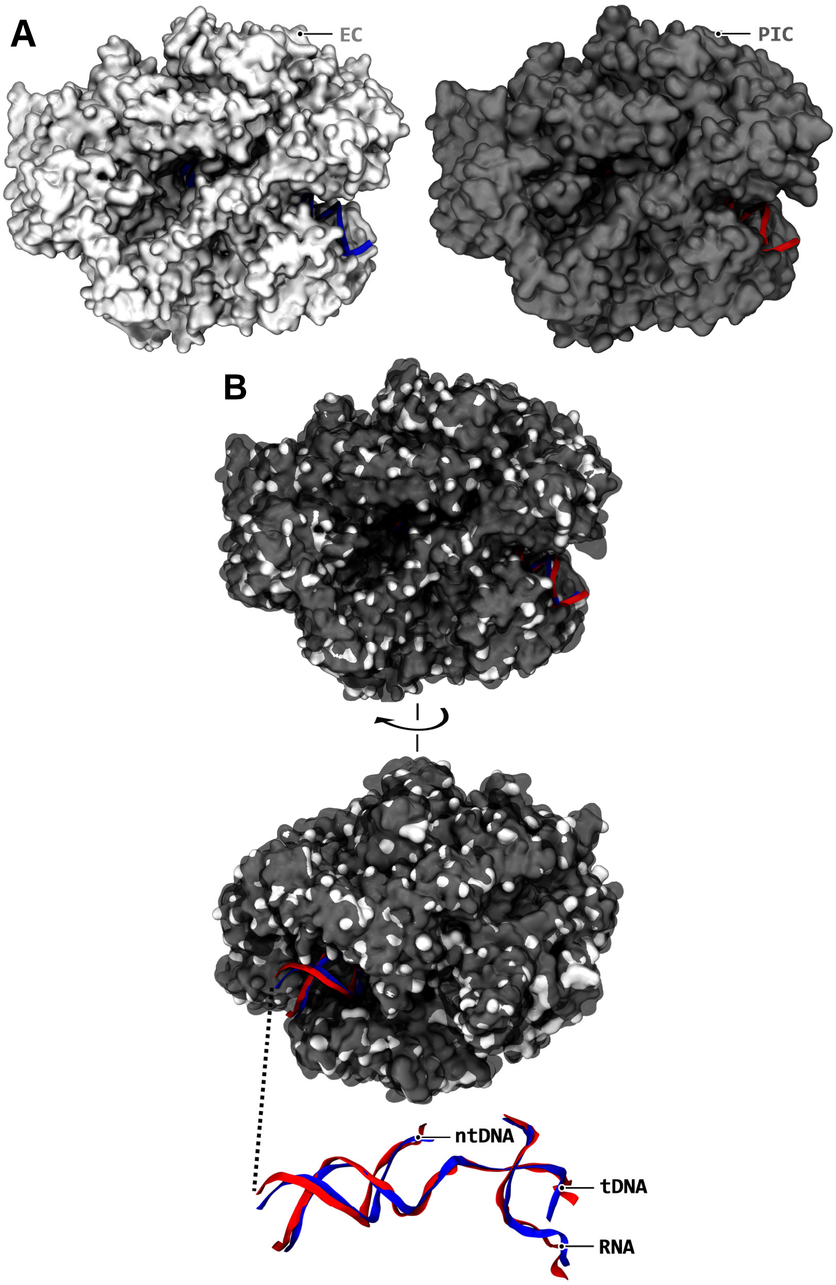

Figure S1. Structural alignment of the 10 subunit preinitiation and elongation RNAPII complexes. (**A**) The x-ray structures of the PDB#5IY9 preinitiation complex ("PIC", grey) and the PDB#5FLM elongation complex ("EC", white) are represented. (**B**) The overlapping of the crystal structures is shown. The nucleic acids lying inside the enzymatic complex for the PIC (red) and the EC (blue) are represented: tDNA i + 16 to i - 8, ntDNA i + 16 to i + 3 and RNA i to i - 13 sections. The ntDNA i + 2 to i - 8 section is not resolved in the EC structure (initiation factors such as TFIIF typically allow to resolve the entire ntDNA strand due to their stabilization effect). The PIC and the EC display very high structural similarity, as characterized by a total nucleic acid/polypeptide backbone RMSD of 1.50 Å.

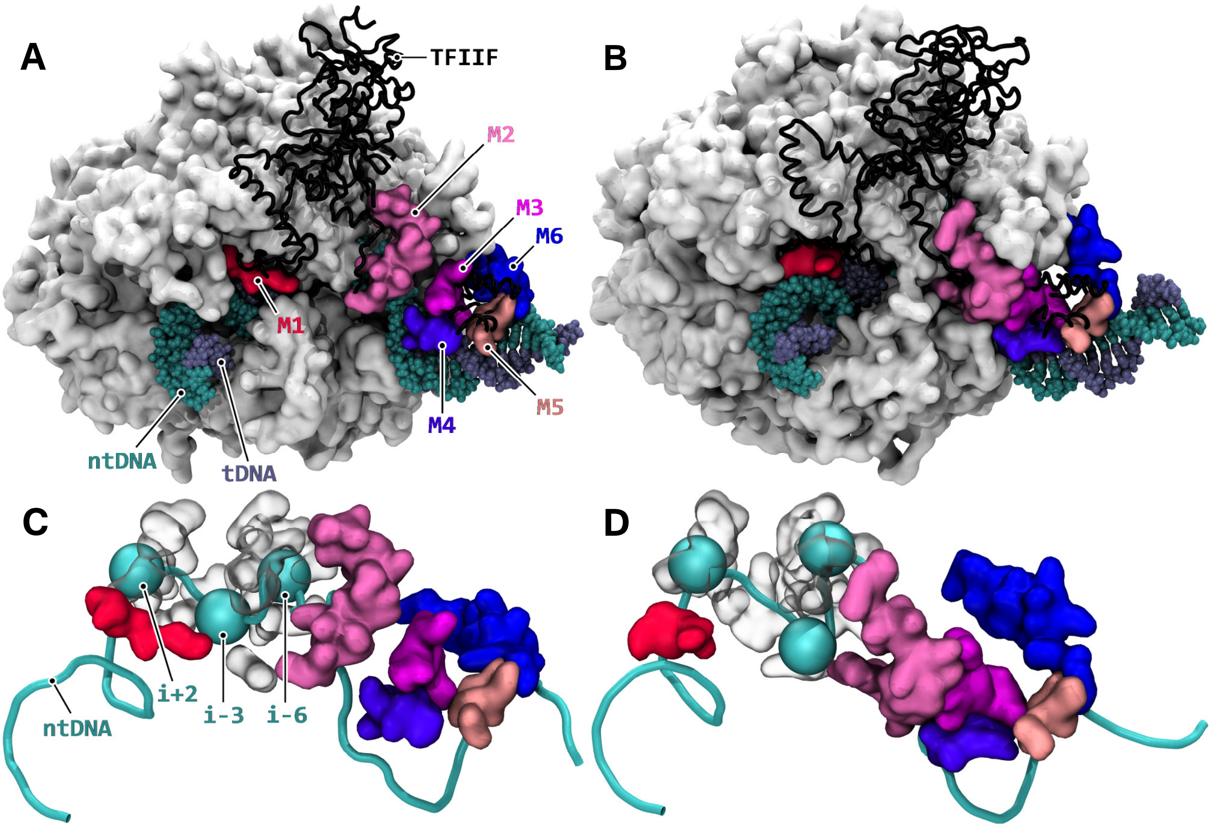

Figure S2. TFIIF reconfiguration of the downstream bubble. Left side (A, C) and right side (B, D): configuration before (simulation aMD_A1: 15 ns) and after (aMD_A1: 220 ns) the relaxation of TFIIF. (**A**, **B**) TFIIF (black tube) interacts with nontemplate (cyan) and template DNA (fade blue) through six Modules: 1 (red), 2 (dark pink), 3 (magenta), 4 (purple), 5 (light pink) and 6 (blue). Amino acids RAP30 180, 233, 234, belonging to Modules 4 and 6 are not represented. RNAPII is represented in white. (**C**, **D**) The anchoring of Module 1 to downstream ntDNA, together with the re-arrangement of Modules 2 to 4, cause the folding of ntDNA. Module 5 remains stable, while Module 6 shifts position and modifies its interaction with upstream tDNA (not represented). The edges of the fold about the i + 2, i - 3 and i - 6 registers are represented as beads. Nontemplate DNA, represented as a tube spanning across the backbone phosphorus atoms, undergoes switching of interactions inside the protein. The amino acids involved in the interaction switch about the fold (RPB1 326, 327; RPB2 206, 208, 210, 211, 223–225, 229, 230, 232, 233, 261, 262, 338, 342, 345, 346, 349, 383–385, 405, 409, 453, 457, 458, 461, 463, 464, 488–493, 495, 497) are represented as transparent surfaces.

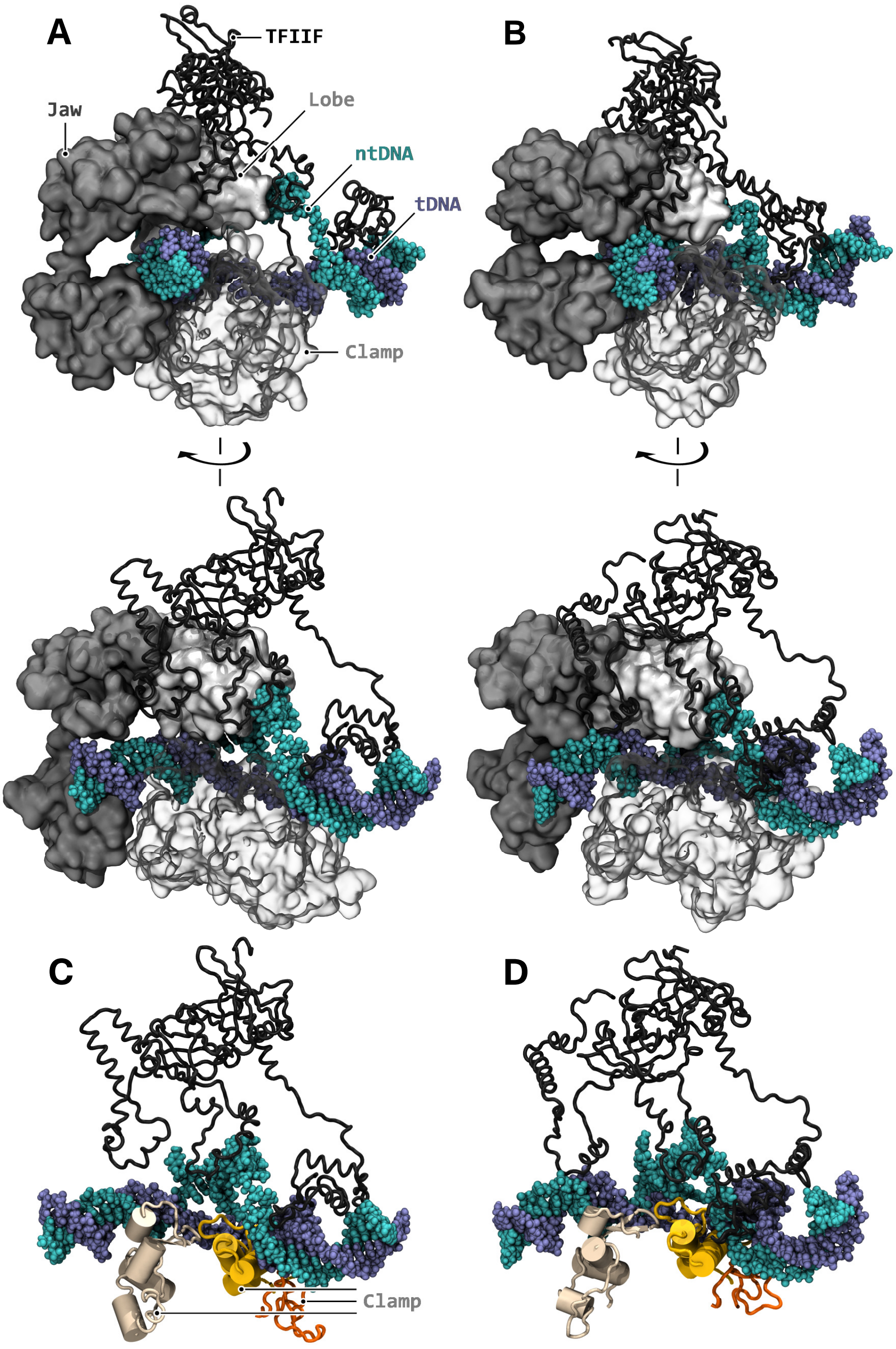

Figure S3. CH1 stabilization into the close conformation. Left side (A, C) and right side (B, D): configuration before (simulation aMD_A1: 12 ns) and after (aMD_A1: 141 ns) the relaxation of TFIIF. The template and nontemplate DNA strands are shown in cyan and fade blue atomic representation respectively. TFIIF is represented as a black tube. (**A**, **B**) The jaw, RPB1 1164–1300, RPB5 1–140, RPB9 11–49 (grey surface), and lobe, RPB2: 200–392 (white surface), domains fold against DNA, locking the downstream channel, CH1, into close conformation. The clamp domain, RPB1 5–360, 1425–1466, RPB2 1107–1173 (transparent surface), remains stacked against the downstream helix, but undergoes re-arrangements. (**C**, **D**) The clamp domain re-organization is visualized through three regions, bordering the DNA helix, shown in composite tube and cylinder representation: RPB1 134–215 (beige), 262–360 (yellow) and 27–71 (orange).

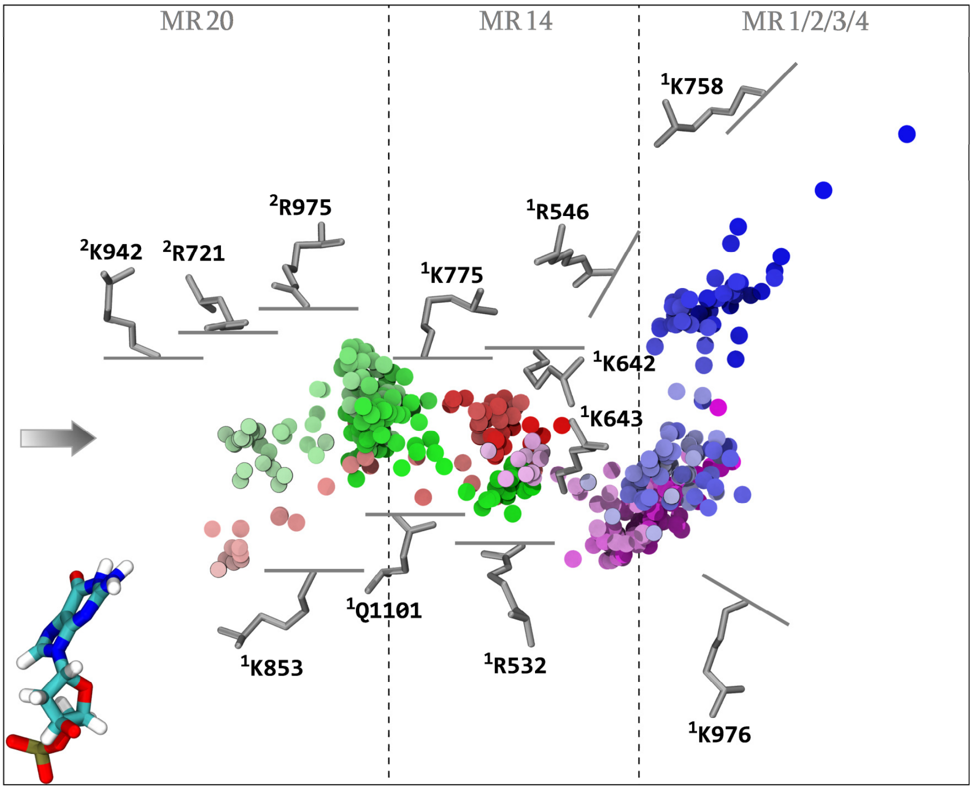

Figure S4. CH2 exit traces. Four exit trajectories across the CH2 pathway are displayed, where the NTP-Mg center of mass (colored disc) is displayed every simulation frame. Key amino acids delineating the NTP interaction pathway are represented as grey sticks. Active site i + 1 template register is shown in atomic type representation. Red, green, blue and magenta trajectories are from aMD_K1 (36-61.5 ns), aMD_K2 (4-98.5 ns), aMD_E5 (43-95 ns) and aMD_A4 (34-79.5 ns), respectively.

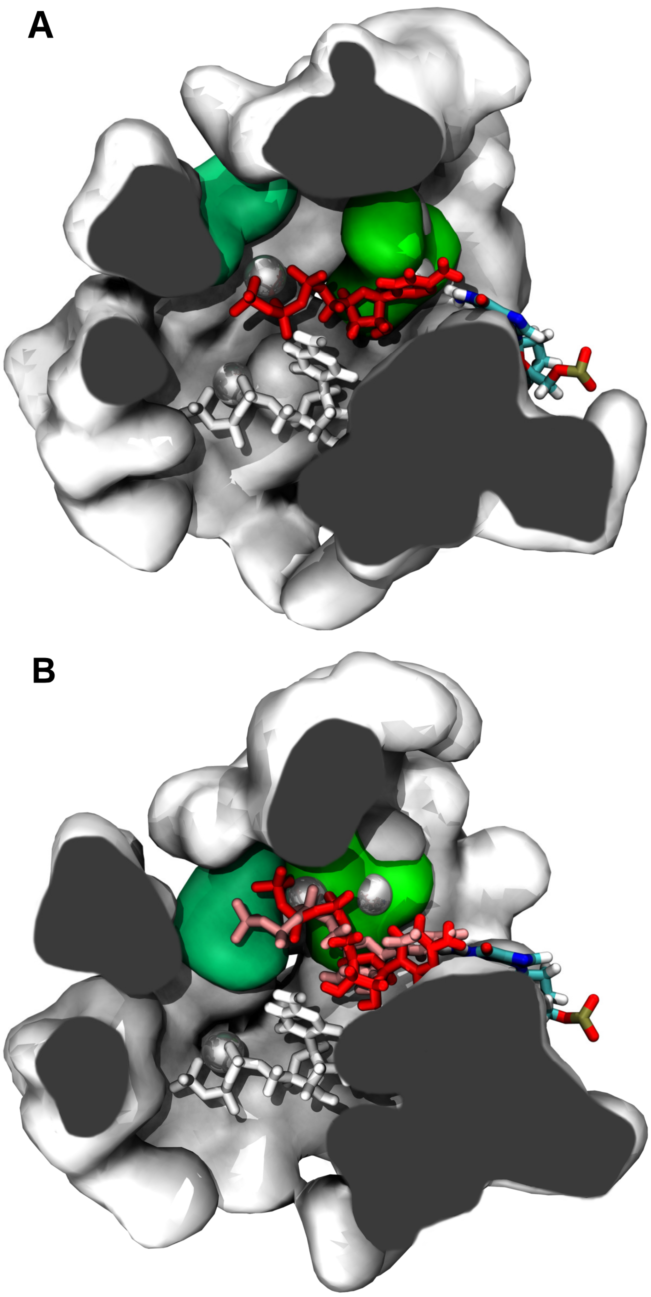

Figure S5. Active site loading transitions. The ultimate section of the CH2 pathway (CH2 corridor) leading to the active site and accommodating the last diffusion state transitions of the incoming NTP to successfully binds to the catalytic register, is presented. CH2 corridor is displayed as a cutaway view (white surface). The NTP bound MgB and catalytic MgA atoms are represented as silver spheres. The i + 1 template dNMP is colored by atomic type in stick representation. The metal site is constituted by an aspartic triad ^{1}D495/^{1}D497/^{1}D499 and by ^{2}E791/^{2}D792, represented as fluo green and green surfaces respectively. The NTP molecule along the diffusion trajectory is represented from white to red (A) or white to pink to red (B) chronologically. (**A**) The NTP advances from the entrance of the corridor (aMD_K2, 0 ns) to i + 1 binding (aMD_L1, 35 ns) directly, without inverted E site coordination and with only residual interaction with the metal site. (**B**) The NTP transitions from the corridor (aMD_K2, 0 ns) directly to i + 1 binding (aMD_L2, 5 ns), with weak metal site interaction, and then binds to the metal site (aMD_L2: 31.5 ns time point displayed; the process initiates at 10 ns), supporting the fact that magnesium binding can proceed after i + 1 binding.

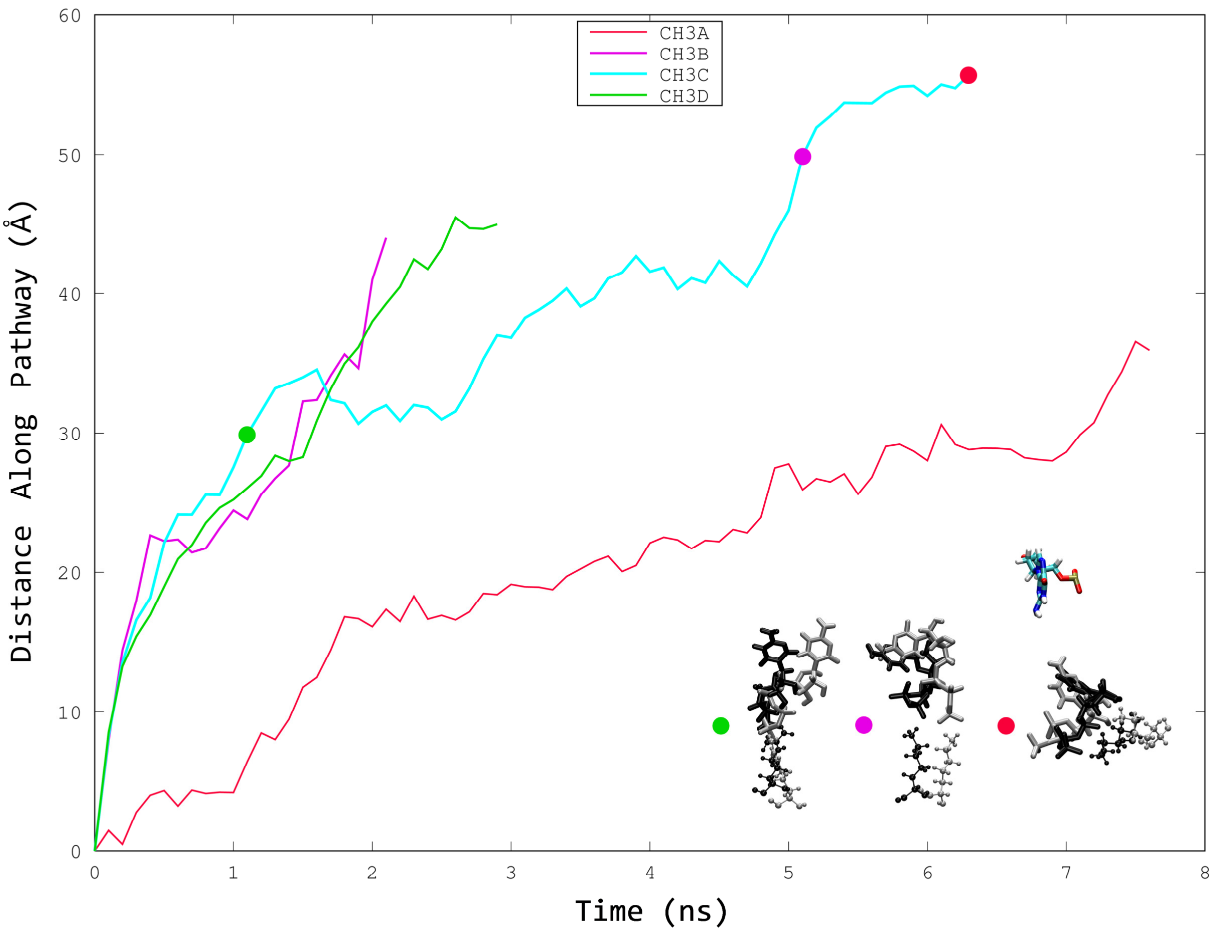

Figure S6. Comparative biased reaction-coordinate diffusion through CH1 subpathways. The green, magenta and red discs represent the merging of CH3A, CH3B and CH3D steering paths with CH3C, at amino acid positions [1]K1135, [1]K1306 and [1]R1345 respectively: CH3A 7.6 ns, CH3B 2.1 ns and CH3D 2.9 ns frames respectively merge CH3C 6.3 ns, 5.1 ns and 1.1 ns frames. Respectively superimposed NTP-coordinating amino acids [1]K1135, [1]K1306 and [1]R1345 are shown in black CPK representation for the CH3C reference trajectory and in silver for the CH3A, CH3B and CH3D trajectories, while the superimposed NTPs are shown in stick representation (black, CH3C, silver, CH3A/B/D).

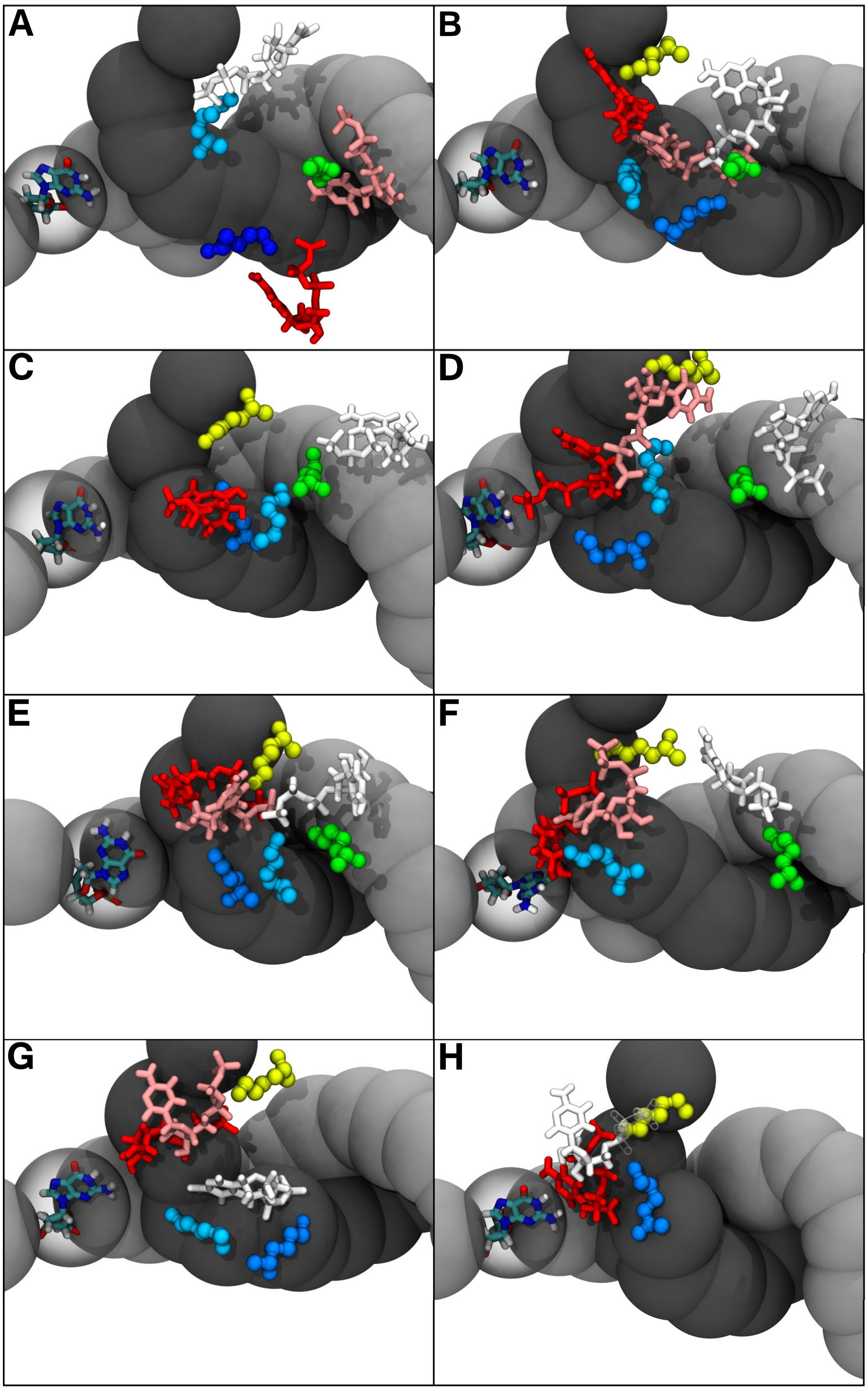

Figure S7. CH3P state transitions. The NTP molecule is shown from white to pink to red or white to red chronologically. Amino acids ²K211 (yellow), ¹K1133 (medium blue), ¹K1135 (cyan), ¹K1306 (green), ¹K1132 (dark blue) are represented. The downstream template and nontemplate strands are represented as light and dark grey beads respectively. The i + 1 template register is shown in sticks colored by atomic type surrounded by a transparent bead. The following transitions are displayed. (**A**) aMD_B2 (79.75, 81.5, 86.75 ns). (**B**) aMD_C1 (0.25, 1.75, 6.5 ns). (**C**) aMD_B3 (21.5, 24, 46 ns). (**D**) aMD_B1 (6.75, 14.5, 20 ns). (**E**) aMD_D13 (4, 16, 18.5 ns). (**F**) aMD_D8 (3.5, 16, 19.5 ns). (**G**) aMD_B'1 (0.5, 15, 16 ns). (**H**) aMD_D12 (1, 9 ns).

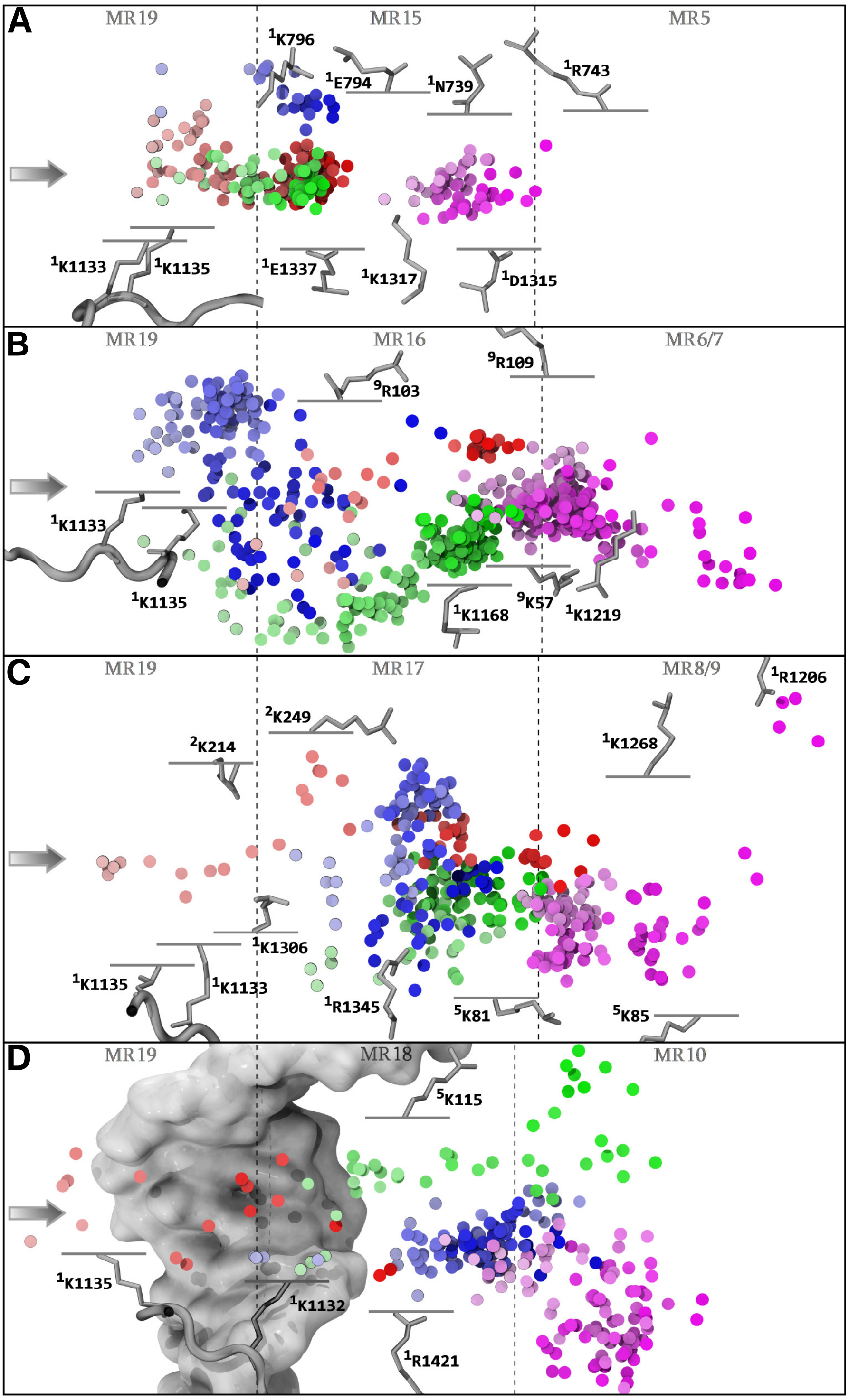

Figure S8. CH1 exit traces. Four exit trajectories are displayed for each CH1 subpathway: CH3A (A), CH3B (B), CH3C (C), CH3D (D), where the NTP-Mg center of mass (colored disc) is displayed every simulation frame. Key amino acids delineating the NTP interaction pathway are represented as grey sticks. The trigger loop carboxy-terminal section, RPB1 1129–1137, is represented as a grey tube. The downstream DNA helix partially enclosing a section of the CH3D (D) pathway is represented as a white surface. Red, green, blue and magenta trajectory simulations are the following. (**A**) aMD_B4 (4-32.5 ns), aMD_F′2 (27-52.5 ns), aMD_D4 (0-12 ns), aMD_A4 (54.5-79.5 ns). (**B**) aMD_C4 (1-8.5 ns), aMD_B′2 (2.5-84 ns), aMD_B′1 (1-71 ns), aMD_A6 (258-350 ns). (**C**) aMD_D8 (70.5-93 ns), aMD_C3 (2-22 ns), aMD_C3 (0-24 ns), aMD_B2 (33.5-61 ns). (**D**) aMD_B2 (80-84.5 ns), aMD_C4 (1-11 ns), aMD_C2 (0.5-23.5 ns), aMD_A6 (197-250.5 ns).

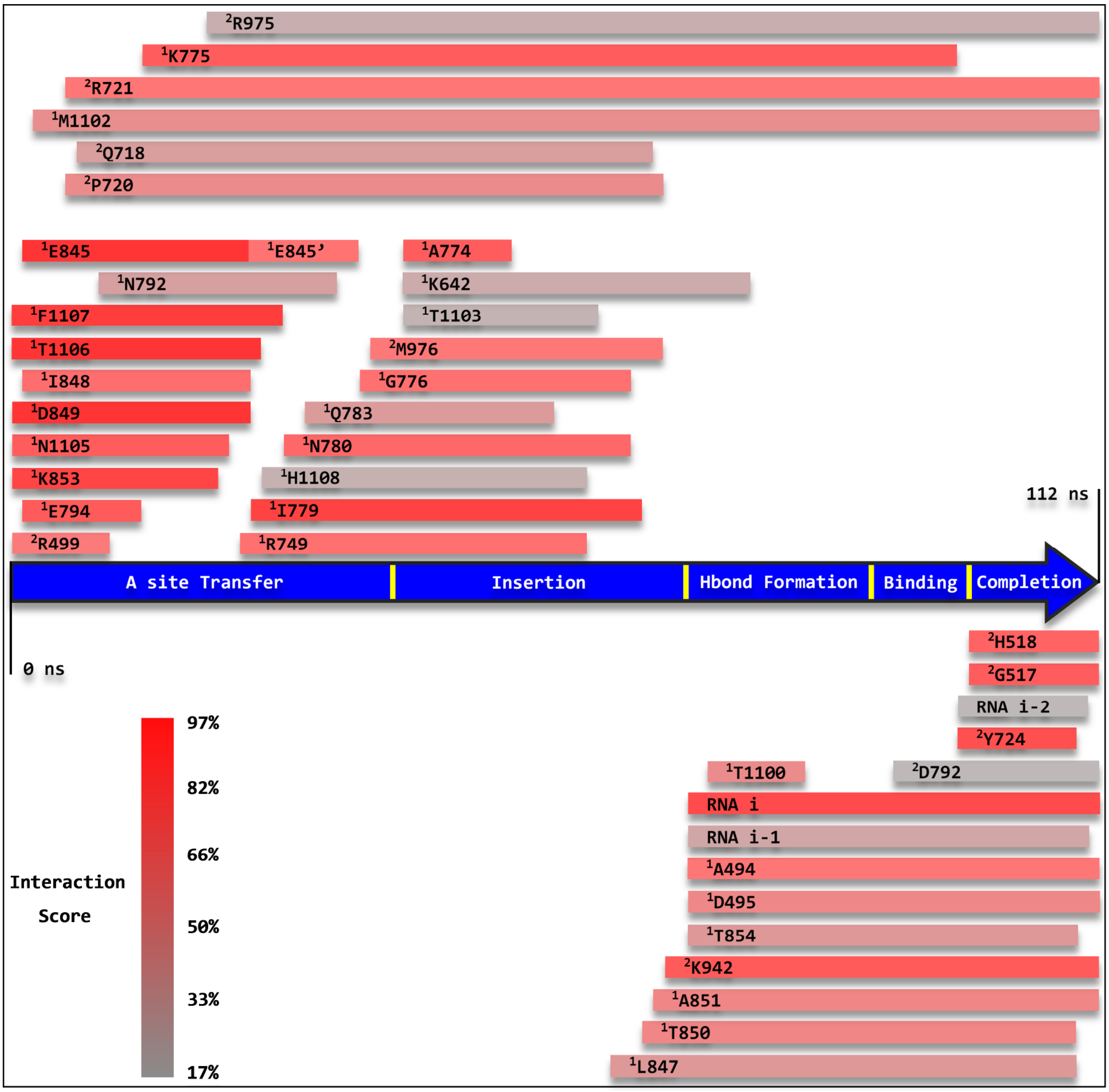

Figure S9. i + 2 NTP transfer and reassociation with the translocated i + 2 template register. The main amino/nucleic acids interacting with NTP-MgB during the shuttling to the active site occurring simultaneously to translocation, are indicated as rectangles. The interaction frequency with the substrate is color-coded from grey to red. ^{1}E845 and ^{1}E845′ denote different coordinations with NTP-MgB: the former interacts mainly with MgB, while the latter interacts only with NTP hydrogens. Regarding the other negatively charged residues, ^{1}E794, ^{1}D495 interact with NTP-MgB indistinctly, ^{1}D849 mainly with NTP hydrogens, and ^{2}D792 with MgB. The combined trajectory is aMD_E4 (starts at 56 ns time point), aMD_G1 (0-11.4 ns), aMD_H2 (0-55.8 ns), aMD_I3 (0-21.4 ns), aMD_J4 (0-23.4 ns), where aMD_G1 is a restart of aMD_E4 (56 ns) with a bent bridge helix, aMD_H2, I3 and J4 are restarts of aMD_G1 (11.4 ns), H2 (55.8 ns) and I3 (21.4 ns) respectively.

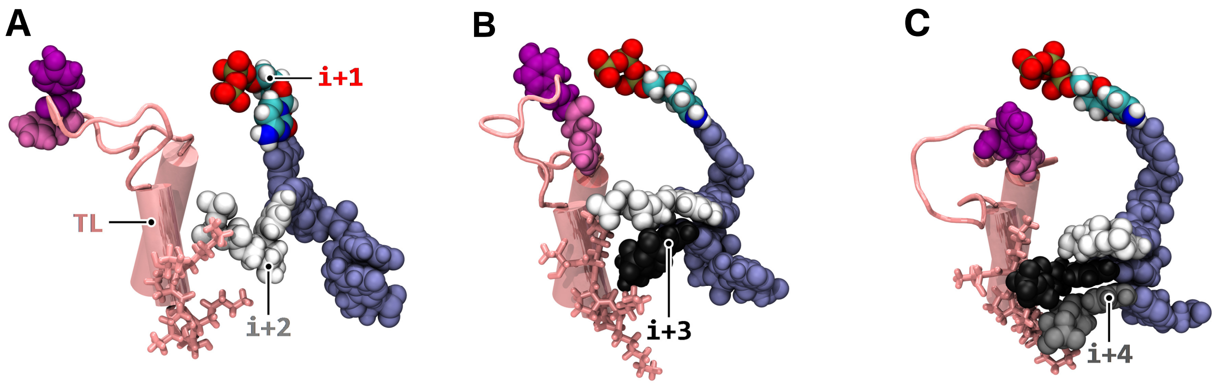

Figure S10. Coupling of downstream binding with trigger loop closing. (**A**, **B**, **C**) Respective simulated configuration after 100 ns with i + 2 (aMD_M1), i + 2/ +3 (aMD_M2) and i + 2/ +3/ +4 (aMD_M3) NTPs paired to template DNA. Template DNA (fade blue van der Waals spheres) is represented from i + 4 to i + 1 positions. Active site i + 1 NTP is colored by atomic type. i + 2, i + 3 and i + 4 NTPs are in white, black and grey van der Waals spherical representation respectively. The trigger loop RPB1 1087–1130 portion is in pink composite tube and cylinder secondary structure representation. The NTPs bound to the downstream registers, interact with the C-TER portion of the trigger loop: RPB1 1131–1136 (pink sticks). Central residues [1]H1108 (dark pink) and [1]Y1109 (magenta) characterize potential trigger loop tip rotation induced by the downstream NTPs anchoring the trigger loop carboxy-terminal portion.

Table S1. Amino acid contribution to the locking of the i + 2 register.

Residue	Domain	Interaction (%)
^{2}K494	FL2	92.09
^{1}E856	BH	88.68
^{1}V852	BH	72.70
^{1}Y859	BH	71.57
^{1}A855	BH	57.23
^{2}R499	FL2	53.27
^{2}L495	FL2	28.88
^{1}K853	BH	25.08
^{2}G493	FL2	24.57
^{2}A496	FL2	22.97
^{2}H518	FL3	17.07
^{1}R351	SW2	14.72
^{1}R1416	SW1	8.88
^{1}R349	SW2	7.66

The interaction score is derived from the statistical analysis of simulations aMD_B to M (excluding translocation frames).

Table S2. Amino acid sequence of the pathway interlining residues.

Channel	Sequence
CH2	RPB1:364 366 460-462 464 465 467 468 486-502 527 529-539 542 601-629 637 641-655 658 701 704 705 708 709 711 712 715 716 718-720 722-733 735 736 738-740 742-755 757 758 767 770-779 781-783 787-792 795-797 822 842-847 849-855 857-858 901-933 936 940 944 946 949-951 953 954 956-961 963-979 982 984 985 1037 1038 1040 1041 1043 1044 1047 1048 1050-1053 1056-1059 1096-1121 1136-1158 1304 1306-1339 1343 1347-1349 1351 1353-1360 1364 1365 1368 1369 1375-1377 1379-1381 1383-1395 1398 1399, RPB2:516-518 520 718 720-724 727 728 731 790-792 932 934 940-944 968 971-976 1051-1053 1058, RPB5:142-148 194-202, RPB8:97-113 115 122 124 126 128 129
Corridor	RPB1:364 366 460-462 464 465 467 468 486-502 527 529 534-539 542 642 646 647 773-776 842-847 849-855 857-858 1096-1121, RPB2: 516-518 520 718 720-724 727 728 731 790-792 932 934 940-944 968 971-976 1051-1053 1058
CH3A	RPB1:728-732 734-743 745 746 749 779 788-805 813 815 820-822 841 842 844-853 856 857 859 860 863 1101-1123 1125 1126 1129-1138 1140 1306-1312 1314-1320 1335-1340 1358 1360-1362 1381 1382 1384-1389 1414-1417, RPB2:208-214 380-387 391 494-504 520
CH3B	RPB1:720-739 741 742 791-806 812-815 820 841 842 844-853 856 857 859 860 863 1103-1123 1125 1126 1129-1139 1155 1158 1159 1162-1165 1167-1172 1174 1176 1213 1215-1225 1253-1256 1294 1303 1305-1311 1337-1341 1360-1362 1414-1417, RPB2:207-218 239 294-296 299 300 375-387 391 494-504 520 608 671, RPB9:54-69 96-113 120-125
CH3C	RPB1:729-732 734-739 742 743 792-805 813 815 820 841 842 844-853 856 857 859 860 863 1103-1109 1111-1123 1125 1126 1129-1140 1162-1165 1167 1168 1170 1206 1233-1243 1260-1279 1283 1284 1286-1298 1300-1309 1311 1337-1342 1344-1346 1348 1359-1364 1366-1368 1414-1417, RPB2:208-218 240-255 300 375-387 391 494-504 520, RPB5:1-3 5 6 9 10 13 47-51 52-57 59 75-86 88-90 93 106-115 131-134 136, RPB9:54 56 100-106, TFIIF-1:164-180
CH3D	RPB1:18-24 105 108 118 122-147 149-153 234-237 239-242 729-732 734-739 742 743 792-805 813 815 820 841 842 844-853 856 857 859 860 863 880-886 1103-1109 1111-1123 1125 1126 1129-1140 1301-1304 1306-1309 1311 1337-1346 1348 1358-1372 1374 1406 1408-1429 1439 1442 1445-1453 1456 1457 1462, RPB2:208-218 240-242 244-251 300 375-387 391 494-504 520 1172-1174, RPB5:9 13 20 24 67-69 77 79-82 87 90 94 98-101 103 104 106-139 151-153 156 160 164-178 180-182 184-190 208-210, RPB6:109-114, RPB9:100-106
CH3P	RPB1:792-795 798 799 801 802 841 842 844-853 856 857 859 860 863 1103-1108 1113-1123 1125 1126 1129-1137 1360-1362 1414-1417, RPB2:208-214 382-387 391 494-504 520

The amino acids composing the solvent-exposed surface of the different pathways are listed. The sequences were refined by a variant of the pathway-exploration algorithm to scan the interlining atoms along the respective pathway axes.

Table S3. Amino acid sequence of the NTP binding macro-regions.

Area	Main NTP coordinating residues
MR1	^{1}K758 ^{1}K697 ^{1}K708 ^{1}R743 ^{1}K707 ^{1}D695 ^{1}K751 ^{1}D701 ^{1}N704 ^{1}Q700 ^{1}S759 ^{1}K710 ^{1}D712 ^{1}D747 ^{1}S696 ^{1}Q711 ^{1}S754 ^{1}E715 ^{1}D750 ^{1}Q703 ^{1}K719 ^{1}T698 ^{1}I744 ^{1}S755 ^{1}Q740 ^{1}Y699 ^{1}I714 **^{1}K775 ^{1}R749 ^{1}N739 ^{1}N746 ^{1}E724 ^{1}E726 ^{1}T728 Funnel-loop**
MR2	^{1}R604 ^{8}K20 ^{1}K550 ^{8}R98 ^{1}K547 ^{2}Q1040 ^{8}E100 ^{1}T686 ^{8}D23 ^{2}S1039 ^{2}R1023 ^{8}K21 ^{1}T605 ^{8}F22 ^{8}E103 ^{1}E683 ^{1}I603 ^{8}D102 ^{2}Y1020 ^{8}R24 ^{2}N1025 ^{1}D552 ^{8}G101 ^{8}E18 ^{8}R27 ^{8}G19 ^{8}Y97 ^{8}R124 ^{8}T104 ^{1}G688 ^{8}Y115 ^{1}D691 ^{1}C602 ^{1}R551 ^{8}R111 ^{1}N601 ^{8}K13 ^{8}D16 ^{8}D11 ^{1}V629 ^{1}E631 ^{1}M637 ^{8}E33 ^{1}I639 ^{8}K55 ^{8}K36 ^{1}H685 ^{8}E31 ^{8}S105 ^{8}F35 ^{8}T106 ^{8}E107 ^{8}T110 ^{1}I687 ^{8}N131 ^{8}D38 ^{8}Q126 ^{1}S692 ^{8}L122 **^{1}R546 CH2-loop Funnel-loop(1&2)**
MR3	^{1}K919 ^{1}K918 ^{1}R931 ^{1}R1052 ^{1}K910 ^{1}K914 ^{1}R928 ^{1}R967 ^{1}D923 ^{1}R921 ^{1}R960 ^{1}S912 ^{1}L909 ^{1}R1053 ^{1}N913 ^{1}T972 ^{1}R963 ^{1}A915 ^{1}E1057 ^{1}P971 ^{1}N905 ^{1}T925 ^{1}Y924 ^{1}E917 ^{1}P911 ^{1}R954 ^{1}E964 ^{1}L906 ^{1}E957 ^{1}V968 ^{1}E1056 ^{1}A929 ^{1}E953 ^{1}K976 ^{1}N926 ^{1}F970 ^{1}R932 ^{1}Q904 ^{1}E927 ^{1}G973 ^{1}R958 ^{1}E951 ^{1}N950 ^{1}L930 ^{1}F922 ^{1}I969 ^{1}E961 ^{1}A907 ^{1}Q935 ^{1}D974 ^{1}K940 ^{1}F916 ^{1}T908 ^{1}L984 ^{1}A946 ^{1}S975 ^{1}F956
MR4	^{1}R1380 ^{1}K1329 ^{1}E1324 ^{1}I1322 ^{1}D1325 ^{5}E198 ^{1}E1327 ^{5}E143 ^{1}R1356 ^{1}F1328 ^{1}T1323 ^{1}K1376 ^{1}L1331 ^{1}Q1332 ^{1}G1326 ^{1}E1333 ^{1}V1355 ^{1}D1353 **^{1}Q1146 ^{1}R1149 ^{1}D1150 ^{1}G1145 ^{1}S1147 ^{1}L1144 Cleft-loop**
MR5	^{1}R1380 ^{1}K708 ^{1}K1329 ^{1}R743 ^{1}K751 ^{1}E1324 ^{1}I1322 ^{1}K710 ^{1}D1325 ^{1}D712 ^{1}D747 ^{1}Q711 ^{1}E1327 ^{1}E715 ^{1}D750 ^{1}R1356 ^{1}F1328 ^{1}T1323 ^{1}K719 ^{1}L1331 ^{1}Q1332 ^{1}G1326 ^{1}I744 ^{1}Q740 ^{1}E1333 ^{1}I714 **^{1}R749 ^{1}N739 ^{1}N746 ^{1}E724 ^{1}E726 ^{1}T728 ^{1}Q1146 ^{1}R1149 ^{1}D1150 ^{1}G1145 ^{1}S1147 ^{1}L1144 Cleft-loop**
MR6	^{9}K92 ^{9}R122 ^{9}E82 ^{9}E125 ^{9}E93 ^{9}H118 ^{9}K88 ^{9}H91 ^{9}H121 ^{9}D83 ^{9}R80 ^{9}Q74 ^{9}C119 ^{9}Y111 ^{9}W123 ^{9}C89 ^{9}E64 ^{9}T124 ^{9}Q87 ^{9}G120 ^{9}P117 ^{9}G90 ^{1}N723 ^{9}C114 ^{9}I68 ^{9}Q67 ^{1}N722 ^{9}V113 ^{9}E61 ^{9}P85 ^{9}C86 ^{9}H60 ^{9}Y112 ^{9}D63 ^{9}I69 ^{9}L65 ^{9}H84 ^{9}S73 ^{9}T66 ^{9}T59 ^{9}D71 ^{9}T115 ^{1}H721 ^{9}V62 ^{9}I58 **^{9}K57 ^{1}E724 ^{1}E726 ^{1}T728 RPB9-loop**
MR7	^{1}K1219 ^{1}R1218 ^{1}E1253 ^{1}H1220 ^{1}K1254 ^{1}D1250 ^{1}D1223 ^{1}E1188 ^{1}D1217 ^{1}E1191 ^{1}R1224 ^{1}T1222 ^{1}N1251 ^{1}K1155 ^{1}D1249 ^{1}E1215 **^{1}K1225 ^{1}E1152 ^{1}E1198 ^{1}Y1196 ^{1}V1195 ^{1}N1194 ^{1}Y1197**
MR8	^{1}R1153 ^{1}R1206 ^{1}E1233 ^{1}Q1230 ^{1}K1268 ^{1}K1234 ^{1}K1350 ^{1}S1264 ^{1}D1351 ^{1}E1229 ^{1}E1272 ^{1}N1267 ^{1}D1265 ^{1}F1202 ^{1}E1271 ^{1}T1227 ^{1}D1156 ^{1}R1160 ^{1}Q1299 ^{1}E1274 ^{1}D1203 ^{1}N1263 ^{1}D1241 ^{1}D1242 ^{1}K1278 ^{1}M1262 ^{1}F1239 ^{1}E1273 ^{1}D1277 ^{1}G1238 ^{1}G1300 ^{1}E1266 ^{1}V1276 ^{1}P1200 ^{1}L1226 ^{1}D1201 ^{1}Q1270 ^{1}M1269 ^{1}G1240 ^{1}L1298 ^{1}N1236 ^{1}M1199 ^{1}V1275 ^{1}N1244 **^{1}K1225 ^{1}Q1146 ^{1}R1149 ^{1}D1150 ^{1}E1152 ^{1}G1145 ^{1}S1147 ^{1}L1144 ^{1}E1198 ^{1}Y1196 ^{1}V1195 ^{1}N1194 ^{1}Y1197**
MR9	^{5}M1 ^{5}K85 ^{5}K88 ^{5}Q92 ^{5}R93 ^{5}V89 ^{5}D2 ^{5}R55 ^{5}E5 ^{5}R52 ^{5}E78 ^{5}E96 ^{5}E4 ^{5}Q95 ^{5}E79 ^{5}D3 ^{5}K47 ^{5}P53 ^{5}E6 ^{5}R9 ^{5}T56 ^{5}E50 ^{5}T86 ^{5}C91 ^{5}R54 ^{5}E97 ^{5}P48 ^{5}G51 ^{5}D46 ^{5}T59 ^{5}K41 ^{5}L61 ^{5}S49 ^{5}D57 ^{5}F73 ^{5}E38 ^{5}Y90 ^{5}E39 ^{5}Q43 ^{5}V60 ^{5}I84 ^{5}S44 ^{5}F75 ^{5}G83 ^{5}L136 ^{5}L37 ^{5}T36 ^{5}G45 ^{5}Y8 ^{5}P77
MR10	^{5}R187 ^{1}K132 ^{5}R172 ^{5}Q210 ^{1}R1408 ^{1}Q136 ^{1}K134 ^{1}D1423 ^{5}K186 ^{1}S133 ^{1}D128 ^{1}K125 ^{5}K124 ^{5}D120 ^{1}G135 ^{1}E155 ^{1}A131 ^{1}K127 ^{5}P123 ^{1}K151 ^{5}I126 ^{5}G188 ^{1}F234 ^{5}V119 ^{1}P124 ^{1}I129 ^{5}A122 ^{1}L130 ^{5}L208 ^{5}Y125 ^{1}R140 ^{5}R101 ^{1}E158 ^{5}M121 ^{1}N122 ^{1}D120 ^{5}Q189 ^{1}E159 ^{5}P68 ^{1}N123 ^{5}D67 ^{1}V119 ^{5}T100 ^{5}T69 ^{5}D66 ^{1}G156 ^{1}G157 **^{5}Q116 ^{1}K139**
MR11	^{1}R33 ^{2}R1141 ^{1}R241 ^{1}E30 ^{2}L1121 ^{2}R1170 ^{2}C1140 ^{2}C1122 ^{1}Y242 ^{2}G1139 ^{2}N1142 ^{2}R1138 ^{1}D29 ^{2}K1143 ^{2}R1131 ^{2}Q1145 ^{1}W247 ^{1}S27 ^{1}M34 ^{2}E1136 ^{1}K32 ^{2}I1124 ^{2}N1120 ^{2}T1144 ^{1}E38 ^{1}P240 ^{2}A1126 ^{2}C1137 ^{1}D230 ^{1}R244 ^{2}T1130 ^{1}K226 ^{2}H1133 ^{1}S35 ^{1}V36 ^{1}R227 ^{1}E239 ^{2}T1134 ^{1}P28 ^{2}G1123 ^{1}E231 ^{2}Y1135 ^{1}E246 ^{1}T37 ^{2}N1129 ^{2}T1132
MR12	^{1}R16 ^{1}K19 ^{2}M1172 ^{5}R166 ^{2}V1174 ^{1}R20 ^{1}K1452 ^{6}Y109 ^{1}D1472 ^{1}T17 ^{6}R107 ^{5}R162 ^{5}N168 ^{5}K164 ^{2}R1150 ^{1}N884 ^{6}G113 ^{6}D112 ^{2}S1173 ^{1}P1465 ^{6}Y115 ^{1}P1450 ^{2}R1104 ^{1}C1470 ^{1}I883 ^{6}P111 ^{5}E158 ^{5}E167 ^{5}Q169 ^{5}K153 ^{1}K874 ^{1}L1463 ^{6}S114 ^{1}Q1462 ^{1}Q22 ^{1}G1469 ^{1}F1471 ^{1}Y875 ^{1}S1448 ^{1}Q885 ^{6}R64 ^{6}L110 ^{5}A161 ^{1}E1447 ^{1}S882 ^{1}D876 ^{1}L1473 ^{5}L165 ^{1}M1451 ^{2}S1147 ^{1}R880 ^{1}N1457 ^{5}T157 ^{5}L160 ^{1}D1449 ^{5}E154 ^{5}T152
MR13	^{1}R1031 ^{1}K1014 ^{1}D1026 ^{1}P1028 ^{1}K1018 ^{1}Q1032 ^{1}E1011 ^{5}K192 ^{5}E155 ^{1}N1024 ^{5}I204 ^{1}E1035 ^{1}E1015 ^{1}K1019 ^{5}Y206 ^{1}S1017 ^{1}G1025 ^{1}D1027 ^{5}M151 ^{1}I1007 ^{1}N1042 ^{1}L1029 ^{1}V1021 ^{5}L159 ^{1}T1038 ^{1}L1039 ^{1}S1030 ^{1}N1036
MR14	^{1}K643 ^{1}S648 ^{1}G650 ^{1}A649 ^{1}S644 ^{1}R532 ^{1}S651 ^{1}F903 ^{1}K642 ^{1}C641 ^{1}D1389 ^{1}T647 ^{1}Q783 ^{1}G536 ^{1}N780 ^{1}Q539 ^{1}V538 ^{1}V534 ^{1}I537 ^{1}Y1392 ^{1}S1387 ^{1}G1390 ^{1}Y1383 ^{1}V788 ^{1}P533

	^{1}M535 ^{1}S1391 **^{1}R546 ^{1}K775 ^{1}F1388 ^{1}H1384 ^{1}R749 ^{1}N746 F-loop-central(2) CH2-loop TL-central Funnel-loop(2&3)**
MR15	^{1}E738 ^{1}R820 ^{1}M1309 ^{1}E1337 ^{1}N742 **^{1}R749 ^{1}N739 ^{1}N746 ^{1}Q735 ^{1}R734 ^{1}N731 ^{1}T732 ^{1}F1388 ^{1}H1384 TL-central F-loop-central Cleft-loop**
MR16	^{1}K1168 ^{1}E1162 **^{1}R1167 ^{1}K1306 ^{1}Y1308 ^{1}S1305 ^{1}V1307 ^{1}D1339 ^{1}G1340 ^{9}K57 ^{1}Q735 ^{1}R734 ^{1}N731 ^{1}T732 RPB9-loop**
MR17	^{5}K81 ^{1}R1345 ^{5}P80 ^{5}Q108 ^{2}K249 ^{1}Q1303 ^{2}G246 ^{1}T1294 ^{1}N1291 ^{2}Q245 ^{1}S1290 ^{5}Q107 ^{1}D1295 ^{1}E1302 ^{1}I1301 ^{2}R242 ^{2}S250 ^{5}Q133 ^{2}G244 ^{2}K248 ^{5}L131 ^{5}G109 ^{1}C1287 ^{1}M1296 ^{1}M1344 ^{2}G243 ^{1}H1163 ^{2}Q254 ^{5}T111 ^{1}T1297 ^{1}V1341 **^{1}S1305 ^{1}G1340 ^{1}R1167 ^{5}Q116**
MR18	^{1}H1410 ^{1}R1421 ^{5}Q174 ^{1}H143 ^{1}Q1422 ^{5}A175 ^{5}G176 ^{5}K115 ^{1}T142 ^{1}D146 ^{1}T1367 ^{1}K138 ^{1}F1366 ^{5}Q129 ^{5}E128 ^{5}R181 ^{1}M1412 ^{5}L118 ^{1}V1363 ^{1}K149 **^{1}K1132 ^{1}K139 NTER-SW1**
MR19	^{1}K1135 ^{2}A496 ^{1}K1133 ^{1}K1125 ^{2}R499 ^{2}K494 ^{2}Q500 ^{1}I848 ^{2}K211 ^{1}E1126 ^{2}K497 ^{1}P1134 ^{1}P1122 ^{1}N1129 ^{1}S1131 ^{2}L495 ^{1}P1137 ^{2}K214 ^{1}S1138 ^{1}T1136 ^{1}E856 ^{2}D212 ^{2}N503 ^{2}S213 ^{2}D386 ^{2}Y215 ^{2}H502 ^{1}R1123 ^{1}R863 ^{1}N1360 ^{2}Y217 ^{2}K210 **^{1}K1132 ^{1}K1306 ^{1}Y1308 ^{1}S1305 ^{1}V1307 ^{1}D1339 ^{1}G1340 TL-central F-loop-central(1) NTER-SW1 BH-central**
MR20	^{1}D495 MgA ^{2}R721 ^{1}D497 ^{2}K942 ^{2}R975 ^{2}E791 ^{1}R460 ^{1}T854 ^{1}P462 ^{1}D499 ^{2}E516 ^{1}N493 ^{2}G517 ^{2}D792 ^{1}T850 ^{1}A494 ^{2}Y724 ^{1}L464 ^{2}H518 ^{1}G498 ^{1}L847 ^{2}T723 ^{2}Q718 ^{2}S974 ^{1}E500 ^{1}F496 ^{2}P720 ^{1}G846 ^{2}S719 ^{2}M976 ^{1}P491 **^{1}K775 BH-central TL-central**
TL-central	^{1}K1115 ^{1}H1108 ^{1}Q1101 ^{1}M1102 ^{1}Y1109 ^{1}L1104 ^{1}T1106 ^{1}T1103 ^{1}S1113 ^{1}V1112 ^{1}T1100 ^{1}N1105 ^{1}E1097 ^{1}F1107 ^{1}G1111 ^{1}N1116 ^{1}V1117 ^{1}L1119 ^{1}T1118
BH-central	^{1}K853 ^{1}A851 ^{1}D849 ^{1}V852 ^{1}E845 ^{1}A855
F-loop-central	(1): ^{1}E794 ^{1}K796 ^{1}N792 ^{1}G795 ^{1}R797 ^{1}F800 ^{1}P799 ^{1}I798 (2): ^{1}Q791 ^{1}Q790
Funnel-loop	(1): ^{1}Y763 ^{1}N765 ^{1}E762 ^{1}S761 ^{1}N764 ^{1}L760 (2): ^{1}K767 ^{1}S768 ^{1}Q757 ^{1}A774 ^{1}V771 ^{1}G773 ^{1}S772 (3): ^{1}G776 ^{1}K778 ^{1}I779 ^{1}S777
Cleft-loop	^{1}K1319 ^{1}K1318 ^{1}K1317 ^{1}I1320 ^{1}I1321 ^{1}D1315 ^{1}N1316 ^{1}T1314
RPB9-loop	^{9}R109 ^{9}R103 ^{9}E105 ^{9}A104 ^{9}D106 ^{9}H100 ^{9}F97 ^{9}S101 ^{9}Q98 ^{9}S99
CH2-loop	^{1}H606 ^{1}K627 ^{1}K619 ^{1}H620 ^{1}P623 ^{1}I621 ^{1}Y618 ^{1}D611 ^{1}S622 ^{1}G624 ^{1}D614 ^{1}D625 ^{1}P617 ^{1}H609 ^{1}T626 ^{1}S615 ^{1}D612 ^{1}E613 ^{1}P610 ^{1}T608
NTER-SW1	^{1}T1415 ^{1}R1416 ^{1}I1414 ^{1}H1417

The NTP binding macro-regions (MR) 1 to 20 are indicated in the Area column. The main NTP interacting residues defining each macro-region are listed by order of statistical relevance (i.e., by order of NTP binding affinity). The highly flexible domains are specified in the last rows. Duplicate residues belonging to the latter domains are indicated in bold at the end of each MR row. The other flexible or junction amino acids are also indicated in bold. They belong or are in proximity to the following motifs: RPB1 α4, α21, α38, α39, α40, α42, α46, β32, β33, RPB5 α6, RPB9 linker. The duplicate residues ^{1}K1132 and ^{1}K775 constitute respectively extruding parts of the TLc and funnel loop domains. The MR5 residues that are duplicates with MR1 and MR4 solely are not specified in bold as they do not represent flexible or junction amino acids as such but result from the fact that CH3A entry pathway overlaps with CH2. The catalytic MgA atom, bound to ^{1}D495/^{1}D497/^{1}D499, is considered as belonging to the protein and is listed.

Table S4. Amino/nucleic acid contribution to the pre-isomerization and isomerization states.

State	Residue	Domain	Interaction (%)
Pre-isomerization	nti+4	ntDNA	87.80
	nti+3	ntDNA	80.96
	^{2}A496	FL2	70.43
	^{1}K1133	TL	59.15
	^{2}K211	na	59.15
	^{1}K1135	TL	48.43
	^{2}K497	FL2	43.81
	^{2}R499	FL2	34.20
	^{2}K494	FL2	31.79
	^{1}K853	BH	25.14
	^{1}K1125	TL	24.77
	^{2}Q500	FL2	24.58
	^{1}K1132	TL	21.26
	^{1}P1134	TL	15.34
	^{2}L495	FL2	8.87
Isomerization	^{1}K853	BH	95.50
	^{1}K1115	TL	73.92
	^{1}K1125	TL	72.98
	^{1}E1126	TL	67.35
	nti+4	ntDNA	66.23
	^{2}A496	FL2	64.54
	^{1}K1135	TL	55.72
	^{1}P1122	TL	53.47
	^{1}K1133	TL	42.78
	^{2}Q500	FL2	27.77
	^{2}R499	FL2	27.20
	^{1}N1129	TL	18.76
	^{1}E794	F-loop	16.51
	^{1}D849	BH	15.76
	^{2}K494	FL2	15.38
	^{1}I848	BH	13.32
	^{2}K497	FL2	8.26
	^{1}K796	F-loop	7.69

The interaction score is derived from the statistical analysis of the simulations in the pre-isomerization state: aMD_E2 (0-25 ns), E3 (0-46, 53.5-75 ns), E4 (0-39.5 ns), E5 (0-39.5 ns), E6 (0-59 ns), and in the isomerization state: aMD_E3 (46.5-53 ns), E4 (40-76.5 ns), aMD_F2 (0-73.5 ns), F5 (0-72.5 ns), F6 (0-75 ns).

Table S5. Melting state of the downstream registers.

State	Melting score (%)			
	i+3 full	i+3 partial	i+4 full	i+4 partial
TFIIF Stab.	9.26	5.57	72.76	3.23
TFIIF Unstab.	76.86	11.30	65.52	11.13
Translocation	7.84	9.81	22.72	15.98
NTP in CH3P	29.34	20.47	98.70	1.30

Were excluded from the analysis all the simulations comprising harmonic restraints on i + 2, i + 3 or i + 4 DNA pairs. The melting states are divided into "full": the three hydrogen bond forming atoms between guanine and cytidine DNA pairs at i + 3/ +4 positions are all above 3 Å distance, and "partial": only one or two hydrogen bond forming atom pairs are within 3 Å distance. The i + 2 melting state is not displayed as it virtually permanently melted (100 % of the simulation frames in the "TFIIF Stabilized," "Translocation" and "NTP in CH3P" states, 99.29% of the time in the "TFIIF Unstabilized" state). The enzymatic states are the following. The "TFIIF Stabilized (Stab.)" state corresponds to the simulation frames where TFIIF is in equilibrated conformation: simulations aMD_A1, B, C, D, K, L. The forward translocation frames are treated separately in the "Translocation" state (simulations aMD_G, H, I, J). The "TFIIF Unstabilized (Unstab.)" state applies to simulations aMD_A2 to A6, where TFIIF is only partially properly associated to the transcription bubble. The "NTP in CH3P" state corresponds to simulations aMD_F1 to F6, comprising an NTP remaining in CH3P. Comparing the three former states with the latter is of particular interest to gauge the relationship between CH1 loading and the induced melting of the downstream registers. The "Translocation" and "NTP in CH3P" states involve TFIIF in equilibrated conformation.

Table S6. RPB1 subunit conservation.

```
  1
h MHGGGPPSGD SACPLRTIKR VQFGVLSPDE LKRMSVTEGG IKYPETTEGG ..RPKLGGLM DPRQGVIERT GRCQTCAGNM TECPGHFGHI
m MHGGGPPSGD SACPLRTIKR VQFGVLSPDE LKRMSVTEGG IKYPETTEGG ..RPKLGGLM DPRQGVIERT GRCQTCAGNM TECPGHFGHI
d ....MSTPTD SKAPLRQVKR VQFGILSPDE IRRMSVTEGG VQFAETMEGG ..RPKLGGLM DPRQGVIDRT SRCQTCAGNM TECPGHFGHI
c ...MALVGVD FQAPLRIVSR VQFGILGPEE IKRMSVAH.. VEFPEVYENG ..KPKLGGLM DPRQGVIDRR GRCMTCAGNL TDCPGHFGHL
y ....MVGQQY SSAPLRTVKE VQFGLFSPEE VRAISVAK.. IRFPETMDET QTRAKIGGLN DPRLGSIDRN LKCQTCQEGM NECPGHFGHI
s .......... ..MSEKNIKG IKFGILSPDE IRKMSVTA.. IITPDVYDED GT.PIEGSVM DPRLGVIEPG QKCPTCGNTL GNCPGHFGHI

  91
h ELAKPVFHVG FLVKTMKVLR CVCFFCSKLL VDSNNPKIKD ILAKSKGQPK KRLTHVYDLC KGKNICEGGE EMDNKFGVEQ PEGDEDLTKE
m ELAKPVFHVG FLVKTMKVLR CVCFFCSKLL VDSNNPKIKD ILAKSKGQPK KRLTHVYDLC KGKNICEGGE EMDNKFGVEQ PEGDEDLTKE
d DLAKPVFHIG FITKTIKILR CVCFYCSKML VSPHNPKIKE IVMKSRGQPR KRLAYVYDLC KGKTICEGGE DMDLTKENQQ P....DPNKK
c ELAKPVFHIG FLTKTLKILR CVCFYCGRLL IDKSAPRVLE ILKKTGTNSK KRLTMIYDLC KAKSVCEGAA EKEEGMPDDP DDPMND..GK
y DLAKPVFHVG FIAKIKKVCE CVCMHCGKLL LDEHNELMRQ ALAIK..DSK KRFAAIWTLC KTKMVCETDV PSED...... .....DPTQL
s ELVRPVIHVG FVKHVYEFLK ATCRRCGRVK ISE....... .......DEI EKYSRIYNAI KKRWPSAARR LTEYVK.... ....KTAMKA

  181
h KGHGGCGRYQ PRIRRSGLEL YAEWK..HVN EDSQEKKI.L LSPERVHEIF KRISDEECFV LGMEPRYARP EWMIVTVLPV PPLSVRPAVV
m KGHGGCGRYQ PRIRRSGLEL YAEWK..HVN EDSQEKKI.L LSPERVHEIF KRISDEECFV LGMEPRYARP EWMIVTVLPV PPLSVRPAVV
d PGHGGCGHYQ PSIRRTGLDL TAEWK..HQN EDSQEKKI.V VSAERVWEIL KHITDEECFI LGMDPKYARP DWMIVTVLPV PPLAVRPAVV
c KVAGGCGRYQ PSYRRVGIDI NAEWKK.NVN EDTQERKI.M LTAERVLEVF QQITDEDILV IGMDPQFARP EWMICTVLPV PPLAVRPAVV
y VSRGGCGNTQ PTIRKDGLKL VGSWKKDRAT GDADEPELRV LSTEEILNIF KHISVKDFTS LGFNEVFSRP EWMILTCLPV PPPPVRPSIS
s QVCPHCGEKQ FKIKL..... ....EKPYNF YEERKEGVAK LTPSDIRERL EKVPESDVEI LGYDPTTSRP EWMILTVLPV PPITIRPSIM

  271
h MQGSARNQDD LTHKLADIVK INNQLRRNEQ NGAAAHVIAE DVKLLQFHVA TMVDNELPGL PRAMQKSGRP LKSLKQRLKG KEGRVRGNLM
m MQGSARNQDD LTHKLADIVK INNQLRRNEQ NGAAAHVIAE DVKLLQFHVA TMVDNELPGL PRAMQKSGRP LKSLKQRLKG KEGRVRGNLM
d MFGAAKNQDD LTHKLSDIIK ANNELRKNEA SGAAAHVIQE NIKMLQFHVA TLVDNDMPGM PRAMQKSGKP LKAIKARLKG KEGRIRGNLM
c TFGSAKNQDD LTHKLSDIIK TNQQLQRNEA NGAAAHVLTD DVRLLQFHVA TLVDNCIPGL PTATQKGGRP LKSIKQRLKG KEGRIRGNLM
y FNESQRGEDD LTFKLADILK ANISLETLEH NGAPHHAIEE AESLLQFHVA TYMDNDIAGQ PQALQKSGRP VKSIRARLKG KEGRIRGNLM
s IESGIRAEDD LTHKLVDIVR INERLKESID AGAPQLIIED LWDLLQYHVA TYFDNEIPGL PPSKHRSGRP LRTLAQRLKG KEGRFRGNLS

  361
h GKRVDFSART VITPDPNLSI DQVGVPRSIA ANMTFAEIVT PFNIDRLQEL VRRGNSQYPG AKYIIRDNGD RIDLRFHPKP SDLH..LQTG
m GKRVDFSART VITPDPNLSI DQVGVPRSIA ANMTFAEIVT PFNIDRLQEL VRRGNSQYPG AKYIIRDNGD RIDLRFHPKP SDLH..LQTG
d GKRVDFSART VITPDPNLRI DQVGVPRSIA QNLTFPELVT PFNIDRMQEL VRRGNSQYPG AKYIVRDNGE RIDLRFHPKS SDLH..LQCG
c GKRVDFSART VITADPNLPI DTVGVPRTIA QNLTFPEIVT PFNVDKLQEL VNRGDTQYPG AKYIIRENGA RVDLRYHPRA ADLH..LQPG
y GKRVDFSART VISGDPNLEL DQVGVPKSIA KTLTYPEVVT PYNIDRLTQL VRNGPNEHPG AKYVIRDSGD RIDLRYSKRA GDIQ..LQYG
s GKRVDFSSRT VISPDPNISI DEVGVPEIIA RTLTVPERIT PWNIEKLRQF VINGPDKWPG ANYVIRPDGR RIDLRYVKDR KELASTLAPG

  451
h YKVERHMCDG DIVIFNRQPT LHKMSMMGHR VRILPWSTFR LNLSVTTPYN ADFDGDEMNL HLPQSLETRA EIQELAMVPR MIVTPQSNRP
m YKVERHMCDG DIVIFNRQPT LHKMSMMGHR VRILPWSTFR LNLSVTTPYN ADFDGDEMNL HLPQSLETRA EIQELAMVPR MIVTPQSNRP
d YKVERHLRDD DLVIFNRQPT LHKMSMMGHR VKVLPWSTFR MNLSCTSPYN ADFDGDEMNL HVPQSMETRA EVENIHITPR QIITPQANKP
c YRVERHMKDG DIIVFNRQPT LHKMSMMGHR VKILPWSTFR MNLSVTSPYN ADFDGDEMNL HLPQSLETRA EIEEIAMVPR QLITPQANKP
y WKVERHIMDN DPVLFNRQPS LHKMSMMAHR VKVIPYSTFR LNLSVTSPYN ADFDGDEMNL HVPQSEETRA ELSQLCAVPL QIVSPQSNKP
s YVVERHLTDG DVVLFNRQPS LHRISMMAHR VRVLKGLTFR LNLLVCPPYN ADFDGDEMNL HVPQSEEAIA EAKEIMLVHK NIITPRYGGP

  541
h VMGIVQDTLT AVRKFTKRDV FLERGEVMNL LMFLSTWDGK VPQPAILKPR PLWTGKQIFS LIIPGHINCI RTHSTHPDDE DSGPYKHISP
m VMGIVQDTLT AVRKFTKRDV FLERGEVMNL LMFLSTWDGK VPQPAILKPR PLWTGKQIFS LIIPGHINCI RTHSTHPDDE DSGPYKHISP
d VMGIVQDTLT AVRKMTKRDV FITREQVMNL LMFLPTWDAK MPQPCILKPR PLWTGKQIFS LIIPGNVNMI RTHSTHPDEE DEGPYKWISP
c VMGIVQDTLC AVRMMTKRDV FIDWPFMMDL LMYLPTWDGK VPQPAILKPK PLWTGKQVFS LIIPGNVNVL RTHSTHPDSE DSGPYKWISP
y CMGIVQDTLC GIRKLTLRDT FIELDQVLNM LYWVPDWDGV IPTPAIIKPK PLWSGKQILS VAIPNGIHLQ RF........ DEGT.TLLSP
s IIGAAQDYIS GAYLLTVKTT LLTKEEAQQI L.GVADVKID LGEPAILAPR EYYTGKQVVS AFLPKDFNFH GQANV..SSG PRLCKNEDCP

  631
h GDTKVVVENG ELIMGILCKK SLGTSAG.SL VHISYLEMGH DITRLFYSNI QTVINNWLLI EGHTIGIGDS IADSKTYQDI QNTIKKAKQD
m GDTKVVVENG ELIMGILCKK SLGTSAG.SL VHISYLEMGH DITRLFYSNI QTVINNWLLI EGHTIGIGDS IADSKTYQDI QNTIKKAKQD
d GDTKVMVEHG ELIMGILCKK SLGTSAG.SL LHICFLELGH DIAGRFYGNI QTVINNWLLF EGHSIGIGDT IADPQTYNEI QQAIKKAKDD
c GDTKVIIEHG ELLSGIVCSK TVGKSAG.NL LHVVTLELGY EIAANFYSHI QTVINAWLIR EGHTIGIGDT IADQATYLDI QNTIRKAKQD
y KDNGMLIIDG QIIFGVVEKK TVGSSNG.GL IHVVTREKGP QVCAKLFGNI QKVVNFWLLH NGFSTGIGDT IADGPTMREI TETIAEAKKK
s HDSYVVIKNG ILLEGVFDKK AIGNQQPESI LHWLIKEYSD EYGKWLMDNL FRVFIRFVEL QGFTMRLEDV SLGDDVKKEI YNEIDRAKVE

  721
h VIEVIEKAHN NELEPTPGNT LRQTFENQVN RILNDARDKT GSSAQKSLSE YNNFKSMVVS GAKGSKINIS QVIAVVGQQN VEGKRIPFGF
m VIEVIEKAHN NELEPTPGNT LRQTFENQVN RILNDARDKT GSSAQKSLSE YNNFKSMVVS GAKGSKINIS QVIAVVGQQN VEGKRIPFGF
d VINVIQKAHN MELEPTPGNT LRQTFENKVN RILNDARDKT GGSAKKSLTE YNNLKAMVVS GSKGSNINIS QVIACVGQQN VEGKRIPYGF
c VVDVIEKAHN DDLEPTPGNT LRQTFENKVN QILNDARDRT GSSAQKSLSE FNNFKSMVVS GSKGSKINIS QVIACVGQQN VEGKRIPFGF
y VLDVTKEAQA NLLTAKHGMT LRESFEDNVV RFLNEARDKA GRLAEVNLKD LNNVKQMVMA GSKGSFINIA QMSACVGQQS VEGKRIAFGF
s VDNLIQKYKN GELEPIPGRT LEESLENYIL DTLDKLRSTA GDIASKYLDP FNFAYVMART GARGSVLNIT QMAAMLGQQS VRGERIKRGY
```

```
  811
h KHRTLPHFIK DDYGPESRGF VENSYLAGLT PTEFFFHAMG GREGLIDTAV KTAETGYIQR RLIKSMESVM VKYDATVRNS INQVVQLRYG
m KHRTLPHFIK DDYGPESRGF VENSYLAGLT PTEFFFHAMG GREGLIDTAV KTAETGYIQR RLIKSMESVM VKYDATVRNS INQVVQLRYG
d RKRTLPHFIK DDYGPESRGF VENSYLAGLT PSEFYFHAMG GREGLIDTAV KTAETGYIQR RLIKAMESVM VNYDGTVRNS VGQLIQLRYG
c RHRTLPHFIK DDYGPESKGF VENSYLAGLT PSEFFFHAMG GREGLIDTAV KTAETGYIQR RLIKAMESVM VNYDGTVRNS LAQMVQLRYG
y VDRTLPHFSK DDYSPESKGF VENSYLRGLT PQEFFFHAMG GREGLIDTAV KTAETGYIQR RLVKALEDIM VHYDNTTRNS LGNVIQFIYG
s MTRTLPHFKP YDISPEARGF IYSSFRTGLK PTELFFHAAG GREGLVDTAV RTSQSGYMQR RLINALSDLR AEYDGTVRSL YGEVIQVAYG

  901
h EDGLAGESVE FQNLATLKPS NKAFEKKFRF DYTNERALRR TLQEDLVKDV LSNAHIQNEL EREFERMRED REVLRVIFPT GDSKVVLPCN
m EDGLAGESVE FQNLATLKPS NKAFEKKFRF DYTNERALRR TLQEDLVKDV LSNAHIQNEL EREFERMRED REVLRVIFPT GDSKVVLPCN
d EDGLCGELVE FQNMPTVKLS NKSFEKRFKF DWSNERLMKK VFTDDVIKEM TDSSEAIQEL EAEWDRLVSD RDSLRQIFPN GESKVVLPCN
c EDGLDGMWVE NQNMPTMKPN NAVFERDFRM DLTDNKFLRK NYSEDVVREI QESEDGISLV ESEWSQLEED RRLLRKIFPR GDAKIVLPCN
y EDGMDAAHIE KQSLDTIGGS DAAFEKRYRV DLLNTDHTLD PSLLESGSEI LGDLKLQVLL DEEYKQLVKD RKFLREVFVD GEANWPLPVN
s DDGVFPMYSA HGKTVDV... NRIFER.... .......... .....VVGWK T....MENVI DE......KD KSYLEE.... ..........

  991
h LLRMIWNAQK IFHINPRLPS DLHPIKVVEG VKELSKKLVI VNGDDPLSRQ AQENATLLFN IHLRSTLCSR RMAEEFRLSG EAFDWLLGEI
m LLRMIWNAQK IFHINPRLPS DLHPIKVVEG VKELSKKLVI VNGDDPLSRQ AQENATLLFN IHLRSTLCSR RMAEEFRLSG EAFDWLLGEI
d LQRMIWNVQK IFHINKRLPT DLSPIRVIKG VKTLLERCVI VTGNDRISKQ ANENATLLFQ CLIRSTLCTK YVSEEFRLST EAFEWLVGEI
c LQRLIWNAQK IFKVDLRKPV NLSPLHVISG VRELSKKLII VSGNDEISKQ AQYNATLLMN ILLRSTLCTK NMCTKSKLNS EAFDWLLGEI
y IRRIIQNAQQ TFHIDHTKPS DLTIKDIVLG VKDLQENLLV LRGKNEIIQN AQRDAVTLFC CLLRSRLATR RVLQEYRLTK QAFDWVLSNI
s .......... ....KVKQAS NILPQKIVED LKNLISNKEV LVTRDEIDK. .......... .......... .......... .IFDLAIKE.

  1081
h ESKFNQAIAH PGEMVGALAA QSLGEPATQM TLNTFHYAGV SAK..NVTLG VPRLKELINI SKKPK...TP SLTVFLLGQS ARDAERAKDI
m ESKFNQAIAH PGEMVGALAA QSLGEPATQM TLNTFHYAGV SAK..NVTLG VPRLKELINI SKKPK...TP SLTVFLLGQS ARDAERAKDI
d ETRFQQAQAN PGEMVGALAA QSLGEPATQM TLNTFHFAGV SSK..NVTLG VPRLKEIINI SKKPK...AP SLTVFLTGGA ARDAEKAKNV
c ESRFQQAIAQ PGEMVGALAA QSLGEPATQM TLNTFHYAGV SAK..NVTLG VPRLKEIINV SKTLK...TP SLTVFLTGAA AKDPEKAKDV
y EAQFLRSVVH PGEMVGVLAA QSIGEPATQM TLNTFHFAGV ASK..KVTSG VPRLKEILNV AKNMK...TP SLTVYLEPGH AADQEQAKLI
s ...YSEGLIA PGEAIGIVAA QSVGEPGTQM TLRTFHFAGI ..RELNVTLG LPRLIEIVD. AK..KVPSTP MMTIYLTDEY KHDKEKALEV

  1171
h LCRLEHTTLR KVTANTAIYY DPNPQSTVVA EDQEWVNVYY EMPDFDVARI ....SPWLLR VELDRKHMTD RKLTMEQIAE KINAGFGDDL
m LCRLEHTTLR KVTANTAIYY DPNPQSTVVA EDQEWVNVYY EMPDFDVARI ....SPWLLR VELDRKHMTD RKLTMEQIAE KINAGFGDDL
d LCRLEHTTLR KVTANTAIYY DPDPQRTVIS EDQEFVNVYY EMPDFDPTRI ....SPWLLR IELDRKRMTD KKLTMEQIAE KINVGFGEDL
c LCKLEHTTLK KVTCNTAIYY DPDPKNTVIA EDEEWVSIFY EMPDHDLSRT ....SPWLLR IELDRKRMVD KKLTMEMIAD RIHGGFGNDV
y RSAIEHTTLK SVTIASEIYY DPDPRSTVIP EDEEIIQLHF SLLDEEAEQS FDQQSPWLLR LELDRAAMND KDLTMGQVGE RIKQTFKNDL
s ARKLEYTKIE NVVSSTSI.. DIASMSII.. .......... .......... .......... LQLDNEMLKD KGVTVDDVKK AINRLKLGE.

  1261
h NCIFNDDNAE KLVLRIRIMN SDENKMQEEE EVVDKMDDDV FLRCIESNML TDMTLQGIEQ ISKVYMHLPQ TDNKKKIIIT EDGEFKALQE
m NCIFNDDNAE KLVLRIRIMN SDENKMQEEE EVVDKMDDDV FLRCIESNML TDMTLQGIEQ ISKVYMHLPQ TDNKKKIIIT EDGEFKALQE
d NCIFNDDNAD KLVLRIRIMN NEENKFQDED EAVDKMEDDM FLRCIEANML SDMTLQGIEA IGKVYMHLPQ TDSKKRIVIT ETGEFKAIGE
c HTIYTDDNAE KLVFRLRIAG EDKGEAQEEQ ..VDKMEDDV FLRCIEANML SDLTLQGIPA ISKVYMNQPN TDDKKRIIIT PEGGFKSVAD
y FVIWSEDNDE KLIIRCRVVR PKSLDAETE. .....AEEDH MLKKIENTML ENITLRGVEN IERVVMM... ..KYDRKVPS PTGEYVKEPE
s FVI...DESE GNTLNISFAN IDSIAA.... .......... .LFKLRDKIL .NTKIKGIKG IKRAIVQ... .......... .....KKGDE

  1351
h WILETDGVSL MRVLSEKDVD PVRTTSNDIV EIFTVLGIEA VRKALERELY HVISFDGSYV NYRHLALLCD TMTCRGHLMA ITRHGVNRQD
m WILETDGVSL MRVLSEKDVD PVRTTSNDIV EIFTVLGIEA VRKALERELY HVISFDGSYV NYRHLALLCD TMTCRGHLMA ITRHGVNRQD
d WLLETDGTSM MKVLSERDVD PIRTSSNDIC EIFQVLGIEA VRKSVEKEMN AVLQFYGLYV NYRHLALLCD VMTAKGHLMA ITRHGINRQD
c WILETDGTAL LRVLSERQID PVRTTSNDIC EIFEVLGIEA VRKAIEREMD NVISFDGSYV NYRHLALLCD VMTAKGHLMA ITRHGINRQE
y WVLETDGVNL SEVMTVPGID PTRIYTNSFI DIMEVLGIEA GRAALYKEVY NVIASDGSYV NYRHMALLVD VMTTQGGLTS VTRHGFNRSN
s YIILTDGSNL SGVLSVKGVD IAKVETNNIR EIEEVFGIEA AREIIIREIS KVLAEQGLDV DMRHILLVAD VMTRTGVVRQ IGRHGVTGEK

  1441
h TGPLMKCSFE ETVDVLMEAA AHGESDPMKG VSENIMLGQL APAGTGCFDL LLDAEKCKYG
m TGPLMKCSFE ETVDVLMEAA AHGESDPMKG VSENIMLGQL APAGTGCFDL LLDAEKCKYG
d TGALMRCSFE ETVDVLMDAA AHAETDPMRG VSENIIMGQL PKMGTGCFDL LLDAEKCRFG
c VGALMRCSFE ETVDILMEAA VHAEEDPVKG VSENIMLGQL ARCGTGCFDL VLDVEKCKYG
y TGALMRCSFE ETVEILFEAG ASAELDDCRG VSENVILGQM APIGTGAFDV MIDEESLVKY
s NSVLARAAFE VTVKHLLDAA ARGDVEEFKG VVENIIIGHP IKLGTGMVEL TMRPILR...
```

Sequence alignment of human (**h**) RPB1 section 1–1483 with M. musculus (**m**), D. melanogaster (**d**), C. elegans (**c**), S. cerevisiae (**y**) and S. shibatae (**s**) RNAP/RNAPII. High (red), Low (blue) and Null (black) conservation scores are indicated. Sequence alignments were computed with Blossum62 consensus scores, and opening/extension gap penalties of 12/2. The conservation of K/R/Q amino acid combinations was recalculated according to Risler consensus scores. Sections RPB1 938–947 and 1110–1143 were manually re-aligned to conform to structural data.

Table S7. RPB2 subunit conservation.

```
  1
h .MYDADEDMQ YD.......E DDDEITPDLW QEACWIVISS YFDEKGLVRQ QLDSFDEFIQ MSVQRIVEDA PPIDLQAEAQ HASGEVEEPP
m .MYDADEDMQ YD.......E DDDEITPDLW QEACWIVISS YFDEKGLVRQ QLDSFDEFIQ MSVQRIVEDA PPIDLQAEAQ HASGEVEEPP
d MMYDNEEELY EE.......E NAEEISHELW QEACWIVINA YFDEKGLVRQ QLDSFDEFIQ MSVQRIVEDS PAIELQAEAQ HTSGEVETPP
c .MYDDEDEMV NDPMDGDYID DSDEISAEAW QEACWVVISA YFDEKGLVRQ QLDSFDEFVQ MNVQRIVEDS PPVELQSENQ HLGTDMENPA
y MSDLANSEKY YD....EDPY GFEDESAPIT AEDSWAVISA FFREKGLVSQ QLDSFNQFVD YTLQDIICED STLILEQLAQ HTTESDNISR
s ......... .......... .MNELSSNLS IDERWKVIEA YFKSKGLVRQ HLDSYNDFVR NKLQEIIDEQ GEIPTEI... .........P

  91
h RYLLKFEQIY LSKPTHWERD GAPSPMMPNE ARLRNLTYSA PLYVDITKTV .......... ....IKEGEE QLQTQHQKTF IGKIPIMLRS
m RYLLKFEQIY LSKPTHWERD GAPSPMMPNE ARLRNLTYSA PLYVDITKTV .......... ....IKEGEE QLQTQHQKTF IGKIPIMLRS
d RFSLKFEQIY LSKPTHWEKD GSPSPMMPNE ARLRNLTYSA PLYVDITKTK .......... ....NVEGLD PVETQHQKTF IGKIPIMLRS
c KFSLKFNQIY LSKPTHWEKD GAPMPMMPNE ARLRNLTYAS PLYVDITKVV .......... ....TRD.DS ATEKVYDKVF VGKVPVMLRS
y KYEISFGKIY VTKPMVNESD GVTHALYPQE ARLRNLTYSS GLFVDVKKRT YEAIDVPGRE LKYELIAEES EDDSESGKVF IGRLPIMLRS
s GLKVRLGKIR IGKPRVRESD RGEREISPME ARLRNLTYAA PLWLTMIPV. .......... ........EN NIEAEPEEVY IGDLPIMLKS

  181
h TYCLLNGLTD RDLCELNECP LDPGGYFIIN GSEKVLIAQE KMATNTVYVF AKK..DSKYA YTGECRSCLE NSSRPTSTIW VSMLARGGQG
m TYCLLNGLTD RDLCELNECP LDPGGYFIIN GSEKVLIAQE KMATNTVYVF AKK..DSKYA YTGECRSCLE NSSRPTSTIW VSMLARGGQG
d TYCLLSQLTD RDLTELNECP LDPGGYFIIN GSEKVLIAQE KMATNTVYVF SMK..DGKYA FKTEIRSCLE HSSRPTSTLW VNMMARGSQN
c SYCMLSNMTD RDLTELNECP LDPGGYFVIN GSEKVLIAQE KMATNTVYVF SMK..DGKYA FKTECRSCLE NSSRPTSTMW VNMLARGGGG
y KNCYLSEATE SDLYKLKECP FDMGGYFIIN GSEKVLIAQE RSAGNIVQVF .KKAAPSPIS HVAEIRSALE KGSRFISTLQ VKLYGREGSS
s AIDPISQYTL DKLIEIGEDP KDPGGYFIVN GSERVIVTQE DLAPNRVLVD TGKT.GSNIT HTAKIISSTA GYRVPVT... IERLKDGT..

  271
h AKKSAIGQRI VATLPYIKQE VPIIIVFRAL GFVSDRDILE HIIYDFEDPE MMEMVKPSLD EAFVIQEQNV ALNFIGSRGA KPGVTKEKRI
m AKKSAIGQRI VATLPYIKQE VPIIIVFRAL GFVSDRDILE HIIYDFEDPE MMEMVKPSLD EAFVIQEQNV ALNFIGSRGA KPGVTKEKRI
d IKKSAIGQRI IAILPYIKQE IPIMIVFRAL GFVADRDILE HIIYDFDDPE MMEMVKPSLD EAFVVQEQNV ALNFIGARGA RPGVTKDKRI
c GKKTAMGQRI IGILPYIKQE IPIMIVFRAL GFVSDRDILG HIIYDFNDPE MMEMVKPSLD EAFVIQEQNV ALNFIGARGA KPGVTREQRI
y ART......I KATLPYIKQD IPIVIIFRAL GIIPDGEILE HICYDVNDWQ MLEMLKPCVE DGFVIQDRET ALDFIGRRGT ALGIKKEKRI
s .........F HVSFPAVPGK IPFVILMRAL GILTDRDIVY AVSLD...PE IQNELFPSLE QASSIANVDD ALDFIGSR.V AIGQKRENRI

  361
h KYAKEVLQKE MLPHVGVSDF CETKKAYFLG YMVHRLLLAA LGRRELDDRD HYGNKRLDLA GPLLAFLFRG MFKNLLKEVR IYAQKFIDRG
m KYAKEVLQKE MLPHVGVSDF CETKKAYFLG YMVHRLLLAA LGRRELDDRD HYGNKRLDLA GPLLAFLFRG MFKNLLKEVR IYAQKFIDRG
d KYAKEILQKE MLPHVGVSDF CETKKAYFLG YMVHRLLLAS LGRRELDDRD HYGNKRLDLA GPLLAFLFRG LFKNLMKEVR MYTQKFIDRG
c KYAREILQKE LLPHVGVSEH CETKKAFFIG YMVHRLLLAA LGRRELDDRD HIGNKRLDLA GPLLAFLFRS LFRNLLKEMR MTAQKYINKN
y QYAKDILQKE FLPHITQLEG FESRKAFFLG YMINRLLLCA LDRKDQDDRD HFGKKRLDLA GPLLAQLFKT LFKKLTKDIF RYMQRTVEEA
s EKAQQIIDKY FLPHLGTSAD DRRKKAYYLA YAISKVIELY LGRREPDDKD HYANKRLRLA GDLFASLFRV AFKAFVKDLT YQLEKSKVRG

  451
h KDFNLELAIK TRIISDGLKY SLATGNWGDQ KKAHQARAGV SQVLNRLTFA STLSHLRRLN SPIGRDGKLA K..PRQLHNT LWGMVCPAET
m KDFNLELAIK TRIISDGLKY SLATGNWGDQ KKAHQARAGV SQVLNRLTFA STLSHLRRLN SPIGRDGKLA K..PRQLHNT LWGMVCPAET
d KDFNLELAIK TNIITDGLRY SLATGNWGDQ KKAHQARAGV SQVLNRLTFA STLSHLRRVN SPIGRDGKLA K..PRQLHNT LWGMLCPAET
c DDFALDVCVK TSTITRGLTY SLATGNWGDQ KKAHQSRAGV SQVLNRLTYT ATLSHLRRAN SPIGREGKLA K..PRQLHNT QWGMVCPAET
y HDFNMKLAIN AKTITSGLKY ALATGNWGEQ KKAMSSRAGV SQVLNRYTYS STLSHLRRTN TPIGRDGKLA K..PRQLHNT HWGLVCPAET
s RKLALKALVR PDIVTERIRH ALATGNW... ...VGGRTGV SQLLDRTNWL SMLSHLRRVI SSLAR.GQ.P NFEARDLHGT QWGRMCPFET

  541
h PEGHAVGLVK NLALMAYISV GSQPSPILEF LEEWSMENLE EISPAAIADA .........T KIFVNGCWVG IHKDPEQLMN TLRKLRRQMD
m PEGHAVGLVK NLALMAYISV GSQPSPILEF LEEWSMENLE EISPAAIADA .........T KIFVNGCWVG IHKDPEQLMN TLRKLRRQMD
d PEGAAVGLVK NLALMAYISV GSQPSPILEF LEEWSMENLE EIAPSAIADA .........T KIFVNGCWVG IHRDPEQLMA TLRKLRRQMD
c PEGQAVGLVK NLALMAYISV GSLPEPILEF LEEWSMENLE EVSPSAIADA .........T KIFVNGAWVG IHREPDQLMT TLKKLRRQMD
y PEGQACGLVK NLSLMSCISV GTDPMPIITF LSEWGMEPLE DYVPHQSPDA .........T RVFVNGVWHG VHRNPARLME TLRTLRRKGD
s PEGPNSGLVK NLALMAQIAV GINEKIVEKT LYEMGVVPVE EVIRRVTEGG EDQNEYLKWS KVILNGRLVG YYRDGEELAK KIRERRRKGE

  631
h IIVSEVSMIR DIRE..REIR IYTDAGRICR PLLIVEK... ...QKLLLKK RHIDQLKER. .........E YNNYSWQDLV ASGVVEYIDT
m IIVSEVSMIR DIRE..REIR IYTDAGRICR PLLIVEK... ...QKLLLKK RHIDQLKER. .........E YNNYSWQDLV ASGVVEYIDT
d IIVSEVSMIR DIRD..REIR IYTDAGRICR PLLIVEN... ...GSLLLKK THVEMLKER. .........D YNNYSWQVLV ASGVVEYIDT
c IIVSEVSMVR DIRD..REIR IYTDAGRVCR PLLIVEN... ...QKLALKK RHIDQLKEAA ........DE ANKYTWSDLV GGGVVELIDS
y IN.PEVSMIR DIRE..KELK IFTDAGRVYR PLFIVEDDES LGHKELKVRK GHIAKLMATE YQDIEGGFED VEEYTWSSLL NEGLVEYIDA
s IS.DEVNVGH IVTDFINEVH VNCDSGRVRR PLIIVSN... ...GNPLVTR EDIEKL.... .........D SGSITFDDLV RQGKIEYLDA

  721
h EEEENAYVAL EPSDL..... .......... .........T PEHTHLEIWS PAILGITASI IPYPEHNQSP RNTYQSAMAK QAMGVYITNF
m LEEETVMLAM TPDDL..... .......... ..QEKEVAYC STYTHCEIHP SMILGVCASI IPFPDHNQSP RNTYQSAMGK QAMGVYITNF
d LEEETVMIAM SPYDL..... .......... .KQDKDYAYC TTYTHCEIHP AMILGVCASI IPFPDHNQSP RNTYQSAMGK QAMGVYITNF
c MEEETSMIAM MPEDL..... .......... ....RSGGYC DTHTHCEIHP AMILGVCASI IPFPDHNQSP RNTYQSAMGK QAMGVYTTNF
y EEEESILIAM QPEDLEPAEA NEENDLDVDP AKRIRVSHHA TTFTHCEIHP SMILGVAASI IPFPDHNQSP RNTYQSAMGK QAMGVFLTNY
s EEEENAYVAL EPSDL..... .......... .........T PEHTHLEIWS PAILGITASI IPYPEHNQSP RNTYQSAMAK QALGLYAANY
```

```
  811
h HVRMDTLAHV LYYPQKPLVT TRSMEYLRFR ELPAGINSIV AIASYTGYNQ EDSVIMNRSA VDRGFFRSVF YRSYKEQESK KGFDQEEVFE
m HVRMDTLAHV LYYPQKPLVT TRSMEYLRFR ELPAGINSIV AIASYTGYNQ EDSVIMNRSA VDRGFFRSVF YRSYKEQESK KGFDQEEVFE
d HVRMDTLAHV LYYPMKPLVT TRSMEYLRFR ELPAGINSIV AILCYTGYNQ EDSVILNASA VERGFFRSVF YRSYKDSENK RVGDQEENFE
c HVRMDTLAHV LYYPQKPLVT TRSMEYLRFN ELPAGINAIV AILSYSGYNQ EDSVIMNNSA IDRGLFRSVF YRSYRDNEAN LDNANEELIE
y NVRMDTMANI LYYPQKPLGT TRAMEYLKFR ELPAGQNAIV AIACYSGYNQ EDSMIMNQSS IDRGLFRSLF FRSYMDQEKK YGMSITETFE
s QLRTDTRAHL LHYPQRPLVQ TRALDIIGYT NRPAGNNAIL AVISFTGYNM EDSIIMNRSS VERGMYRSTF FRLYSTEEVK YPGGQEDKIV

  901
h KPTRETCQGM RHAIYDKLDD DGLIAPGVRV SGDDVIIGKT VTLPENEDEL ESTNRRYTKR DCSTFLRTSE TGIVDQVMVT LNQEGYKFCK
m KPTRETCQGM RHAIYEKLDD DGLIAPGVRV SGDDVIIGKT VTLPENEDEL ESTNRRYTKR DCSTFLRTSE TGIVDQVMVT LNQEGYKFCK
d KPHRGTCQGM RNAHYDKLDD DGIIAPGIRV SGDDVVIGKT ITLPENDDEL DSNTKRFSKR DASTFLRNSE TGIVDQVMLT LNSEGYKFCK
c KPTREKCSGM RHSLYDKLDE DGIISPGMRV SGDDVIIGKT VALPDIDDDL DASGKKYPKR DASTFLRSSE TGIVDQVMLS LNSDGNKFVK
y KPQRTNTLRM KHGTYDKLDD DGLIAPGVRV SGEDVIIGKT TPISPDEEEL GQRTAYHSKR DASTPLRSTE NGIVDQVLVT TNQDGLKFVK
s MPEPGVRGYK GKEYYRLLED NGVVSPEVEV KGGDVLIGKV SP.PRFLQEF KELSPEQAKR DTSIVTRHGE MGIVDLVLIT ETAEGNKLVK

  991
h IRVRSVRIPQ IGDKFASRHG QKGTCGIQYR QEDMPFTCEG ITPDIIINPH AIPSRMTIGH LIECLQGKVS ANKGEIGDAT PFNDAVNVQK
m IRVRSVRIPQ IGDKFASRHG QKGTCGIQYR QEDMPFTCEG ITPDIIINPH AIPSRMTIGH LIECLQGKVS ANKGEIGDAT PFNDAVNVQK
d IRVRSVRIPQ IGDKFASRHG QKGTCGIQYR QEDMAFTCEG LAPDIIINPH AIPSRMTIGH LIECLQGKLG SNKGEIGDAT PFNDAVNVQK
c IRMRSVRLPQ IGDKFASRHG QKGTMGIMYR QEDMPFTAEG LTPDIIINPH AVPSRMTIGH LIECLQGKLS ANKGEIGDAT PFNDTVNVQK
y VRVRTTKIPQ IGDKFASRHG QKGTIGITYR REDMPFTAEG IVPDLIINPH AIPSRMTVAH LIECLLSKVA ALSGNEGDAS PFTD.ITVEG
s VRVRDLRIPS IGDKFASRHG QKGVIGMLIP QVDMPYTVKG VVPDVILNPH ALPSRMTLGQ IMEGIAGKYA ALSGNIVDAT PFYK.TPIEQ

  1081
h ISNLLSDYGY HLRGNEVLYN GFTGRKITSQ IFIGPTYYQR LKHMVDDKIH SRARGPIQIL NRQPMEGRSR DGGLRFGEME RDCQIAHGAA
m ISNLLSDYGY HLRGNEVLYN GFTGRKITSQ IFIGPTYYQR LKHMVDDKIH SRARGPIQIL NRQPMEGRSR DGGLRFGEME RDCQIAHGAA
d ISTFLQEYGY HLRGNEVMYN GHTGRKINAQ VFLGPTYYQR LKHMVDDKIH SRARGPVQIL VRQPMEGRAR DGGLRFGEME RDCQISHGAA
c ISGLLCEYGY HLRGNEVMYN GHTGKKLTTQ IFFGPTYYQR LKHMVDDKIH SRARGPIQMM NRQPMEGRAR DGGLRFGEME RDCQISHGAT
y ISKLLREHGY QSRGFEVMYN GHTGKKLMAQ IFFGPTYYQR LRHMVDDKIH ARARGPMQVL TRQPVEGRSR DGGLRFGEME RDCMIAHGAA
s LQNEILKYGY LPDATEVTYD GRTGQKIKSR IYFGVVYYQK LHHMVADKIH ARARGPVQIL TRQPTEGRAR EGGLRFGEME RDCLIGFGTA

  1171
h QFLRERLFEA SDPYQVHVCN LCGIM.AIAN TRTHTYECRG CRNKTQISLV RMPYACKLLF QELMSMSIAP RMMSV..... ........
m QFLRERLFEA SDPYQVHVCN LCGIM.AIAN TRTHTYECRG CRNKTQISLV RMPYACKLLF QELMSMSIAP RMMSV..... ........
d QFLRERLFEV SDPYRVHICN FCGLI.AIAN LRNNTFECKG CKNKTQISQV RLPYAAKLLF QELMSMNIAP RLMVT..... ........
c QFLRERLFEV SDPYHVYVCN NCGLI.VVAN LRTNSFECKA CRNKTQVSAV RIPYACKLLF QELMSMSIAP RLMVKPRQSK RSKHQSEA
y SFLKERLMEA SDAFRVHICG ICGLMTVIAK LNHNQFECKG CDNKIDIYQI HIPYAAKLLF QELMAMNITP RLYTDRSRDF ........
s MLLKDRLLDN SDRTTIYVCD QCGYI.GWYD KNKNKYVCPI HGDKSNLFPV TVSYAFKLLI QELMSMIISP RLILEDRVGL SGGKGNE.
```

Sequence alignment of human (**h**) RPB2 subunit with M. musculus (**m**), D. melanogaster (**d**), C. elegans (**c**), S. cerevisiae (**y**) and S. shibatae (**s**) RNAP/RNAPII. High (red), Low (blue) and Null (black) conservation scores are indicated. Sequence alignments were computed with Blossum62 consensus scores, and opening/extension gap penalties of 12/2. The conservation of K/R/Q amino acid combinations was recalculated according to Risler consensus scores. Sections RPB2 207–223 and 491–498 were manually re-aligned to conform to structural data.
